# Scaling-up and proteomic analysis reveals photosynthetic and metabolic insights toward prolonged H_2_ photoproduction in Chlamydomonas *hpm91* mutant lacking proton gradient regulation 5 (PGR5)

**DOI:** 10.1007/s11120-022-00945-4

**Published:** 2022-08-16

**Authors:** Peng Liu, De-Min Ye, Mei Chen, Jin Zhang, Xia-He Huang, Li-Li Shen, Ke-Ke Xia, Xiao-Jing Xu, Yong-Chao Xu, Ya-Long Guo, Ying-Chun Wang, Fang Huang

**Affiliations:** 1 Photosynthesis Research Center, Key Laboratory of Photobiology, Institute of Botany, Chinese Academy of Sciences, Beijing, 100093 China; 2 State Key Laboratory of Molecular Developmental Biology, Institute of Genetics and Developmental Biology, Chinese Academy of Sciences, Beijing, 100101 China; 3 BGI-Shenzhen, Shenzhen, 518083 China; 4 State Key Laboratory of Systematic and Evolutionary Botany, Institute of Botany, Chinese Academy of Sciences, Beijing, 100093 China; 5 University of Chinese Academy of Sciences, Beijing, 100049 China

**Keywords:** *C. reinhardtii*; *hpm91* mutant, Scale-up H_2_ production, ROS, Quantitative proteomics, *hpm91*-derived mutant

## Abstract

Clean and sustainable H_2_ production is crucial to a carbon–neutral world. H_2_ generation by *Chlamydomonas reinhardtii* is an attractive approach for solar-H_2_ from H_2_O. However, it is currently not large-scalable because of lacking desirable strains with both optimal H_2_ productivity and sufficient knowledge of underlying molecular mechanism. We hereby carried out extensive and in-depth investigations of H_2_ photoproduction of *hpm91* mutant lacking PGR5 (Proton Gradient Regulation 5) toward its up-scaling and fundamental mechanism issues. We show that *hpm91* is at least 100-fold scalable (up to 10 L) with continuous H_2_ collection of 7287 ml H_2_/10L-HPBR in averagely 26 days under sulfur deprivation. Also, we show that *hpm91* is robust and active during sustained H_2_ photoproduction, most likely due to decreased intracellular ROS relative to wild type. Moreover, we obtained quantitative proteomic profiles of wild type and *hpm91* at four representing time points of H_2_ evolution, leading to 2229 and 1350 differentially expressed proteins, respectively. Compared to wild type, major proteome alterations of *hpm91* include not only core subunits of photosystems and those related to anti-oxidative responses but also essential proteins in photosynthetic antenna, C/N metabolic balance, and sulfur assimilation toward both cysteine biosynthesis and sulfation of metabolites during sulfur-deprived H_2_ production. These results reveal not only new insights of cellular and molecular basis of enhanced H_2_ production in *hpm91* but also provide additional candidate gene targets and modules for further genetic modifications and/or in artificial photosynthesis mimics toward basic and applied research aiming at advancing solar-H_2_ technology.

**Supplementary Information:**

The online version contains supplementary material available at 10.1007/s11120-022-00945-4.

## Introduction

Hydrogen (H_2_) derived from H_2_O is a clean and versatile energy carrier that could be obtained sustainably through sunlight-driven chemical and biological means such as photocatalysis and algal photoproduction (Bayro-Kaiser and Nelson 2017; Nishiyama et al. 2021). Under anaerobic condition, H_2_ photoproduction occurs in microalgae such as *Chlamydomonas reinhardtii* (henceforth referred to as *Chlamydomonas*) via two distinct light-driving pathways dependent on the activities of photosystems and (Fe–Fe) hydrogenases (Chochois et al. 2009; Fouchard et al. 2005). H_2_ formation is through the catalytic activity of (Fe–Fe) hydrogenases (Forestier et al. 2003) induced under anoxia, using mainly photosynthetic electrons on the acceptor side of photosystem I (PSI) in reduction of protons into H_2_. As a result, renewable H_2_ production is achieved in the organisms using solar energy and electrons derived from photosynthetic water-splitting reaction. However, H_2_ evolution by wild-type Chlamydomonas strains is only transient with small amounts, probably due to high sensitivity of the hydrogenases to O_2_ released from PSII (Ghirardi 2015). Significant progress was made mainly by Melis et al. (Melis et al. 2000) and Kruse et al. (Kruse et al. 2005) with development of the sulfur-deprivation method for sustained H_2_ evolution and establishment of *stm6* mutant that produces 540 ml of H_2_ /1L culture up to 14 days under such condition, respectively.

Making use of genomic sequence information (Merchant et al. 2007) and various tools of systems biology (Nguyen et al. 2008; Matthew et al. 2009; Chen et al. 2010; Toepel et al. 2013, numerous Chlamydomonas mutants with increased H_2_ production have been obtained. Several of them are reasonably well studied such as the truncated light-harvesting antenna mutant *(tla1),* D1 mutant (Kosourov et al. 2011; Scoma et al. 2012), and the *pgr* mutants named as *pgrl1* (proton gradient regulation like 1)*, pgr5* (proton gradient regulation 5), and *hpm91* (Tolleter et al. 2011; Steinbeck et al. 2015; Chen et al. 2016; 2019). The *pgr* mutants appeared highly favorable because their target genes (*Pgrl1*, *Pgr5*) are involved in the PGR5-dependent path of photosynthetic cyclic electron flow (CEF), which is the major branch of CEF (Takahashi et al. 2013; Schwenkert et al. 2022). Inverse relationship of CEF and H_2_ evolution was firstly revealed in the study of *stm6* mutant lacking MOC1 (an assembly factor of the mitochondrial respiratory chain) (Schonfeld et al. 2004; Kruse et al. 2005) as well as the observation of increased H_2_ production upon addition of the CEF inhibitor antimycin A (Antal et al. 2009). More experimental evidence was also obtained via analysis of *pgr (pgrl1, pgr5, hpm91)* as well as *fnr* (ferredoxin-NADPH reductase) mutants (Yacoby et al. 2011; Sun et al. 2013). PGRL1 and FNR have been identified in the CEF supercomplex (Iwai et al. 2010) and PGR5 is known being an important protein involved in the CEF branch (Munekage et al. 2002; Suorsa et al. 2012), for which the mechanistic mode is currently not completely understood (Schwenkert et al. 2022).

Remarkably, the *hpm91* mutant sustains H_2_ production for 25 days with 30-fold yield increase relative to wild type (Chen et al. 2016). Although a negative correlation was already revealed between PGR5 levels and H_2_ production, and the prolonged H_2_ evolution was mainly attributed to the enhanced anti-ROS capability protecting the photosynthetic electron transport chain from photooxidative damage, questions arise as (i) whether the phenotype of *hpm91* is stable in large scale setting-ups? (ii) how intracellular ROS content of *hpm91* is altered during sulfur-deprived H_2_ photoproduction? (iii) what are the proteomic characteristics of *hpm91* under such conditions; (iv) is it possible to obtain high H_2_-producing mutants excess to *hpm91*? We report here the performance of *hpm91* in a large scale (up to 10 L) H_2_-photobioreactor (HPBR) systems. We also describe insightful findings of *hpm91* based on quantitative proteomic analysis. We highlight the *hpm91* as a valuable algal strain not only for basic research of understanding the molecular mechanisms of bio-H_2_ photoproduction but also for development of economically viable solar-powered H_2_ production systems.

## Materials and methods

### Algal cultivation and H_2_ photoproduction

Wild-type *Chlamydomonas* strain CC400 was purchased from the Chlamydomonas Center (www.Chlamy.org) and the mutant strain *hpm91* was isolated in our laboratory and previously reported (Chen et al. 2016; 2019). Genetic analysis and phenotypic rescue of several fully complemented strains as well as immunoblot detection suggest that the loss of *Pgr5* gene is responsible for the H_2_-overproducing phenotype of *hpm91* (Chen et al. 2016).

H_2_ production was induced using sulfur-deprivation method (Melis et al. 2000) with minor modifications (Chen et al. 2010; Sun et al. 2013). Algal cells were grown in TAP medium (Gorman and Levine 1965) at 25 °C under continuous light (60 µmol photons m^−2^ s^−1^) until mid-exponential phase. Cells were pelleted and washed once with sulfur-depleted TAP medium followed by resuspending in the medium with desired cell density of 20- (for 10L-HPBR) and 25 μg ml^−1^ chlorophyll (*a* and *b*) (Arnon 1949), respectively. H_2_ evolution was achieved via transferring the culture into small H_2_ photobioreactor (100 ml, light path 4.5 cm) for biochemical analysis and large gas-tight glass bottles (large HPBR, upto 10 L; light path 10 cm for 1-3L and 22 cm for 10L, respectively) connected by a Teflon tube to storage glass cylinder for scaling-up studies followed by magnetic-bar-stirring cultivation at the same condition mentioned above (for small HPBR) or upto 230 µmol photons m^−2^ s^−1^ (for large HPBR), respectively. H_2_ gas accumulated in the headspace of the small HPBR was measured by gas chromatograph (GC-2014, Shimadzu, Japan) in the way as previously described (Sun et al. 2013; Zhao et al. 2013; Chen et al. 2016; 2019). The evolved H_2_ gas from the headspace of large HPBR was collected in inverted graduated cylinders and measured by the displacement of water (Melis et al. 2000).

### Intracellular ROS and cell growth analysis

Intracellular ROS content was determined using a MoFlo XDP high-speed flow cytometer (Beckman-Coulter, Inc. USA) by following the manufacturer’s instructions. Samples were prepared in an anoxia workstation (Longyao, LAI-3 T; Shanghai, China) as (Chen et al. 2019). Cells were incubated with 10 mM H_2_DCFDA at 25 °C for 30 min in dark then examined by the flow cytometer. For detection of DCF green fluorescence, wavelength of excitation at 488 nm and emission at 510 to 550 nm was used. Rosup provided by a ROS assay kit (Beyotime Institute of Biotechnology, Haimen, China) was used as a positive control. Average fluorescence intensity of DCF from 3 × 10^5^ cells was recorded and data acquisition and analysis were carried out using Summit 5.2 software (Beckman-Coulter, Inc. USA). Morphology of algal cells was examined and photographed with a differential interference contrast microscopy (DIC) (Leica DM4500, Germany). Culture density were determined by cell counting using a hemocytometer.

### iTRAQ proteomics and data analysis

Protein extraction and sample preparation for iTRAQ labeling was done as described (Chen et al. 2010) with minor modifications (Ge et al. 2017). Frozen cells suspended in ice-cold extraction buffer were disrupted with glass beads (diameter 150–212 μm, Sigma) via vortexing 30 s/5 cycles/each with 1-min break on ice. After removed unbroken cells and insoluble debris, total proteins in the supernatant were precipitated with ice-cold 10% trichloroacetic acid (TCA) in acetone at − 20 °C. Protein pellets were washed with acetone by centrifugation and vacuum-dried. The proteins were resolubilized with 4% sodium dodecyl sulfate (SDS) in 0.1 M Tris–HCl, pH 7.6. Protein concentration was determined using BCA protein assay kit (Thermo Scientific, Rockford, IL).

Trypsin digestion of proteins was performed using the filter-aided sample preparation (FASP) method with slight modifications (Wisniewski et al. 2009). The resulting tryptic peptides were labeled with 8-plex iTRAQ reagents (AB Sciex Inc., MA, USA) alternatively by following manufacturer's manual. The iTRAQ labeled samples were mixed together with equal ratios in amount, and the mixture was concentrated with a SpeedVac in subjection for fractionation by HPLC (Waters, e2695 separations) coupled with a phenomenex gemini-NX 5u C18 column (250 × 3.0 mm, 110 Å) (Torrance, CA, USA). The samples were then separated using a 97 min basic RP-LC gradient as described (Udeshi et al. 2013) and a flow rate of 0.4 mL/min was used. The separated samples were collected into 10 fractions and vacuum-dried prior to LC–MS/MS analysis by a TripleTOF 5600 mass spectrometer (AB SCIEX) coupled online to an Eksigent nanoLC Ultra in Information Dependent Mode. Peptides were separated on a C18 column (Acclaim PepMap C18, 250 mm × 75 μm × 5 μm, 100 Å, Dionex) with a 90 min nonlinear gradient of buffer B (100% ACN, 0.1% FA) from 3 to 30%. The gradient was set as 3–8% B for 10 min, 8–20% B for 60 min, 20–30% B for 8 min, 30–100% B for 2 min, and 100% B for 10 min at a flow rate of 300 nl/min. MS spectra survey scan was done across mass range of 350 to 1500 m/z and the spectra data were acquired at resolution 30,000 with 250 ms accumulation per spectrum. 25 most intense ions from each MS scan were chosen for fragmentation from each MS spectrum with 2 s minimum accumulation time for each precursor and dynamic exclusion for 18 s. Tandem mass spectra were recorded in high sensitivity mode (resolution > 15,000) with rolling collision energy on and iTRAQ reagent collision energy adjustment on.

Peptide and protein identification and quantification was performed with the ProteinPilot 4.5 software (AB SCIEX) using the Paragon database search algorithm. The Chlamydomonas proteome sequences downloaded from UniProt (dated 2–07-2016) were used for the database searching with manual editing using the information (updated on 11–12-2019) in Uniprot database. The false discovery rate (FDR) analysis was performed using the software PSPEP integrated with the ProteinPilot. Confidence of quantitation for differentially expressed proteins was analyzed with the ProteinPilot Descriptive Statistics Template (PDST) (beta v3.07p, AB SCIEX). Gene ontology (GO) enrichment analysis of proteins was performed using DAVID Bioinformatic Resources 6.8 (https://david.ncifcrf.gov/summary.jsp) with the following parameters: ‘Gene List Enrichment,’ ‘Chlamydomonas reinhardtii species,’ ‘Uniprot Accession.’ Functional annotation and classification of the differentially expressed proteins was mainly based on the search results of ‘Biological processes’ in DAVID analysis followed by manual editing using the updated Chlamydomonas genome data in NCBI (updated on 30–01-2018). Differentially expressed proteins were selected with *p* value < 0.05 and cut-off > 1.2 and < 0.83 as up-and down-regulated, respectively.

### Generation and screening of *hpm91*-derived mutants

Insertion mutant library was constructed by transformation of *hpm91* using the glass bead method with KpnI-linearized plasmid pDble containing the *ble* gene conferring zeocin resistance (Kindle 1990). Transformants growing on TAP plates with 10 μg mL^−1^ zeocin (Solarbio) were isolated. After cultured on TAP-S plates for one week, mutants were screened based on increased Y(II) values relative to *hpm91* using Maxi-Imaging PAM chlorophyll fluorometer (Walz, Germany) as previously mentioned (Zhao et al. 2017) followed by H_2_ measurements by GC analysis (Sun et al. 2013; Chen et al. 2019).

## Results and discussion

### H_2_ production of ***hpm91*** mutant was large-scalable

**Fig. 1:**
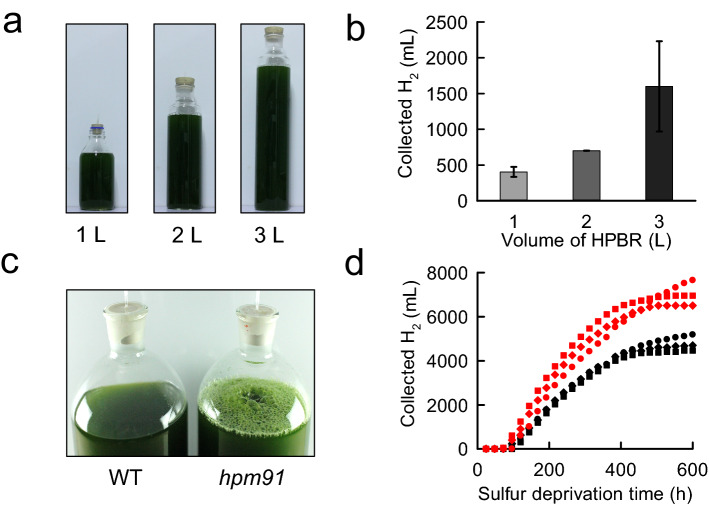
H_2_ production of *hpm91* in 1 to 10 L-HPBR under sulfur deprivation **a** Photograph of 1 to 3 L-HPBR (optical path 10 cm, 100 µmol photons m^−2^ s^−1^.) taken at 10 day of sulfur deprivation. **b** Correlation between H_2_ output of *hpm91* and size of HPBR. Standard deviations were estimated from three biological replicates. **c** Photograph of wild type and *hpm91* in 10L-HPBR taken at 4 day of sulfur deprivation. **d** Comparison of H_2_ output profiles of *hpm91* in 10L-HPBR under light intensity of 130 (in black) and 230 µmol photons m^−2^ s^−1^ (in red). The data are from three independent experiments

Based on experimental results reported so far, *hpm91* appears remarkable among the single mutants deficient in *pgr* genes of Chlamydomonas with enhanced H_2_ photoproduction under sulfur-deprived condition (Tolleter et al. 2011; Steinbeck et al. 2015; Chen et al. 2016; Ho et al. 2022). To determine its potential toward application, we carried out scaling-up and gas-handling experiments in the laboratory using *hpm91* (Fig. 1; Movie S1). Initially, the experiments were performed with H_2_-photobioreactors (HPBR) of scaling-up 10-, 20-, and 30-times (from 100 ml to 1L, 2L, and 3L) with chlorophyll concentration of 25 µg/ml as previously used (Chen et al. 2016; Fig. 1a). Although the yield of H_2_ production in *hpm91* was slightly lower or comparable to the original *pgr5* mutant in 1-L set-ups (Steinbeck et al. 2015; Ho et al. 2022), we found the highest yield of H_2_ from 3L-HPBR and a positive correlation between the yield and size of HPBR (Fig. 1b). These results encouraged us to extend the scaling-up directly to 10L-HPBR (headspace 1700 ml, light path 22 cm, illuminated on 3 sides) and to investigate its H_2_ production profiles. To reduce potential shading effects (Kosourov et al. 2002; Hemschemeier et al. 2009) and low-light stress on algal cells, we reasonably reduced initial chlorophyll concentration (ICC) to 20 µg/ml of cell suspension and cultured under increased light irradiance (130 µmol photons m^−2^ s^−1^) for H_2_ production.

Figure 1c compares H_2_ photoproduction of *hpm91* and wild type in 10L-HPBR under identical cultural condition. In contrast to wild type, which generated few gas bubbles with collectable H_2_ quantity negligible, we observed a bulk of H_2_ bubbles in *hpm91* after 3–4 days of sulfur deprivation (Fig. 1c) with the gas collectable for up to 33 days (Table S1). To confirm these results, experiments were repeated using different batch of algal cells. The data shows that continuous H_2_ collection from *hpm91* could be achieved at an average of 28 (28 ± 4) days under light irradiance of 130 µmol photons m^−2^ s^−1^ (Table S1), which was the longest duration of H_2_ production under sulfur deprivation reported thus far for Chlamydomonas. These results promoted us to explore more possibilities to enhance H_2_ production of *hpm91* in 10L-HPBR.

Indeed, further increase of light intensity to 230 µmol photons m^−2^ s^−1^ apparently enhanced H_2_-producing capability of *hpm91* (Table S1). Our data shows that while the durations of H_2_ production under both light intensities were roughly comparable, the average yield of H_2_ obtained under the elevated light irradiance (230 µmol photons m^−2^ s^−1^) was 7287 (7287 ± 986) ml/10L-HPBR, which was 50.7% higher than that under lower light condition (Table S1). To obtain more information of H_2_ photoproduction, we compared time course of H_2_ collection in *hpm91* grown under such conditions (Fig. 1d). Considering that in both cases H_2_ output was relatively stable around 25 day of sulfur deprivation, H_2_ evolution profiles of *hpm91* during this period were compared. As shown in Fig. 1d, the general kinetic pattern was largely similar but significantly higher average rate of H_2_ production was found (710 ml d^−1^ *vs* 420 ml d^−1^) for those cultured under increased light. Because no further scaling-up data is available thus far for the original *pgr5* mutant, our present results of *hpm91* obtained in the large scale (2–10 L) are novel among the *pgr5* mutants. Further experiments verified its essentially pure H_2_ output which enabled us to make a H_2_ fuel cell-powered toycar drive using ambient air directly (Movie S1). These results allowed us to address that *hpm91* is a potent H_2_ producer for further genetic engineering toward large-scale H_2_ photoproduction.

### Decreased intracellular ROS in ***hpm91*** during sustained H_2_ production

**Fig. 2:**
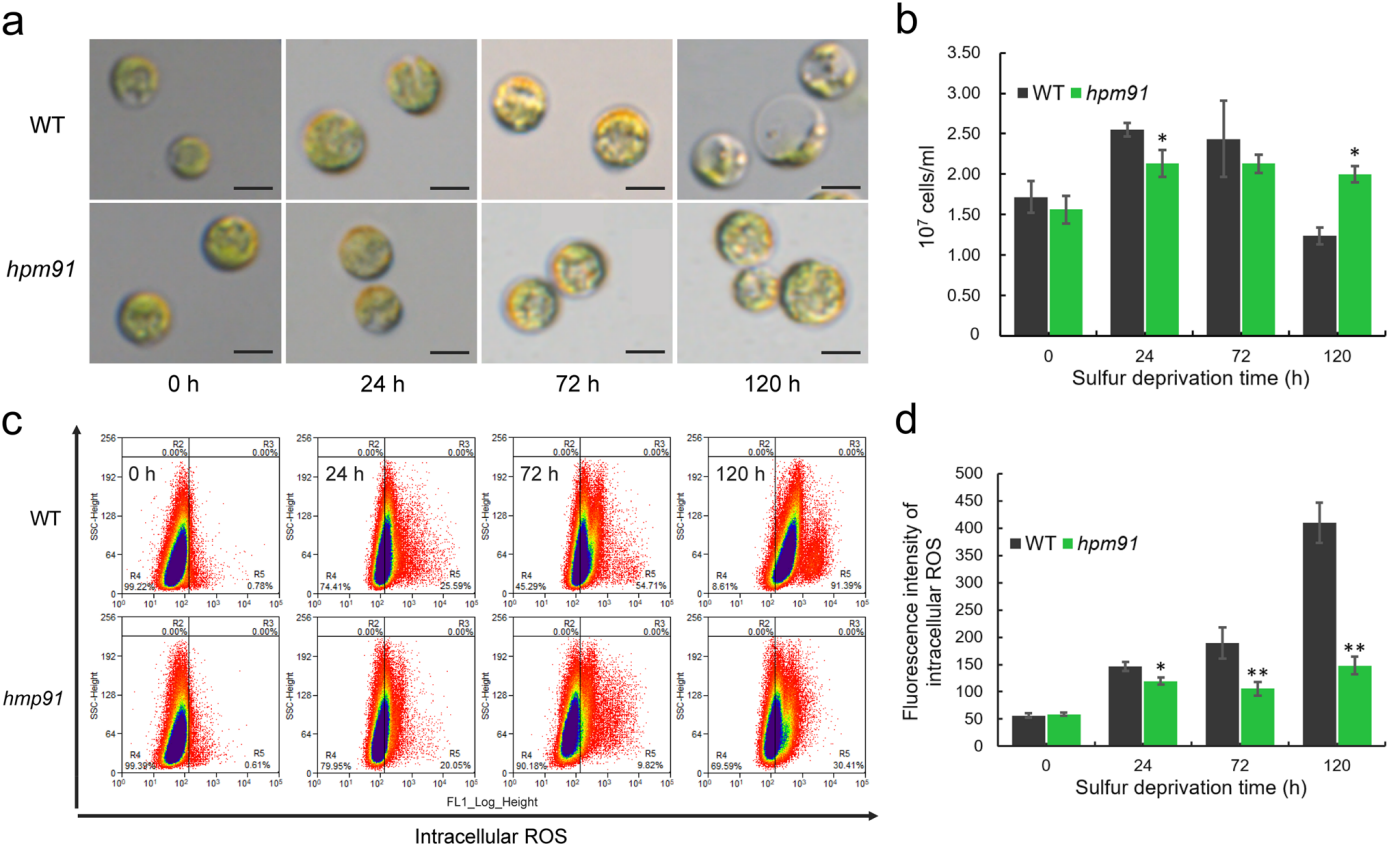
Comparison of intracellular ROS contents of *hpm91* and wild type during 120 h of sulfur-deprived H_2_ production process **a** and **b** Cell morphology and proliferation of the two strains. **c** and **d** Scatter diagram and quantification of intracellular ROS contents in the two strains. Sample preparation was performed under anoxia and ROS levels were determined using a MoFlo XDP high-speed flow cytometer (Beckman-Coulter, Inc. USA) by following the manufacturer’s instructions. Average fluorescence intensity of DCF from 3 × 10^5^ cells was recorded and data acquisition and analysis were carried out using Summit 5.2 software. Experiments were repeated three times with similar results. * and ** refer to *p*-values < 0.05 and < 0.01 in Student’s *t*-test, respectively

Based on visual comparison of cell suspension, cell growth of *hpm91* was always better than wild type in both small (100 ml) and large HPBR (1 to 10 L) especially during prolonged period of H_2_ production. To understand the basis of cell biology, we investigated changes of cell morphology and viability in *hpm91* committed to H_2_ production. Since the small HPBR system was experimentally more efficient, the system was of choice for comparative studies between wild type and *hpm91* during 120 h of H_2_ production (Fig. 2).

Figure 2a shows that wild-type cells became largely translucent, whereas no such changes were observed in *hpm91*. This remarkable morphological alteration of wild-type cells was highly similar to earlier observations which was mainly attributed to the substantial loss of endogenous starch and a declined cell viability under such condition (Zhang et al. 2002). Also, culture density of *hpm91* was more stable after 24 h and remained significantly higher than wild type at the end of the measurements (120 h) (Fig. 2b). Moreover, we found decreased portion of dead cells in the culture of *hpm91* relative to wild type (Fig. S1). These results are clear indications of robustness of *hpm91* cells during sulfur-deprived H_2_ production.

To further elucidate this, we measured intracellular ROS contents of *hpm91* and wild type under such conditions (Fig. 2c and d). ROS levels were determined using a MoFlo XDP high-speed flow cytometer (Beckman-Coulter, Inc. USA) by following the manufacturer’s instructions. Sample preparation was performed under anoxia as described (Chen et al. 2019). Average fluorescence intensity of DCF from 3 × 10^5^ cells was recorded and data acquisition/analysis were carried out using Summit 5.2 software (Beckman-Coulter, Inc. USA) (Fig. 2c). Our data shows that intracellular ROS in *hpm91* remained significantly lower than wild type during H_2_ production. At 120 h, the amount of ROS in *hpm91* was only 1/3 of that in wild type (Fig. 2d). Earlier physiological and biochemical studies strongly implicate occurrence of oxidative stress in Chlamydomonas wild-type and *hpm91*-mutant cells during sulfur-deprived H_2_ photoproduction (Sáenz et al. 2015; Chen et al. 2016; Kosourov et al. 2017). In this work, we demonstrated that cell viability was negatively correlated with intracellular ROS content (Fig. 2; Fig. S1), providing in vivo evidence of toxic effect of excess ROS on cell biology.

### Overview of proteome changes in wild type and *hpm91* under sulfur deprivation

**Fig. 3:**
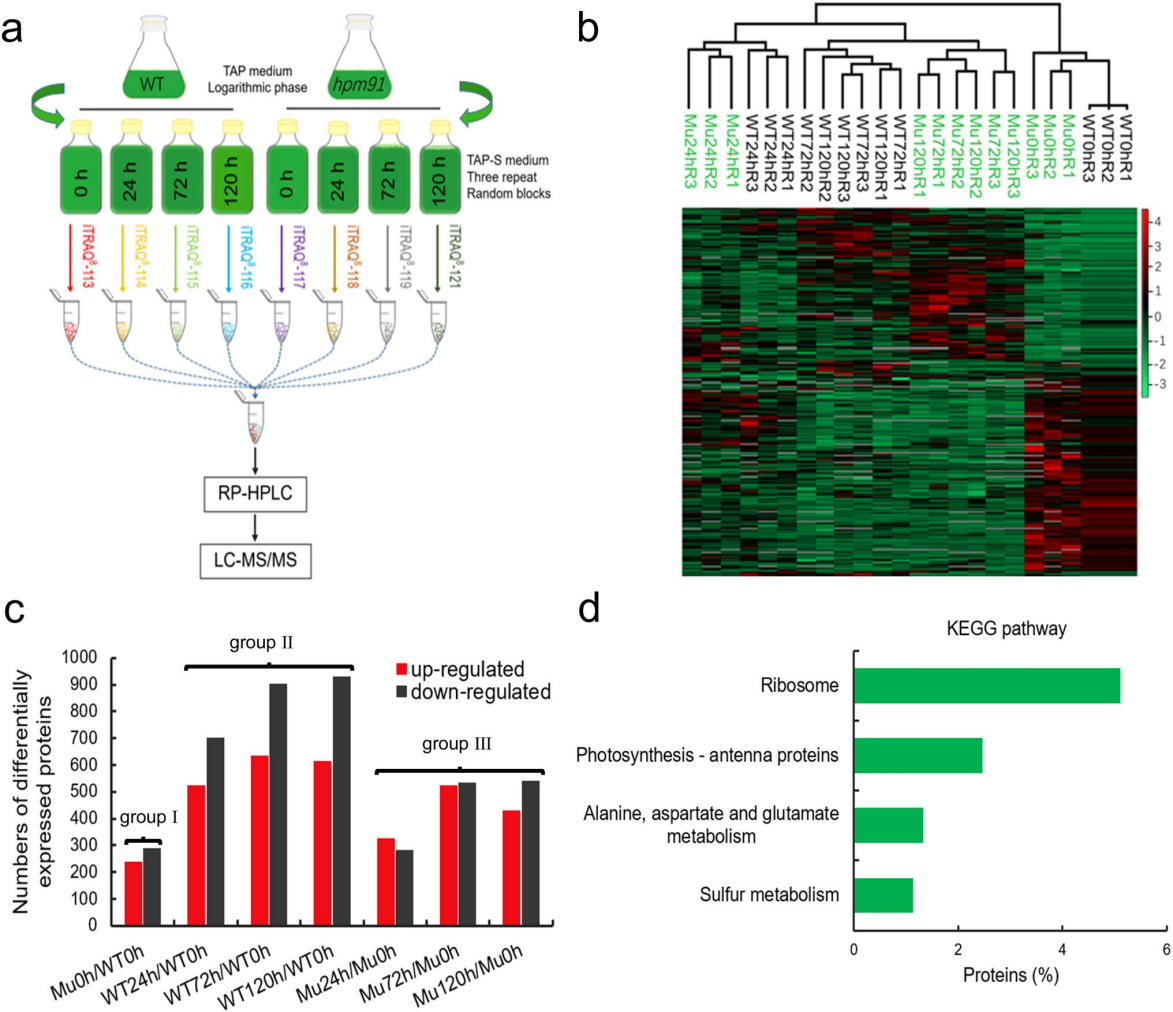
Overview of iTRAQ proteomics of *hpm91* and wild type during 120 h of sulfur-deprived H_2_ production **a** Schematic presentation of iTRAQ experimental design. **b** Hierarchy clustering analysis showing high reproducibility of protein quantitation. **c** Number of differential expressed proteins in *hpm91* and wild type using a cutoff of 1.2-fold change with significance (p < 0.05). **d** KEGG analysis of group I proteins in (c) showing major proteome changes caused by PGR5 deletion in Chlamydomonas. DAVID Bioinformatic Resources 6.8 (https://david.ncifcrf.gov/summary.jsp) was used

To understand the molecular mechanism behind the remarkable phenotype of *hpm91*, we carried out iTRAQ proteomics of *hpm91* and wild-type cells during 120 h of H_2_ production (Fig. 3). As presented in Fig. 3a, samples were taken at four different time points (0, 24, 72 and 120 h) and total proteins were extracted for protein identification and quantification. A total of 3798 proteins with quantitative data were confidently identified (FDR < 1%) as listed in (Dataset 1). Identification was mostly based on minimum of two peptide-hits per protein but considering several proteins such as hydrogenase3 (Hyd3) were probably involved H_2_ metabolism and numerous functionally important small-sized and/or membrane proteins may possess only one identifiable tryptic peptide in MS analysis, those identified with one-peptide hit were also reasonably included (Dataset 1).

Reproducibility of protein quantitation was verified by hierarchical clustering analysis (Perseus _1.6.0.7) of these proteins obtained with three biological replicates (Fig. 3b). To confirm functional impairment of *hpm91* in cyclic electron transfer (CEF), we compared the CEF rates in both strains. The data showed that CEF of *hpm91* was significantly decreased relative to wild type (Fig. S2a). To be more certain with the genetic background of the strains, we performed high-throughput genomic sequencing for wild type (CC400, 137c) and the *pgr5* mutants (*hpm91*, *pgr5*). The data was deposited into CNGB Sequencing Archive1of CNGBdb2 database (accession No. CNP0002674). Further analysis of reads coverage validates previous mutation mapping (Johnson et al. 2014; Chen et al. 2016) and showed large deletions in PGR5-containing region of *hpm91* and *pgr5* mutants (Supplemental Fig. S2b). Together with the immuno-blot results showing no detectable PGR5 but presence of PGRL1 and FNR in *hpm91* (Chen et al. 2016), we clarify that the impaired CEF of *hpm91* is attributed to loss of PGR5. Differentially expressed proteins were determined using a cutoff of 1.2-fold change with significance (*p* < 0.05), leading to three groups consisting of 529 (group I), 2229 (group II), and 1350 (group III) proteins (Fig. 3c) listed in (Datasets 2–4). These correspond to three comparisons, *i.e., hpm91* at 0 h *vs* wild-type at 0 h, any time of- wild type *vs* wild-type at 0 h, and any time of-*hpm91 vs hpm91* at 0 h, representing differentially expressed proteins caused by deletion of PGR5 and by sulfur-deprived anoxia in wild type and *hpm91,* respectively.

**Fig. 4:**
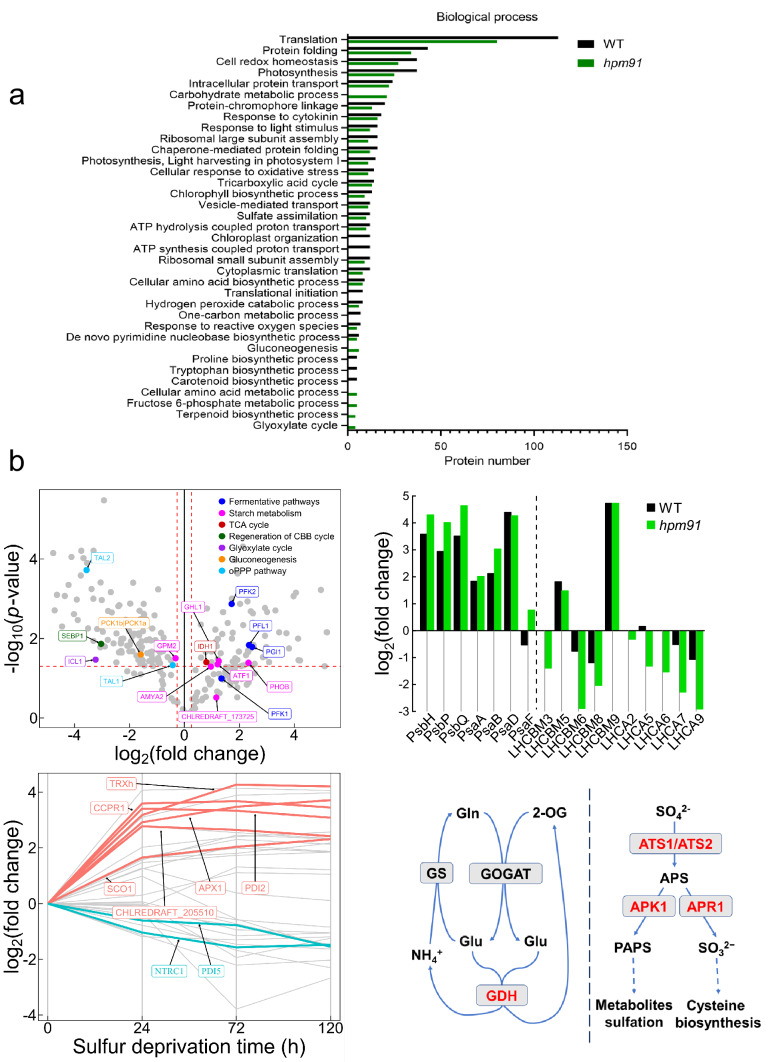
Proteomic characteristics of *hpm91* during sustained H_2_ production **a** Comparison of GOBP enrichments in *hpm91* and wild type during 120 h of sulfur deprivation. **b** Major proteome feature of *hpm91* under sulfur-deprived condition. Volcano plot shows major changes in carbon metabolism of *hpm91* during 120 h of sulfur deprivation (upleft panel). Comparison of average fold-change of photosynthetic proteins in *hpm91* and wild type at 120 h sulfur deprivation (upright panel). Dynamic changes of redox proteins in *hpm91* during 120 of sulfur deprivation (downleft panel). Schematic illustration of N- and S-metabolic features in *hpm91* under sulfur-deprived condition (downright panel)

To understand functional significance of the differentially expressed proteins in each group, KEGG and gene ontology (GO) analysis was performed using DAVID Bioinformatic Resources 6.8 (https://david.ncifcrf.gov/summary.jsp), yielding 4 enriched KEGG pathways for group I (Fig. 3d), 30 and 29 enriched biological process (GOPB) for the later two groups, respectively (Fig. 4a, Datasets 5 and 6). Because numerous proteins in the latter two enrichments were multiply or/and with error assignments, we reasonably delineated them into 8 and 9 major groups as (Wang et al. 2012) and shown in (Tables S2 and S3), respectively.

It can be seen in Table 1, deletion of PGR5 caused significant changes in four pathways in Chlamydomonas. Compared to wild type, all the ribosomal proteins and most of those related to nitrogen metabolism were higher-expressed in *hpm91*. The latter may implicate enhanced nitrogen metabolism in *hpm91*. To test this possibility, we compared phenotype of the two strains under N-starved stress condition. The experimental data showed that both cell growth and photosynthetic capability of *hpm91* was indeed better than wild type (Fig. S2c), revealing another impact of PGR5 on chloroplast biology. Apparently, further investigations are required to uncover the molecular mechanism behind this phenotype. More interestingly, we found all the photosynthetic antenna proteins (except for LHCA3) were lower-expressed in *hpm91*. Because those account for more than 50% of LHCI and LHCII proteins in Chlamydomonas (Shen et al. 2019; Su et al. 2019; Suga et al. 2019), our finding of their reduced levels could be an indication of a smaller photosynthetic antenna in *hpm91* than wild type. Notably, among the proteins involved in sulfur metabolism only APS reductase APR1 encoded by *APR1*/*MET16* (Gutierrez-Marcos et al. 1996; Setya et al. 1996), known to be involved in sulfur-starvation response (Ravina et al. 2002; Zhang et al. 2004) was higher-expressed in *hpm91* (Table 1).

**Table 1:** List of proteins corresponding to Fig. 3d that represents major proteome changes caused by loss of PGR5 in *hpm91*

KEGG pathway	Uniprot accession	Gene ID	Protein name	Ratio (Mu0/Wt0)	*p*-value (< 0.05)
Ribosomal proteins	A8JEP1	PRPL35	50S ribosomal protein L35	1.80	3.73E−02
	A8IQE3	RPL14	Ribosomal protein L14	1.60	2.06E−02
	A8J9D9	PRPL24	Plastid ribosomal protein L24	1.63	2.23E−03
	A8JGK1	RPS17	Ribosomal protein S17	3.41	3.43E−03
	A8HVP2	RPS18	Ribosomal protein S18	3.07	4.63E−02
	A8HQ81	RPL11	Ribosomal protein L11	1.42	3.99E−04
	A8JDN4	PRPS20	Plastid ribosomal protein S20	3.06	7.10E−03
	A8J2G4	RPL32	Ribosomal protein L32	1.53	2.99E−02
	A8HVP7	PRPL10	Plastid ribosomal protein L10	1.51	4.71E−02
	A8IVE2	RPL7	Ribosomal protein L7	2.35	3.00E−02
	A8I0I1	RPS24	40S ribosomal protein S24	1.55	1.83E−02
	A8HVK4	RPS27a	Ribosomal protein S27a	1.47	1.26E−03
	A8IB25	CHLREDRAFT_126059	40S ribosomal protein SA	1.44	1.39E−02
	A8IVK1	RPL8	Ribosomal protein L8	1.97	3.02E−02
	A8INR7	PRPL27	Plastid ribosomal protein L27	2.26	6.07E−03
	A8ID84	RPL3	Ribosomal protein L3	1.59	6.87E−05
	P48267	rps7	Chloroplastic 30S ribosomal protein S7	1.81	3.11E−02
	A8HVQ1	RPS8	40S ribosomal protein S8	1.56	6.57E−03
	A8JF05	RPL28	Ribosomal protein L28	1.43	7.34E−04
	P59776	rps19	Chloroplastic 30S ribosomal protein S19	1.85	3.56E−03
	A8J4Q3	RPS10	Ribosomal protein S10	1.77	1.59E−02
	A8J8P4	RPL34	Ribosomal protein L34	1.39	4.65E−02
	Q9GGE2	rps14	Chloroplastic 30S ribosomal protein S14	1.63	2.27E−02
	A8HMG7	RPL26	Ribosomal protein L26	1.68	4.19E−02
	A8J768	RPS14	Ribosomal protein S14	3.11	2.22E−02
	A8J8M9	RPS20	Ribosomal protein S20	1.55	7.45E−03
	A8HS59	RPL17	Ribosomal protein L17	1.89	2.46E−02
Photosynthesis- antenna proteins	Q75VY6	LHCA6	Light-harvesting protein of photosystem I	0.68	2.93E−02
	Q75VY8	LHCA5	Light-harvesting protein of photosystem I	0.55	1.75E−05
	A8J249	LHCA1	Light-harvesting protein of photosystem I	0.67	4.09E−03
	A8ISG0	LHCA7	Light-harvesting protein of photosystem I	0.64	7.68E−03
	A8ITV3	LHCA9	Light-harvesting protein of photosystem I	0.79	8.00E−03
	A8I0C6	LHCA3	Regulatory chlorophyll a/b binding protein	1.74	3.47E−02
	A8J431	LHCSR3	Stress-related chlorophyll a/b binding protein 2	0.16	1.27E−03
	Q9ZSJ4	LHCBM5	Chlorophyll a-b binding protein of LHCII	0.43	4.64E−03
	Q93WL4	LHCBM3	Light-harvesting chlorophyll a/b binding protein LhcII-1.3	0.53	2.09E−03
	A8JCU4	LHCBM1	Chlorophyll a-b binding protein of LHCII	0.34	1.46E−04
	A8J287	LHCBM6	Chlorophyll a-b binding protein of LHCII type I	0.64	2.04E−02
	A8J270	LHCBM8	Chlorophyll a-b binding protein of LHCII	0.56	4.27E−03
	Q8S3T9	Lhcbm9	Chlorophyll a-b binding protein of LHCII	0.68	4.44E−02
Alanine, aspartate and glutamate metabolism	A8JFZ0	SGA1a|SGA1b	Serine glyoxylate aminotransferase	0.55	1.46E−02
	A8IMN5	CMPS1	Small subunit of carbamoyl phosphate synthase	1.91	9.09E−03
	A8IVZ9	GLN2/GS2	Glutamine synthetase	1.85	2.27E−05
	A8I263	AST3	Aspartate aminotransferase	1.71	5.04E−03
	A8HXW8	AST4	Aspartate aminotransferase	2.11	1.80E−02
	A8JE06	CHLREDRAFT_122298	Predicted protein	1.66	1.44E−02
	A8IW34	PURA	Adenylosuccinate synthetase	2.33	9.86E−04
Sulfur metabolism	A8J3Q6	APK1	Adenylyl-sulfate kinase	0.64	6.72E−03
	A8J6A7	APR1/MET16	Adenylylphosphosulfate reductase	1.40	5.90E−04
	A8I3V3	ATS2	ATP-sulfurylase	0.62	4.34E−03
	Q6QJE1	SABC	Chloroplast ATP-binding protein	0.56	2.43E−02
	A8JDD3	SAT2	Serine O-acetyl transferase	0.24	1.35E−02
	A8IEE5	OASTL3	Cysteine synthase	0.46	1.48E−02

Comparison of the results in Tables S2 and S3 revealed similarities in 5 functional groups, *i.e.,* translation, protein folding, intracelluar protein trafficking, response to cytokinin, and ATP hydrolysis/production in the two strains. However, differences were also revealed in 4 of those corresponding to carbon metabolism, photosynthetic antenna, cell redox homeostasis/anti-oxidative systems, and nitrogen- and sulfur metabolisms. These are the major proteomic characteristics of *hpm91*-cells committed to sulfur-deprived H_2_ production, which are described/discussed in the following section.

### Proteomic characteristics of ***hpm91*** during sustained H_2_ production

#### Loss of PGR5 in *hpm91* causes compromised primary carbon metabolism

As can be seen in Fig. 4a, six biological processes were only enriched in *hpm91, i.e.,* ‘carbohydrate metabolism,’ ‘gluconeogenesis,’ ‘fructose 6-phosphate metabolic process,’ ‘glyoxylate cycle,’ ‘cellular amino acid metabolic process,’ and ‘terpenoid biosynthetic process,’ with the first one appeared within top-10 rankings. This implicates that loss of PGR5 caused more profound alterations of primary carbon metabolism in *hpm91* than wild type during H_2_ production. Considering metabolic relevance in Chlamydomonas under anoxia (Yang et al. 2015), the group ‘tricarboxylic acid cycle’ was combined with the first four groups in (Table S3). While many of them overlapped with wild type, 16 proteins were exclusively revealed in *hpm91* during sustained H_2_ production. These include the key proteins in the regeneration pathway of CBB cycle (SEBP1), glyoxylate cycle (ICL1), gluconeogenesis (PCK1b |PCK1a), oxidative PPP pathway (TAL1, TAL2), fermentative pathways (PFK1, PFK2, PGI1, PFL1), starch metabolism (AMYA2, AMY-like protein, GHL1, ATF1, PHOB, GPM2), and TCA cycle (IDH1), showing decreased and increased trends for the most proteins in the first four and the latter three pathways, respectively (Fig. 4b, upleft pannel). It was earlier reported that loss of PFL1 decreased H_2_ photoproduction (Philipps et al. 2011). Our finding of increased amount of PFL1 in *hpm91* was in line with this and may suggest a partial contribution of PFL1 to its enhanced H_2_ production. Also, we found two alpha-amylases, glucosamine-fructose-6-phosphate aminotransferase (ATF1) and phosphorylase PHOB increased at an average of 1.86 to 5.02-fold in *hpm91* during H_2_ production (Table S3). These enzymes are known to be essential for starch metabolism (Weigelt et al. 2009). While accumulation of PHOB could be correlated to the marked increase of starch contents in *hpm91* (Chen et al. 2016), elevated level of the amylases was somehow intriguing because starch breakdown in *hpm91* was found significantly less than wild type (Chen et al. 2016), excluding the major contribution of ‘indirect pathway’ on the prolonged H_2_ production phenotype of *hpm91*. Moreover, our data revealed down-regulated key enzymes in or related to CBB cycle (PRK1, FBP1, SEBP1) in *hpm91*, suggesting that the main route of CO_2_ fixation was largely repressed toward H_2_ production. It is possible that the increased levels of amylases as well as GHL1 and ATF1 in *hpm91* is to yield various intermediates satisfying increased carbon demand under anoxic conditions (Weigelt et al. 2009). Yet, a large variation of the key enzymes in TCA cycle of *hpm91*, such as malate dehydrogenase MDH4 and subunits of succinate dehydrogenase (SDH1, SDH2, SDH4) was observed (Table S3). This may reflect dynamic energetic status in *hpm91* during sulfur-deprived H_2_ production.

#### *hpm91* is characteristic of increased photosynthetic core and decreased PSI antenna

In Table S3, nearly 50% of the PSII and PSI proteins were accumulated in *hpm91* especially PsbH, PsbP, PsbQ, PsaA, PsaB, PsaD, and PsaF with average values of fold-increase within 3.1 to 25.2. Because H_2_ evolution profile of wild type and *hpm91* was mostly distinct at 120 h of sulfur deprivation (Chen et al. 2019), the change-fold of this time point was compared between the two strains (Fig. 4b, upright pannel). Compared to wild type, fold-increase values of the three PSII proteins was about 2-times larger in *hpm91*. Considering that PsbP and PsbQ are essential in maintaining water-splitting reaction (Shen 2015) and PsbH is crucial for stable assembly and optimal function of PSII (Umena et al. 2011; Trosch et al. 2018), their greater increase in *hpm91* may partially explain its significantly higher residual PSII activity during prolonged H_2_ production (Chen et al. 2019). Regarding to LHCII proteins, it was noted that decrease of LHCBM3, LHCBM6, LHCBM8 were more pronounced in *hpm91* under such conditions (Fig. 4b, upright panel). Most interestingly, we found that while the fold-increase values of PSI core subunits of *hpm91* were either higher (PsaB and PsaF) or comparable (PsaA, PsaD) to wild type, all the LHCA proteins displayed declined trends with greater values of decrease-fold in *hpm91* than wild type during H_2_ production process (Fig. 4b, upright panel). These results strongly suggest that *PGR5*-deficient *hpm91* mutant is an algal strain characteristic of small-sized PSI antenna under not only normal condition (Table 1) but also during sustained sulfur-deprived H_2_ production. It is generally known that, in wild type, transcriptional regulation of LHC genes plays a central role in antenna size adjustment. To test if this is true for *hpm91* mutant, we then carried out qRT-PCR analysis of the genes encoding the LHCA proteins. Our data showed that their mRNA levels were indeed down-regulated during H_2_ production (Fig. S3). Taken together, we suggest that mutation of *PGR5* caused not only the impaired CEF (Fig. S2a) but also significantly reduced PSI antenna (Fig. 4b), leading to its higher efficiency of light utilization than wild type toward H_2_ photoproduction (Kosourov et al. 2011).

Strikingly, we found the level of LHCA2 protein in *hpm91* was remarkably decreased during H_2_ production, whereas no change was revealed in wild type (Fig. 4b, upright panel). This distinction could be of strong indication of LHCA2 as a negative effector on H_2_ photoproduction in Chlamydomonas. Indeed, a recent report by Ho et al. (Ho et al. 2022) shows that *pgr5/lhca2* double mutant produced more than twofold H_2_ amount relative to its single *pgr5* mutant, revealing the crucial role of LHCA2 involved in algal H_2_ photoproduction, strongly suggesting that *lhca2* is a potent gene target for further genetic modifications of the organism toward H_2_ photoproduction.

#### Reinforced cell redox homeostasis and anti-oxidative systems in *hpm91*

Based on comparison of Tables S2 and S3, a higher percentage of up-regulated proteins involved in cell redox homeostasis and/or anti-oxidative stress responses was revealed in *hpm91* than wild type under such condition. Although most of them overlapped with wild type, accumulation of several proteins in TRX superfamily, mitochondrial proteins SCO1 and cytochrome c peroxidase CCPR1 as well as those related to oxidative stress responses was more pronounced in *hpm91* than wild type. Strikingly, the amount of TRXh, PDI2, APX1, and CCPR1 increased at least tenfold in *hpm91* during prolonged H_2_ production (Fig. 4b, downleft panel). Based on physiological and biochemical analysis of wild-type cells, it has been earlier suggested that oxidative stress occurs during sulfur-deprived H_2_ photoproduction (Sáenz et al. 2015; Kosourov et al. 2017). Regarding the *pgr5* mutants (*pgr5, hpm91*), we have previously observed both increased ROS tolerance and ROS-scavenging enzyme activity under such conditions (Chen et al. 2016). In this work, we found the higher percentage and abundance of those proteins involved in cell redox homeostasis and/or anti-oxidative stress reactions (Tables S2 and S3; Fig. 4b, downleft panel). Together with the finding of better cell viability of *hpm91* than wild type during sulfur-deprived H_2_ photoproduction (Fig. 2), we suggest that the lower amount of ROS observed in *hpm91* (Fig. 2c and d) could be largely attributed to the marked increase of both protein abundance of those and activity of the ROS-scavenging enzymes as well as putatively reduced PSI antenna mentioned above (Lu et al. 2021). A question is open how this is fulfilled in the mutant cells. Considering that H_2_ production is beneficial for cell survival and maintenance of photosynthetic apparatus activity as well as energy and redox status under such conditions (Chen et al., 2016; 2019; Antal et al. 2020), we presume that shifting to the H_2_ production mode is one of the best choices for the mutant cells acclimating to sulfur-deprived anaerobic condition.

#### Enhanced N- and S- metabolism in ***hpm91*** during sustained H_2_ photoproduction

Comparison of Tables S2 and S3 reveals that both N- and S- metabolism of *hpm91* was also enhanced during sustained H_2_ photoproduction. Considering that glutamine and glutamate are the major intracellular amino group donors for the synthesis of several other amino acids and nitrogen-containing compounds including purine and pyrimidine nucleobases (Zhang et al. 2018), we combined the functional groups of ‘amino acid pathways,’ ‘terpenoid- and de novo biosynthesis of pyrimidine nucleobase’ (Fig. 4a) and referred as N-metabolism in Table S3. In contrast to wild type, 3 proteins of those were only accumulated in *hpm91* at 120 h of sulfur deprivation. These were aspartate aminotransferase AST3, glutamate dehydrogenase GDH and GDH2, showing upto 2.20-, 13.42-, and 24.56-fold increase (Table S3). AST3 is known to be one of the major enzymes catalyzing conversion of glutamate and oxaloacetic acid (OAA) into asparate and 2-oxoglutarate (2-OG/alpha-KG), an intermediate of the TCA cycle (Ohashi et al. 2011) that serves as the metabolic basis for coupling N- and C-metabolisms in photosynthetic organisms. Because AST3 was already higher-expressed in *hpm91* under normal condition (Table 1), the continued increase is strongly indicating its dominant role in asparate biosynthesis and/or maintenance of C/N metabolic balance in the *PGR5*-deficient *hpm91* mutant during prolonged H_2_ production.

More interestingly, 2 glutamate dehydrogenases (GDH and GDH2) were markedly accumulated in *hpm91* during sustained H_2_ production. These proteins are supposed to play an anaplerotic role in ammonium assimilation via conversion of Glu into 2-OG and ammonium in Chlamydomonas (Moyano et al. 1995). Their remarkable increase in *hpm91* implicates activation of this minor pathway of ammonium assimilation in the mutant under such condition. Together with upregulation of NADH-dependent glutamate synthase GSN1 (Table S3), the key enzyme of the major route GS-GOGAT cycle, we propose that, due to loss of PGR5, both the major and minor route of ammonium assimilation was activated/or enhanced in *hpm91* during sustained H_2_ photoproduction. Because enhanced ammonium assimilation requires higher demand of the carbon skeleton 2-OG (Ohashi et al. 2011) for coupling between nitrogen and carbon metabolism (Zhang et al. 2018), we propose that by stimulating the anaplerotic role of the GDH toward producing more 2-OG, carbon, and nitrogen metabolism could be better coupled in *hpm91* than wild type under such conditions.

In this work, we also found remarkable accumulations for 5 key proteins involved in sulfur assimilation in *hpm91* during H_2_ photoproduction (Table S3). These include not only the above-mentioned APR1 (Table 1) with continued increase in abundance (Table S3) but also ATP-sulfurylases (ATS1, ATS2) as well as cysteine synthase OASTL3 toward incorporation SO_4_^2+^ into cysteine (Gonzales-Ballester et al. 2009). Because the latter two proteins were found lower-expressed in *hpm91* under normal condition (Table 1), their marked accumulation and that of ATS1 during prolonged H_2_ production may strongly indicate activation of the pathway toward cysteine biosynthesis (Fig. 4b, downright pannel). Meanwhile, we observed upto 4.5-fold accumulation of APK1 in *hpm91* during H_2_ production process (Table S3), suggesting sulfur-assimilation pathway toward cysteine biosynthesis sulfation of metabolites cysteine biosynthesis (Gonzales-Ballester et al. 2009) was also enhanced in *hpm91* (Fig. 4b, downright panel). These results strongly indicate that overall sulfur metabolism was more enhanced in *hpm91* relative to wild type during sulfur-deprived H_2_ production process.

### Creating mutants with H_2_ production excess to ***hpm91***

**Fig. 5:**
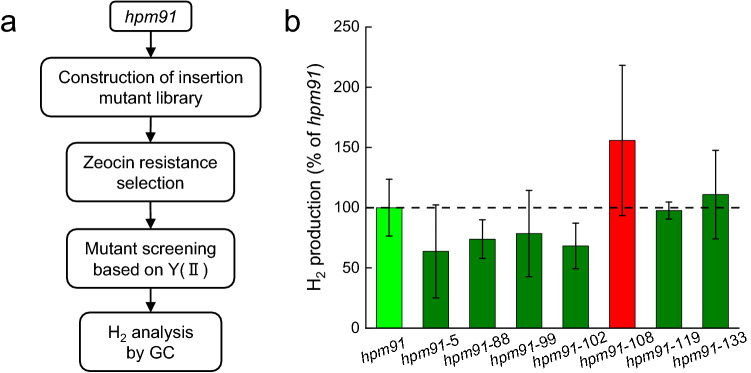
Screening for H_2_ production mutants excess to *hpm91* **a** Outline of the screening method. **b** Phenotype confirmation of the *hpm91*-derived mutants. H_2_-producing capability of the selected mutants was determined with a GC-2014 gas chromatographer (Shimadzu; Japan) at 5 days of sulfur deprivation. Standard deviations were estimated from 3 biological replicates

Since the data described above suggests that *hpm91* is better suited for sulfur-deprived H_2_ production photosynthetically, metabolically, and redox poised, we hypothesized that the strain can be used as a “chassis cell” for creating new mutant strains with enhanced H_2_ production relative to *hpm91*. To test this, an insertion mutant library derived from *hpm91* was constructed according to (Kindle 1990; Zhao et al. 2017) followed by tranformants selection via zeocin resistance (Fig. 5a). Considering that increased stability of PSII is essential to sustain sufur-deprived H_2_ photoproduction in Chlamydomonas (Volgusheva et al. 2013; Chen et al. 2019), a subsequent ‘two-step mutant screening’ was applied, *i. e.* Y(II) measurements using Maxi-Imaging PAM chlorophyll fluorometer (Walz, Germany) as the first followed by H_2_-generating phenotype confirmation by GC analysis as described (Sun et al. 2013). Preliminary mutant screening identified over two hundred transformants with 10% increase of Y(II) values than *hpm91* under sulfur deprivation. Subsequent screening by GC analysis identified one of the mutants, named *hpm91-108,* with significantly higher H_2_ production than *hpm91* (Fig. 5b). At 120 h, H_2_ production in *hpm91-108* was 55. 9% higher than *hpm91*. This result is the direct experimental evidence of *hpm91* as a potent strain for re-engineering the organism toward advancing photobiological H_2_ production.

## Concluding remarks

In summary, scaling-up and in-depth analysis of *hpm91* mutant has revealed several valuable properties toward development of sunlight-powered algal H_2_ production systems in the near future. First, it is largely up-scalable using the ‘two-step’ protocol of H_2_ induction by sulfur deprivation (Melis et al. 2000). In both steps, up to 100-fold extension of PBR (10 L, mixotrophic growth) (Chen et al. 2019) and HPBR (10 L, H_2_ photoproduction) was achieved in the laboratory set-ups, leading to an average H_2_ output of 7287 ml/10L-HPBR for averagely 26 days (this work). Second, *hpm91* is robust during prolonged H_2_ production. In the absence of PGR5, *hpm91* shows competent viability than wild type and remains active over a long period of sulfur deprivation, which could be mainly due to a decrease of intracellular ROS involved in signaling pathways (Mullineaus et al. 2008). Third, *hpm91* was active metabolically (reinforced anti-ROS systems, compromised carbon metabolism, enhanced/activation of anaplerotic route of ammonium assimilation and sulfur assimilation) and photosynthetically (optimal structure and function of PSII and PSI, reduced size of PSI antenna and CEF) toward sustained H_2_ photoproduction. These results reveal not only new insights of cellular and molecular basis of enhanced H_2_ production in *hpm91* but also provide additional candidate gene targets and modules for further genetic modifications and/or in artificial photosynthesis mimics (Ye et al. 2021) toward basic and applied research aiming at advancing solar-H_2_ technology.

## Supplementary Information

Below is the link to the electronic supplementary material.

Supplementary file1 Fig. S1 Comparison of percentage of dead cells in the culture of hpm91 and wild type during 120 h of sulfur-deprived H2 production. Fig. S2 Functional and genomic verification of PGR5 deletion in hpm91. Fig. S3 qRT-PCR analysis of relative gene expression of the LHCAs in hpm91 during 120 h of sulfur-deprived H2 production (DOCX 1962 kb)

Supplementary file2 Table S1 Comparison of H2 output of hpm91 in 10L-HPBR under different light intensities. Table S2 List of differentially expressed proteins in wild type delineated from Dataset 5 that represents major proteome changes during H2 production process. Table S3 List of differentially expressed proteins in hpm91 delineated from Dataset 6 that represents major proteome changes during H2 production process. Table S4 List of primers used in this work (DOCX 219 kb)

Supplementary file3 Demonstration of a laboratory set-up making a H2-fuel-cell-powered toycar drive by input of algal-H2 collected from hpm91 and ambient air. (AVI 2917 kb)

Supplementary file4 Dataset 1 List of confidentially identified proteins with quantitative information. (XLSX 2292 kb)

Supplementary file5 Dataset 2 List of proteins corresponding to group I of Fig. 3C (XLSX 64 kb)

Supplementary file6 Dataset 3 List of proteins corresponding to group II of Fig. 3C (XLSX 518 kb)

Supplementary file7 Dataset 4 List of proteins corresponding to group III of Fig. 3C (XLSX 336 kb)

Supplementary file8 Dataset 5 GOBP enrichment results derived from Dataset 3 (XLSX 140 kb)

Supplementary file9 Dataset 6 GOBP enrichment results derived from Dataset 4 (XLSX 112 kb)
