# Supplementary material for: Scaling-up and proteomic analysis reveals photosynthetic and metabolic insights toward prolonged H2 photoproduction in Chlamydomonas hpm91 mutant lacking proton gradient regulation 5 (PGR5)
Source: Photosynth Res. 2022 Aug 16;154(3):397–411. doi: 10.1007/s11120-022-00945-4 (PMC9722884; doi:10.1007/s11120-022-00945-4)
Supplement: Supplementary file 1 — Supplementary file1 Fig. S1 Comparison of percentage of dead cells in the culture of hpm91 and wild type during 120 h of sulfur-deprived H2 production. Fig. S2 Functional and genomic verification of PGR5 deletion in hpm91. Fig. S3 qRT-PCR analysis of relative gene expression of the LHCAs in hpm91 during 120 h of sulfur-deprived H2 production (DOCX 1962 kb) [file 11120_2022_945_MOESM1_ESM.docx]

**Supporting Information for manuscript by Liu et al. submitted to Photosynthesis Research:**

**Title:** Scaling-up and proteomic analysis reveals photosynthetic and metabolic insights towards prolonged H_2_ photoproduction in Chlamydomonas *hpm91* mutant lacking Proton Gradient Regulation 5 (PGR5)

**Authors:** Peng Liu^a,e,1^, De-Min Ye^a,e,1^, Mei Chen ^a,1^, Jin Zhang^a,e,1^ , Xia-He Huang^b^, Li-Li Shen^a,e^, Ke-Ke Xia^c^, Xiao-Jing Xu^c,e^, Yong-Chao Xu^d,e^, Ya-Long Guo^d^, Ying-Chun Wang^b,^*, and Fang Huang ^a,^*

**Affiliations:** ^a^Photosynthesis Research Center, Key Laboratory of Photobiology, Institute of Botany, Chinese Academy of Sciences, Beijing 100093, China; ^b^State Key Laboratory of Molecular Developmental Biology, Institute of Genetics and Developmental Biology, Chinese Academy of Sciences, Beijing 100101, China; ^c^BGI-Shenzhen, Shenzhen 518083, China; ^d^State Key Laboratory of Systematic and Evolutionary Botany, Institute of Botany, Chinese Academy of Sciences, Beijing 100093, China; ^e^University of Chinese Academy of Sciences, Beijing 100049, China

***Corresponding authors:**

Fang Huang: e-mail, [fhuang@ibcas.ac.cn](mailto:fhuang@ibcas.ac.cn); phone, +86-10-62836692; fax, +86-10-62594363

Ying-Chun Wang: e-mail, ycwang@genetics.ac.cn; phone, +86-10-64806149

**SI files include:**

Figures S1 to S3

Tables S1 to S4

Movie S1

Datasets 1 to 6

**Supporting information Fig. S1**


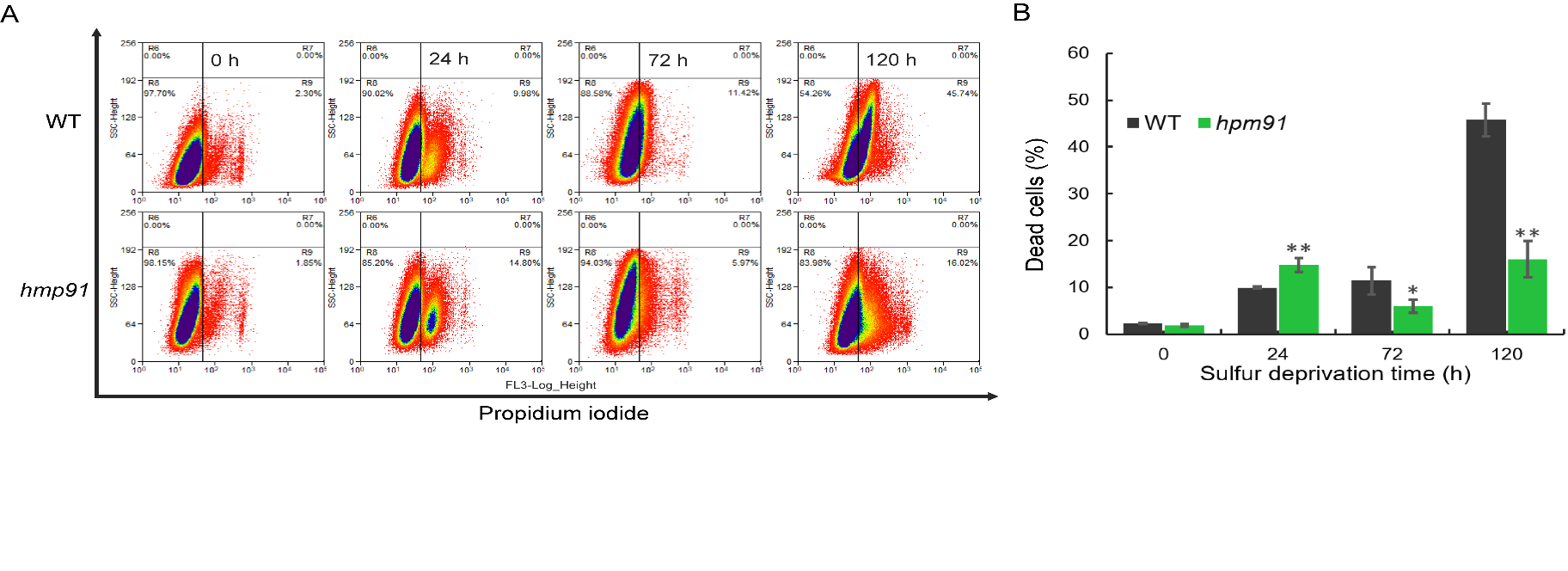


a

b

**Supporting information Fig. S1** Comparison of percentage of dead cells in the culture of *hpm91* and wild type during 120 h of sulfur-deprived H_2_ production (a) Cells were incubated with propidium iodide (PI; 10 μg·mL^−1^) in an anoxia workstation (Longyao, LAI-3T; Shanghai, China). Fluorescence of PI inside the cells was detected with excitation at 488 nm and emission 510 to 550 nm. Data acquisition and analysis was carried out using Summit 5.2 software (Beckman Coulter, Inc. USA). (b) The percentage of membrane damaged cells (dead cells) was a proportion of the total population. Experiments were repeated three times with similar results. * and ** refer to *p*-values <0.05 and <0.01 in Student’s t-test, respectively.

**Supporting information Fig. S2**


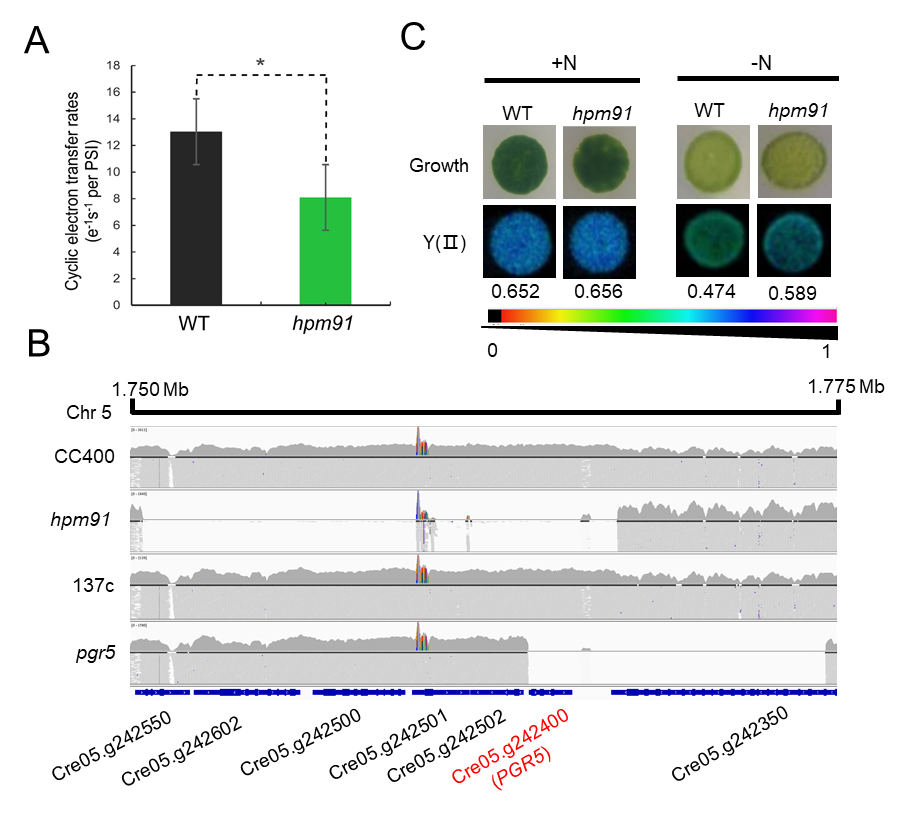


a

c

b

Supporting information Fig. S2 Functional and genomic verification of *PGR5* deletion in *hpm91*. (a) Comparison of CEF in wild type and *hpm91* Measurements were performed with a JTS-10 spectrometer (BioLogic, France) using samples prepared according to (Takahashi et al. 2013; Alric 2010). Standard deviations were estimated from 3 biological replicates. Experiments were repeated three times with similar results. *refers to p-values ＜0.05 in Student’s t-test. (b) Genomic coverage of four strains of *hpm91*, CC400, 137c and *pgr5* (these two strains were also purchased from Chlamydomonas Center, www.Chlamy.org). Genomic DNA was isolated using the Plant Genomic DNA Kit (TiangenBiotech; Beijing, China) as described in (Chen et al. 2016). High-throughput sequencing was carried out in DNBSEQ-T1 platform (BGI-Shenzhen, China) with clean data obtained via SOAPnuke software (Chen et al. 2018) followed by mapping to Chlamydomonas reference genome (Merchant et al. 2007) using BWA-mem algorithms (Li 2013) and deposited at the CNGBdb database (Guo et al. 2020; Chen et al. 2020)(https://db.cngb.org/search; accession No. CNP0002674). Further analysis of genomic region containing *PGR5* was done with IGV software (Thorvaldsdottir et al. 2013). The thick blue lines in the bottom indicates genes on the chromosome 5: 1.750-1.775 Mb. Each grey line indicates the reads coverage of the four strains, which reveals two large deletions in the strains of *hpm91* and *pgr5* both with the gene loss of *PGR5*. (c) Loss of PGR5 led to enhanced tolerance to N-starvation. Mid-exponential phase cells with density of 2 x 10^6^ cells mL^-1^ were spotted on TAP plates (+N, -N) and grown for 5 days. Y(II) was measured with as Maxi-Imaging PAM (Walz, Germany) as described in (Zhao et al. 2017). Experiments were repeated three times with similar results.

**Supporting information Fig. S3**


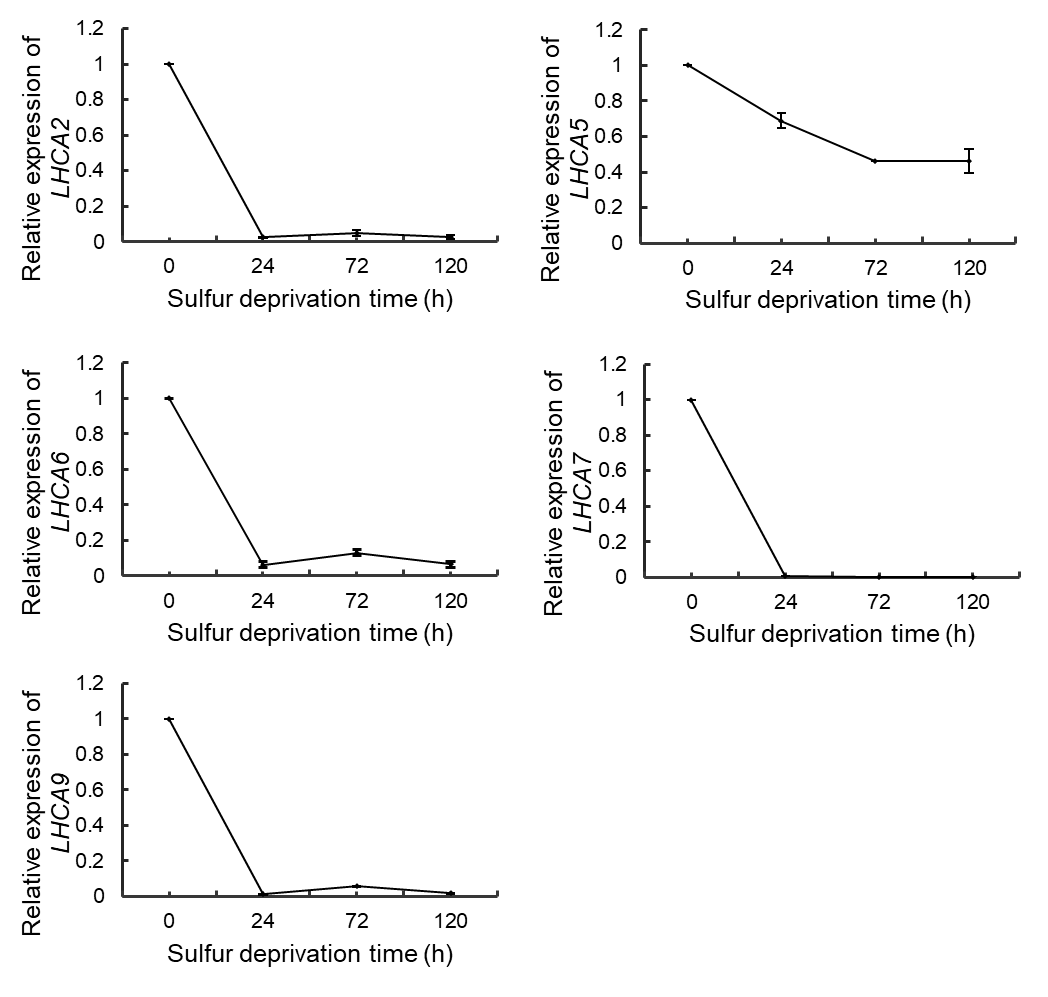


**Supporting information Fig. S3** qRT-PCR analysis of relative gene expression of the LHCAs in *hpm91* during 120 h of sulfur-deprived H_2_ production. Measurements were done according to (Zhao et al. 2017). Standard deviations were estimated from 3 biological replicates each with 3 technical replicates (n = 9). Similar results were obtained in at least three independent experiments. The CBLP gene was used as a control.

**Supporting information References**

Alric J (2010) Cyclic electron flow around photosystem I in unicellular green algae. Photosynth Res 106: 47-56. <https://doi.org/10.1007/s11120-010-9566-4>.

Chen FZ, You LJ, Yang F, Wang LN, Guo XQ, Gao F, Hua C, Tan C et al (2020) CNGBdb: China National GeneBank DataBase. Hereditas(Beijing) 42: 799-809. <https://doi.org/10.16288/j.yczz.20-080>.

Chen M, Zhang J, Zhao L, Xing JL, Peng LW, Kuang TY, Rochaix JD, Huang F (2016) Loss of algal proton gradient regulation 5 increases reactive oxygen species scavenging and H_2_ evolution. Journal of Integrative Plant Biology 58: 943-946. <https://doi.org/10.1111/jipb.12502>.

Chen YX, Chen YS, Shi CM, Huang ZB, Zhang Y, Li SK, Li Y, Ye J et al. (2018). SOAPnuke: a MapReduce acceleration-supported software for integrated quality control and preprocessing of high-throughput sequencing data. Gigascience 7: 1-6. <https://doi.org/10.1093/gigascience/gix120>.

Guo XQ, Chen FZ, Gao F, Li L, Liu K, You LJ, Hua C, Yang F et al (2020) CNSA: a data repository for archiving omics data. Database-Oxford. ARTN baaa055 <https://doi.org/10.1093/database/baaa055>.

Li H (2013) Aligning sequence reads, clone sequences and assembly contigs with BWA-MEM. arXiv:1303.3997. <https://arxiv.org/abs/1303.3997>.

Merchant SS, Prochnik SE, Vallon O, Harris EH, Karpowicz SJ, Witman GB, Terry A, Salamov A et al (2007). The Chlamydomonas genome reveals the evolution of key animal and plant functions. Science 318: 245-251. <https://doi.org/10.1126/science.1143609>.

Takahashi H, Clowez S, Wollman FA, Vallon O Rappaport F (2013) Cyclic electron flow is redox-controlled but independent of state transition. Nat Commun 4: 1954 <https://doi.org/10.1038/ncomms2954>.

Thorvaldsdottir H, Robinson JT Mesirov JP (2013) Integrative Genomics Viewer (IGV): high-performance genomics data visualization and exploration. Brief Bioinform 14: 178-192. <https://doi.org/10.1093/bib/bbs017>.

Zhao L, Cheng DM, Huang XH, Chen M, Dall'Osto L, Xing JL, Gao LY, Li LY et al (2017) A light harvesting complex- like protein in maintenance of photosynthetic components in Chlamydomonas. Plant Physiol 174: 2419-2433. <https://doi.org/10.1104/pp.16.01465>.
