# Supplementary material for: Scaling-up and proteomic analysis reveals photosynthetic and metabolic insights toward prolonged H2 photoproduction in Chlamydomonas hpm91 mutant lacking proton gradient regulation 5 (PGR5)
Source: Photosynth Res. 2022 Aug 16;154(3):397–411. doi: 10.1007/s11120-022-00945-4 (PMC9722884; doi:10.1007/s11120-022-00945-4)
Supplement: Supplementary file 2 — Supplementary file2 Table S1 Comparison of H2 output of hpm91 in 10L-HPBR under different light intensities. Table S2 List of differentially expressed proteins in wild type delineated from Dataset 5 that represents major proteome changes during H2 production process. Table S3 List of differentially expressed proteins in hpm91 delineated from Dataset 6 that represents major proteome changes during H2 production process. Table S4 List of primers used in this work (DOCX 219 kb) [file 11120_2022_945_MOESM2_ESM.docx]

**Supporting Information for manuscript by Liu et al. submitted to Photosynthesis Research:**

**Title:** Scaling-up and proteomic analysis reveals photosynthetic and metabolic insights towards prolonged H_2_ photoproduction in Chlamydomonas *hpm91* mutant lacking Proton Gradient Regulation 5 (PGR5)

**Authors:** Peng Liu^a,e,1^, De-Min Ye^a,e,1^, Mei Chen ^a,1^, Jin Zhang^a,e,1^ , Xia-He Huang^b^, Li-Li Shen^a,e^, Ke-Ke Xia^c^, Xiao-Jing Xu^c,e^, Yong-Chao Xu^d,e^, Ya-Long Guo^d^, Ying-Chun Wang^b,^*, and Fang Huang ^a,^*

**Affiliations:** ^a^Photosynthesis Research Center, Key Laboratory of Photobiology, Institute of Botany, Chinese Academy of Sciences, Beijing 100093, China; ^b^State Key Laboratory of Molecular Developmental Biology, Institute of Genetics and Developmental Biology, Chinese Academy of Sciences, Beijing 100101, China; ^c^BGI-Shenzhen, Shenzhen 518083, China; ^d^State Key Laboratory of Systematic and Evolutionary Botany, Institute of Botany, Chinese Academy of Sciences, Beijing 100093, China; ^e^University of Chinese Academy of Sciences, Beijing 100049, China

***Corresponding authors:**

Fang Huang: e-mail, [fhuang@ibcas.ac.cn](mailto:fhuang@ibcas.ac.cn); phone, +86-10-62836692; fax, +86-10-62594363

Ying-Chun Wang: e-mail, ycwang@genetics.ac.cn; phone, +86-10-64806149

**SI files include:**

Figures S1 to S3

Tables S1 to S4

Movie S1

Datasets 1 to 6

**Supporting Information Table S1.** Comparison of H_2_ output of *hpm91* in 10L-HPBR under different light intensities.

| No. | | Light irradiance  (µE m^−2^ s^−1^) | | Chlorophyll  (µg/ml) | SD | Duration time (d) | SD | Collected H_2_ (ml) | SD |
| --- | --- | --- | --- | --- | --- | --- | --- | --- | --- |
| Experiment | 1 | | 130 | 20.0 |  | 33 |  | 5315 |  |
|  | 2 | | 130 | 18.4 |  | 25 |  | 4465 |  |
|  | 3 | | 130 | 19.1 |  | 27 |  | 4718 |  |
| Average |  | |  | 19.2 | 0.8 | 28 | 4 | 4832 | 436 |
|  |  | |  |  |  |  |  |  |  |
| Experiment | 1 | | 230 | 18.3 |  | 33 |  | 8395 |  |
|  | 2 | | 230 | 18.3 |  | 21 |  | 6505 |  |
|  | 3 | | 230 | 18.1 |  | 23 |  | 6960 |  |
| Average |  | |  | 18.2 | 0.1 | 26 | 6 | 7287 | 986 |

**Supporting Information Table S2.** List of differentially expressed proteins in wild type delineated from Dataset S5 that represents major proteome changes during H_2_ production process.

| Ranking | GOPB | Uniprot accession | Gene ID | Protein name | Ratio (WTt/WT0) | | |  | *p*-value | | |
| --- | --- | --- | --- | --- | --- | --- | --- | --- | --- | --- | --- |
|  |  |  |  |  | 24 h | 72 h | 120 h |  | 24 h | 72 h | 120 h |
| 1 | Translation | A8J9T0 | RPS12 | 40S ribosomal protein S12 | 2.18 | 1.34 | 0.40 |  | 2.50E-01 | 2.97E-01 | 2.45E-02 |
|  |  | A8IXG3 | RPS21 | 40S ribosomal protein S21 | 1.10 | 0.63 | 0.72 |  | 7.51E-01 | 2.43E-02 | 4.78E-01 |
|  |  | A8I0I1 | RPS24 | 40S ribosomal protein S24 | 1.63 | 0.66 | 0.30 |  | 3.45E-01 | 4.00E-02 | 4.31E-02 |
|  |  | A8J576 | RPS27-A | 40S ribosomal protein S27 | 0.43 | 0.14 | 0.07 |  | 1.51E-03 | 1.08E-07 | 8.81E-06 |
|  |  | A8J0V6 | RPS27-B | 40S ribosomal protein S27 | 2.96 | 2.96 | 2.08 |  | 1.51E-02 | 7.29E-03 | 2.67E-02 |
|  |  | A8HS48 | CHLREDRAFT_168484 | 40S ribosomal protein S3a | 1.33 | 0.39 | 0.10 |  | 6.15E-01 | 9.77E-05 | 1.07E-05 |
|  |  | A8HVQ1 | RPS8 | 40S ribosomal protein S8 | 0.32 | 0.16 | 0.08 |  | 4.41E-04 | 1.60E-06 | 5.04E-06 |
|  |  | A8IB25 | CHLREDRAFT_126059 | 40S ribosomal protein SA | 1.13 | 0.58 | 0.22 |  | 5.50E-01 | 3.48E-05 | 2.53E-03 |
|  |  | A8IUV7 | RPL13 | 60S ribosomal protein L13 | 1.72 | 0.34 | 0.09 |  | 2.16E-01 | 1.76E-07 | 1.31E-06 |
|  |  | A8IKZ2 | RPL18 | 60S ribosomal protein L18 | 1.21 | 0.35 | 0.09 |  | 4.94E-01 | 5.94E-05 | 8.35E-06 |
|  |  | A8IQC1 | RPL27 | 60S ribosomal protein L27 | 14.85 | 6.63 | 3.01 |  | 4.76E-04 | 2.12E-02 | 5.73E-02 |
|  |  | Q8GUQ9 | RPL38 | 60S ribosomal protein L38 | 4.17 | 0.91 | 0.63 |  | 3.16E-02 | 7.11E-01 | 2.99E-01 |
|  |  | A8J1B6 | ANT1 | Adenine nucleotide translocator | 0.49 | 0.48 | 0.36 |  | 3.79E-02 | 1.79E-02 | 5.40E-04 |
|  |  | Q84X74 | MPC1b\|MPC1a | CR057 protein | 1.42 | 4.27 | 2.72 |  | 6.31E-02 | 2.67E-02 | 2.53E-02 |
|  |  | A8JHX9 | EFG2 | Elongation factor 2 | 1.77 | 1.14 | 0.20 |  | 1.19E-01 | 6.39E-01 | 1.70E-03 |
|  |  | A8JCA8 | EFG5 | Elongation factor EF-Tu-like protein | 1.15 | 2.23 | 2.48 |  | 2.36E-01 | 6.07E-03 | 7.58E-03 |
|  |  | A8IAC7 | EIF5Ba | Eukaryotic initiation factor | 0.51 | 0.83 | 0.64 |  | 2.69E-03 | 3.04E-01 | 5.59E-02 |
|  |  | A8HX38 | EEF1 | Eukaryotic translation elongation factor 1 alpha 1 | 1.13 | 0.67 | 0.10 |  | 3.74E-01 | 2.16E-01 | 2.37E-06 |
|  |  | A8J8J8 | CHLREDRAFT_120661 | GTPase Der | 0.44 | 0.43 | 0.44 |  | 1.68E-03 | 5.38E-04 | 5.25E-02 |
|  |  | A8JCK3 | GBA1 | GTP binding protein TypA | 0.48 | 0.63 | 1.11 |  | 3.77E-04 | 2.70E-01 | 8.43E-01 |
|  |  | A8IFH4 | LPA1 | Membrane GTP-binding protein LepA | 0.39 | 0.51 | 0.44 |  | 2.40E-02 | 4.70E-02 | 1.66E-02 |
|  |  | A8JB67 | NHP2 | Nucleolar protein small subunit of H/ACA snoRNPs | 0.70 | 0.37 | 0.15 |  | 1.23E-01 | 1.15E-05 | 3.91E-05 |
|  |  | A8J9X5 | CHLREDRAFT_18599 | Predicted protein | 0.86 | 0.54 | 0.74 |  | 5.62E-01 | 7.32E-02 | 1.98E-03 |
|  |  | A8I9M5 | CHLREDRAFT_141578 | Predicted protein | 0.14 | 0.14 | 0.16 |  | 7.34E-08 | 5.99E-07 | 5.02E-04 |
|  |  | A8JHJ5 | CHLRE_15g641200v5 | Predicted protein | 0.08 | 0.15 | 0.12 |  | 2.52E-07 | 7.48E-06 | 3.48E-06 |
|  |  | A8I2G3 | CHLREDRAFT_112251 | Predicted protein | 0.57 | 0.48 | 0.51 |  | 3.46E-03 | 2.43E-03 | 5.58E-02 |
|  |  | A8HXD0 | CHLREDRAFT_134139 | Predicted protein | 0.96 | 0.70 | 0.72 |  | 3.27E-01 | 7.35E-03 | 1.42E-01 |
|  |  | A8HZV5 | CHLREDRAFT_187641 | Predicted protein | 0.50 | 0.46 | 0.47 |  | 3.73E-02 | 5.22E-02 | 1.87E-03 |
|  |  | A8I4T2 | RPL10a | Ribosomal protein | 21.35 | 14.90 | 11.18 |  | 1.64E-02 | 1.74E-02 | 3.30E-02 |
|  |  | A8IZK3 | RPL10 | Ribosomal protein L10 | 1.19 | 0.30 | 0.12 |  | 5.62E-01 | 3.35E-05 | 2.26E-04 |
|  |  | A8HQ81 | RPL11 | Ribosomal protein L11 | 5.40 | 1.87 | 1.11 |  | 5.51E-04 | 3.92E-04 | 7.82E-01 |
|  |  | A8J597 | RPL12 | Ribosomal protein L12 | 1.75 | 0.47 | 0.25 |  | 2.96E-01 | 4.09E-03 | 5.09E-03 |
|  |  | A8HS59 | RPL17 | Ribosomal protein L17 | 3.66 | 0.97 | 0.46 |  | 3.89E-02 | 8.94E-01 | 1.28E-01 |
|  |  | A8IA18 | RPL19 | Ribosomal protein L19 | 0.50 | 0.11 | 0.05 |  | 6.68E-03 | 1.80E-05 | 1.84E-09 |
|  |  | A8J951 | RPL21 | Ribosomal protein L21 | 3.97 | 1.37 | 0.41 |  | 4.16E-02 | 4.04E-01 | 1.38E-01 |
|  |  | A8J239 | RPL23a | Ribosomal protein L23a | 1.10 | 0.29 | 0.13 |  | 7.90E-01 | 9.73E-05 | 2.66E-05 |
|  |  | A8J1A3 | RPL24 | Ribosomal protein L24 | 1.02 | 0.44 | 0.14 |  | 9.46E-01 | 2.84E-05 | 1.12E-06 |
|  |  | A8I2T0 | RPL27a | Ribosomal protein L27a | 0.45 | 0.11 | 0.04 |  | 1.81E-03 | 3.91E-07 | 2.59E-09 |
|  |  | A8ID84 | RPL3 | Ribosomal protein L3 | 0.40 | 0.18 | 0.04 |  | 2.42E-03 | 4.92E-08 | 5.94E-09 |
|  |  | A8ICT1 | RPL30 | Ribosomal protein L30 | 2.73 | 0.80 | 0.38 |  | 2.29E-01 | 2.64E-01 | 2.76E-02 |
|  |  | A8ILG8 | RPL31 | Ribosomal protein L31 | 0.98 | 0.22 | 0.11 |  | 9.63E-01 | 4.25E-04 | 3.86E-05 |
|  |  | A8J2G4 | RPL32 | Ribosomal protein L32 | 1.13 | 0.31 | 0.14 |  | 7.85E-01 | 6.98E-04 | 2.82E-04 |
|  |  | A8J8P4 | RPL34 | Ribosomal protein L34 | 0.38 | 0.23 | 0.12 |  | 3.76E-04 | 2.98E-07 | 8.59E-05 |
|  |  | A8HNX3 | RPL35 | Ribosomal protein L35 | 8.38 | 2.40 | 1.59 |  | 7.12E-03 | 1.92E-02 | 4.00E-01 |
|  |  | A8HY08 | RPL37a | Ribosomal protein L37a | 0.71 | 0.37 | 0.18 |  | 1.26E-01 | 2.10E-03 | 1.43E-05 |
|  |  | A8J0I0 | RPL4 | Ribosomal protein L4 | 0.49 | 0.18 | 0.06 |  | 1.20E-02 | 2.49E-06 | 2.43E-07 |
|  |  | A8HP55 | RPL5 | Ribosomal protein L5 | 2.95 | 1.47 | 0.69 |  | 2.45E-02 | 3.91E-02 | 3.78E-01 |
|  |  | A8HP90 | RPL6 | Ribosomal protein L6 | 2.22 | 0.67 | 0.39 |  | 1.37E-01 | 2.76E-02 | 2.04E-02 |
|  |  | A8J567 | RPL7a | Ribosomal protein L7a | 6.85 | 2.95 | 1.22 |  | 6.39E-02 | 4.38E-02 | 7.86E-01 |
|  |  | A8JHC3 | RPS11 | Ribosomal protein S11 | 0.27 | 0.12 | 0.04 |  | 1.50E-05 | 2.75E-07 | 2.03E-07 |
|  |  | A8IGY1 | RPS13 | Ribosomal protein S13 | 1.48 | 0.36 | 0.09 |  | 4.44E-01 | 3.24E-05 | 4.92E-10 |
|  |  | A8J493 | RPS15 | Ribosomal protein S15 | 2.67 | 0.92 | 0.34 |  | 8.80E-02 | 7.74E-01 | 8.07E-03 |
|  |  | A8JE07 | RPS15a | Ribosomal protein S15a | 2.34 | 0.85 | 0.30 |  | 2.48E-02 | 5.27E-01 | 3.07E-03 |
|  |  | A8JGK1 | RPS17 | Ribosomal protein S17 | 27.56 | 11.70 | 5.88 |  | 2.49E-02 | 1.30E-02 | 3.15E-01 |
|  |  | A8HVP2 | RPS18 | Ribosomal protein S18 | 20.38 | 9.99 | 5.76 |  | 2.46E-02 | 1.87E-02 | 1.48E-01 |
|  |  | A8I403 | RPS19 | Ribosomal protein S19 | 11.02 | 4.78 | 2.51 |  | 3.40E-03 | 5.98E-03 | 2.79E-01 |
|  |  | A8HME4 | RPS2 | Ribosomal protein S2 | 0.98 | 0.32 | 0.10 |  | 8.87E-01 | 8.20E-07 | 1.72E-05 |
|  |  | A8J8M9 | RPS20 | Ribosomal protein S20 | 1.26 | 0.39 | 0.16 |  | 4.47E-01 | 1.46E-09 | 4.01E-04 |
|  |  | A8IS22 | RPS26 | Ribosomal protein S26 | 0.67 | 0.18 | 0.09 |  | 2.23E-01 | 2.60E-04 | 6.17E-06 |
|  |  | A8HVK4 | RPS27a | Ribosomal protein S27a | 0.22 | 0.07 | 0.03 |  | 2.28E-03 | 2.65E-07 | 1.02E-07 |
|  |  | A8IKP1 | RPS28 | Ribosomal protein S28 | 9.86 | 7.71 | 4.05 |  | 1.14E-01 | 2.08E-01 | 4.77E-02 |
|  |  | A8I4P5 | RPS3 | Ribosomal protein S3 | 1.62 | 0.42 | 0.18 |  | 2.89E-01 | 3.64E-04 | 4.32E-04 |
|  |  | A8JF66 | RPS30 | Ribosomal protein S30 | 0.75 | 0.27 | 0.12 |  | 1.63E-01 | 3.19E-04 | 6.60E-05 |
|  |  | A8IMP6 | RPS4 | Ribosomal protein S4 | 1.53 | 0.29 | 0.06 |  | 4.73E-01 | 2.58E-06 | 4.68E-08 |
|  |  | A8J2I5 | RPS5 | Ribosomal protein S5 | 16.37 | 10.13 | 6.52 |  | 1.79E-02 | 2.01E-02 | 1.56E-01 |
|  |  | A8HYC0 | UTP1 | Nucleolar protein component of the U3 processome | 1.13 | 0.70 | 0.86 |  | 8.20E-01 | 1.77E-02 | 6.70E-01 |
|  |  | A8JHU2 | RPL36 | 60S ribosomal protein L36 | 6.91 | 2.24 | 0.85 |  | 4.49E-02 | 2.38E-01 | 7.66E-01 |
|  |  | A8J5Z0 | RPP0 | Acidic ribosomal protein P0 | 1.08 | 0.36 | 0.09 |  | 7.59E-01 | 1.37E-03 | 5.04E-07 |
|  |  | A8J0R4 | RPP2 | Acidic ribosomal protein P2 | 3.56 | 3.63 | 3.14 |  | 2.96E-02 | 3.15E-02 | 1.08E-01 |
|  |  | A8IML7 | EIF3X | Hypothetical translation initiation factor (Fragment) | 3.17 | 1.70 | 0.87 |  | 4.27E-02 | 1.87E-01 | 6.67E-01 |
|  |  | A8I982 | RPL15 | Ribosomal protein L15 | 1.15 | 0.34 | 0.07 |  | 6.81E-01 | 1.49E-04 | 8.61E-07 |
|  |  | A8JI94 | RPL22 | Ribosomal protein L22 | 21.08 | 11.10 | 6.18 |  | 1.75E-03 | 5.70E-03 | 3.78E-02 |
|  |  | A8HMG7 | RPL26 | Ribosomal protein L26 | 2.04 | 0.42 | 0.14 |  | 6.81E-02 | 6.18E-03 | 8.88E-06 |
|  |  | A8I0Y2 | RPL35a | Ribosomal protein L35a | 1.10 | 0.29 | 0.08 |  | 6.18E-01 | 1.69E-06 | 2.89E-07 |
|  |  | A8IVE2 | RPL7 | Ribosomal protein L7 | 10.68 | 5.29 | 2.56 |  | 4.76E-04 | 2.87E-04 | 1.39E-01 |
|  |  | A8IVK1 | RPL8 | Ribosomal protein L8 | 0.49 | 0.20 | 0.13 |  | 4.86E-02 | 3.26E-05 | 8.57E-05 |
|  |  | A8ILL5 | EIF2Ab | Eukaryotic initiation factor (Fragment) | 5.54 | 3.11 | 2.30 |  | 2.26E-02 | 9.66E-04 | 1.91E-01 |
|  |  | A8JHM2 | EIF3C | Eukaryotic initiation factor (Fragment) | 0.57 | 0.33 | 0.20 |  | 6.46E-02 | 1.10E-04 | 2.20E-04 |
|  |  | A8IP17 | CHLREDRAFT_188942 | Eukaryotic initiation factor 4A-like protein | 0.60 | 0.54 | 0.15 |  | 1.64E-02 | 4.55E-05 | 4.34E-05 |
|  |  | A8J9A9 | EIF3F | Eukaryotic initiation factor | 2.07 | 1.38 | 1.26 |  | 2.32E-02 | 2.60E-01 | 4.69E-01 |
|  |  | A8J4L1 | CHLREDRAFT_119356 | Predicted protein (Fragment) | 0.64 | 0.58 | 0.37 |  | 3.57E-02 | 3.74E-02 | 5.58E-03 |
|  |  | A8J2I8 | CHLREDRAFT_104111 | Predicted protein (Fragment) | 0.85 | 0.74 | 0.44 |  | 1.17E-01 | 2.99E-01 | 3.67E-02 |
|  |  | A8IBY2 | CHLREDRAFT_111269 | Predicted protein (Fragment) | 0.86 | 0.43 | 0.34 |  | 4.01E-01 | 4.51E-03 | 1.86E-03 |
|  |  | A8HTK2 | CPLD10 | Predicted protein | 0.78 | 0.66 | 0.67 |  | 4.35E-03 | 1.07E-02 | 7.41E-02 |
|  |  | Q7YKX3 | rps11 | 30S ribosomal protein S11 chloroplastic | 0.41 | 0.29 | 0.26 |  | 9.93E-04 | 2.08E-04 | 3.06E-03 |
|  |  | Q9GGE2 | rps14 | 30S ribosomal protein S14 chloroplastic | 2.17 | 1.07 | 1.02 |  | 4.56E-06 | 7.42E-01 | 5.00E-01 |
|  |  | O20032 | rps18 | 30S ribosomal protein S18 chloroplastic | 2.90 | 0.34 | 0.27 |  | 3.01E-02 | 1.50E-02 | 4.69E-03 |
|  |  | P59776 | rps19 | 30S ribosomal protein S19 chloroplastic | 0.38 | 0.11 | 0.17 |  | 5.15E-02 | 5.17E-06 | 9.81E-04 |
|  |  | O47027 | rps2-1 | 30S ribosomal protein S2 chloroplastic | 0.25 | 0.16 | 0.12 |  | 3.46E-04 | 3.76E-06 | 7.48E-05 |
|  |  | Q08365 | rps3 | 30S ribosomal protein S3 chloroplastic | 0.33 | 0.12 | 0.13 |  | 8.56E-04 | 3.88E-06 | 9.15E-05 |
|  |  | P48270 | rps4 | 30S ribosomal protein S4 chloroplastic | 0.96 | 0.29 | 0.14 |  | 6.97E-01 | 3.80E-04 | 3.30E-06 |
|  |  | P48267 | rps7 | 30S ribosomal protein S7 chloroplastic | 4.71 | 1.80 | 1.70 |  | 3.25E-03 | 2.77E-02 | 1.03E-01 |
|  |  | P59775 | rps8 | 30S ribosomal protein S8 chloroplastic | 1.21 | 0.59 | 0.52 |  | 6.18E-01 | 1.93E-02 | 1.60E-01 |
|  |  | O20029 | rps9 | 30S ribosomal protein S9 chloroplastic | 0.31 | 0.17 | 0.11 |  | 7.67E-04 | 7.22E-06 | 1.59E-05 |
|  |  | P11094 | rpl14 | 50S ribosomal protein L14 chloroplastic | 0.68 | 0.12 | 0.20 |  | 3.22E-02 | 1.41E-08 | 1.75E-03 |
|  |  | Q8HTL2 | rpl2 | 50S ribosomal protein L2 chloroplastic | 0.19 | 0.05 | 0.05 |  | 2.04E-04 | 3.55E-07 | 1.83E-07 |
|  |  | P26565 | rpl20 | 50S ribosomal protein L20 chloroplastic | 1.82 | 0.27 | 0.15 |  | 2.06E-01 | 8.73E-04 | 5.41E-06 |
|  |  | Q8HTL3 | rpl23 | 50S ribosomal protein L23 chloroplastic | 0.76 | 0.12 | 0.13 |  | 9.08E-02 | 7.08E-06 | 1.07E-04 |
|  |  | A8J3Z3 | PRPL31 | 50S ribosomal protein L31 | 0.34 | 0.08 | 0.15 |  | 1.30E-05 | 1.49E-07 | 1.26E-04 |
|  |  | A8JEP1 | PRPL35 | 50S ribosomal protein L35 | 1.33 | 0.49 | 0.22 |  | 4.85E-01 | 4.83E-02 | 9.30E-05 |
|  |  | P59774 | rpl36 | 50S ribosomal protein L36 chloroplastic | 0.20 | 0.11 | 0.06 |  | 2.14E-03 | 1.78E-05 | 2.13E-05 |
|  |  | Q8HTL1 | rpl5 | 50S ribosomal protein L5 chloroplastic | 2.79 | 0.68 | 0.37 |  | 1.09E-02 | 6.66E-02 | 1.61E-03 |
|  |  | A8I8Z4 | PRPL1 | Plastid ribosomal protein L1 | 0.40 | 0.06 | 0.04 |  | 8.41E-04 | 8.88E-07 | 7.65E-10 |
|  |  | A8ICE4 | PRPL11 | Plastid ribosomal protein L11 | 1.22 | 0.19 | 0.21 |  | 4.61E-01 | 2.52E-05 | 1.48E-05 |
|  |  | A8HWZ6 | PRPL13 | Plastid ribosomal protein L13 | 0.32 | 0.08 | 0.08 |  | 3.65E-04 | 1.59E-08 | 4.22E-07 |
|  |  | A8JAL6 | PRPL15 | Plastid ribosomal protein L15 | 0.24 | 0.04 | 0.05 |  | 3.39E-04 | 2.86E-07 | 2.65E-07 |
|  |  | A8I3M4 | PRPL17 | Plastid ribosomal protein L17 | 1.34 | 0.27 | 0.59 |  | 2.95E-02 | 3.70E-04 | 2.55E-02 |
|  |  | A8HNJ8 | PRPL18 | Plastid ribosomal protein L18 | 0.49 | 0.08 | 0.07 |  | 2.13E-02 | 2.02E-09 | 9.00E-08 |
|  |  | A8IW44 | PRPL19 | Plastid ribosomal protein L19 | 0.48 | 0.10 | 0.15 |  | 9.39E-03 | 1.18E-07 | 1.67E-04 |
|  |  | A8IP00 | PRPL21 | Plastid ribosomal protein L21 | 0.54 | 0.19 | 0.37 |  | 3.92E-02 | 5.71E-03 | 5.38E-02 |
|  |  | A8J9D9 | PRPL24 | Plastid ribosomal protein L24 | 1.49 | 0.23 | 0.24 |  | 4.87E-01 | 2.09E-05 | 2.94E-04 |
|  |  | A8INR7 | PRPL27 | Plastid ribosomal protein L27 | 0.26 | 0.14 | 0.15 |  | 8.50E-04 | 8.24E-05 | 9.15E-05 |
|  |  | A8HWS8 | PRPL28 | Plastid ribosomal protein L28 | 0.21 | 0.05 | 0.06 |  | 5.06E-04 | 3.61E-07 | 1.30E-06 |
|  |  | A8JE35 | PRPL3 | Plastid ribosomal protein L3 | 0.23 | 0.04 | 0.03 |  | 2.72E-04 | 4.87E-08 | 4.45E-10 |
|  |  | A8IUC3 | PRPL32 | Plastid ribosomal protein L32 | 2.41 | 0.35 | 0.33 |  | 1.33E-01 | 5.31E-03 | 4.08E-03 |
|  |  | A8I1D3 | PRPL33 | Plastid ribosomal protein L33 | 1.03 | 0.41 | 0.43 |  | 7.78E-01 | 5.97E-05 | 4.32E-03 |
|  |  | Q84U22 | PRPL4 | Plastid ribosomal protein L4 | 2.76 | 0.59 | 0.40 |  | 2.10E-01 | 1.84E-02 | 7.93E-02 |
|  |  | A8J503 | PRPL6 | Plastid ribosomal protein L6 | 0.94 | 0.16 | 0.15 |  | 8.44E-01 | 3.37E-05 | 6.67E-05 |
|  |  | A8HTY0 | PRPL7/L12 | Plastid ribosomal protein L7/L12 | 3.16 | 1.76 | 1.34 |  | 1.35E-02 | 7.12E-03 | 2.51E-01 |
|  |  | A8IYS1 | PRPL9 | Plastid ribosomal protein L9 | 0.79 | 0.12 | 0.07 |  | 5.29E-01 | 1.65E-06 | 7.78E-10 |
|  |  | A8JGS2 | PRPS17 | Plastid ribosomal protein S17 | 0.45 | 0.08 | 0.09 |  | 3.05E-02 | 2.19E-10 | 3.44E-06 |
|  |  | A8JDN4 | PRPS20 | Plastid ribosomal protein S20 | 6.41 | 2.20 | 3.12 |  | 3.86E-04 | 8.18E-02 | 1.28E-02 |
|  |  | A8J8M5 | PRPS5 | Plastid ribosomal protein S5 | 0.18 | 0.05 | 0.07 |  | 1.71E-05 | 9.97E-09 | 2.59E-07 |
|  |  | A8IMN3 | PSRP-6 | Plastid-specific ribosomal protein 6 | 0.11 | 0.05 | 0.05 |  | 3.79E-05 | 1.07E-07 | 6.60E-08 |
|  |  | Q8HUH1 | rps2-2 | Putative 30S ribosomal S2-like protein | 1.87 | 1.15 | 1.08 |  | 1.02E-07 | 4.75E-01 | 7.72E-01 |
|  |  | A8IA39 | EFG1 | Chloroplast elongation factor G | 0.36 | 0.45 | 0.15 |  | 1.35E-03 | 6.65E-03 | 9.62E-05 |
|  |  | A8HTK7 | TBA1 | PsbA translation factor | 1.05 | 0.73 | 0.51 |  | 7.33E-01 | 9.95E-02 | 5.55E-03 |
|  |  | A8IPJ0 | MITC11 | Mitochondrial carrier protein | 0.50 | 0.67 | 0.47 |  | 3.18E-03 | 8.80E-02 | 1.49E-04 |
|  |  | A8IXI7 | MITC10 | Mitochondrial carrier protein | 0.72 | 0.93 | 0.76 |  | 2.70E-02 | 3.64E-01 | 3.24E-02 |
|  |  | A8HXM1 | MRPL29 | Mitochondrial ribosomal protein L29 | 9.54 | 2.56 | 2.79 |  | 3.83E-02 | 2.01E-01 | 2.66E-01 |
|  |  | A8HPX6 | MRPL3 | Mitochondrial ribosomal protein L3 | 0.86 | 0.67 | 0.54 |  | 1.18E-01 | 3.64E-04 | 7.34E-05 |
|  |  | A8JFK9 | MRPL7/L12 | Mitochondrial ribosomal protein L7/L12 | 2.68 | 2.68 | 2.28 |  | 0.00E+00 | 1.84E-04 | 1.99E-03 |
|  |  | A8JCF4 | MRPS17 | Mitochondrial ribosomal protein S17 | 0.70 | 0.44 | 0.50 |  | 3.46E-02 | 1.94E-03 | 6.40E-02 |
|  |  | A8JGX6 | MRPS6 | Mitochondrial ribosomal protein S6 | 0.76 | 0.75 | 0.87 |  | 1.39E-01 | 4.84E-02 | 6.05E-01 |
|  |  | A8J1X0 | UCP1 | Uncoupling protein | 0.62 | 0.63 | 0.51 |  | 3.78E-04 | 1.19E-02 | 7.34E-03 |
|  |  | A8HXY9 | UCP2 | Uncoupling protein | 2.05 | 2.44 | 2.27 |  | 1.36E-01 | 1.39E-02 | 1.61E-02 |
|  |  | A8J3F7 | MITC14 | Mitochondrial substrate carrier protein | 0.67 | 0.34 | 0.27 |  | 2.05E-01 | 3.24E-03 | 2.61E-03 |
|  |  | A8J2A5 | MSCP1 | Mitochondrial substrate carrier protein | 0.60 | 0.35 | 0.52 |  | 3.02E-02 | 4.42E-04 | 3.19E-02 |
| 2 | Photosynthesis | P10898 | psbC | Photosystem II CP43 chlorophyll apoprotein | 0.31 | 2.01 | 1.38 |  | 2.50E-03 | 1.29E-01 | 5.10E-01 |
|  |  | P06007 | psbD | Photosystem II D2 protein | 0.12 | 1.14 | 1.02 |  | 2.97E-07 | 2.99E-01 | 9.28E-01 |
|  |  | P22666 | psbH | Photosystem II reaction center protein H | 12.59 | 15.21 | 12.10 |  | 5.62E-04 | 1.21E-02 | 1.03E-02 |
|  |  | Q06480 | psbN | Protein psbN | 1.39 | 1.02 | 1.13 |  | 3.73E-03 | 8.68E-01 | 4.89E-04 |
|  |  | A8J0E4 | PsbO | Oxygen-evolving enhancer protein 1 of photosystem II | 0.94 | 0.77 | 0.69 |  | 2.48E-01 | 2.71E-05 | 1.94E-06 |
|  |  | A8IXU9 | CGL30/PsbP | Photosystem II thylakoid lumenal 29.8 kDa protein PsbP | 7.48 | 7.99 | 7.70 |  | 5.69E-02 | 9.00E-02 | 4.35E-02 |
|  |  | A8IYH9 | PsbP1 | Oxygen-evolving enhancer protein 2 of photosystem II | 1.50 | 0.74 | 0.56 |  | 3.85E-01 | 4.36E-02 | 8.67E-02 |
|  |  | A8J3S9 | PsbP2 | PsbP-like protein | 1.01 | 0.51 | 0.67 |  | 9.11E-01 | 5.26E-02 | 4.86E-03 |
|  |  | A8IKE6 | PsbP3 | OEE2-like protein of thylakoid lumen | 1.05 | 0.42 | 0.36 |  | 8.85E-01 | 6.81E-04 | 1.04E-04 |
|  |  | A8IHH9 | PsbP6 | Lumen targeted protein | 1.12 | 0.59 | 0.43 |  | 6.13E-01 | 1.36E-02 | 1.46E-02 |
|  |  | A8JEV1 | PsbQ | Oxygen evolving enhancer protein 3 | 13.41 | 14.63 | 11.40 |  | 1.15E-02 | 5.20E-04 | 1.08E-03 |
|  |  | A8HXG5 | PsbR | 10 kDa photosystem II polypeptide | 0.78 | 0.42 | 0.27 |  | 2.42E-02 | 9.25E-03 | 1.62E-02 |
|  |  | A8JFQ7 | PsbW | Photosystem II reaction center W protein | 0.17 | 0.08 | 0.10 |  | 4.09E-05 | 1.33E-05 | 5.05E-05 |
|  |  | A8III5 | Psb28 | Photosystem II reaction center psb28 protein | 0.67 | 0.19 | 0.28 |  | 1.97E-03 | 9.04E-05 | 2.27E-05 |
|  |  | P12154 | psaA | Photosystem I P700 chlorophyll a apoprotein A1 | 0.08 | 3.02 | 3.59 |  | 2.68E-06 | 1.18E-02 | 3.27E-02 |
|  |  | P09144 | psaB | Photosystem I P700 chlorophyll a apoprotein A2 | 0.13 | 4.23 | 4.34 |  | 1.93E-05 | 5.52E-03 | 5.32E-03 |
|  |  | Q5NKW4 | PsaD | Photosystem I reaction center subunit II 20 kDa | 14.68 | 19.73 | 20.90 |  | 1.38E-03 | 1.29E-04 | 2.92E-07 |
|  |  | A8J4S1 | PsaF | Photosystem I reaction center subunit III | 2.31 | 0.99 | 0.69 |  | 1.59E-01 | 9.86E-01 | 3.07E-02 |
|  |  | A8JHN9 | PsaG | Photosystem I reaction center subunit V | 0.30 | 0.10 | 0.15 |  | 1.32E-03 | 2.72E-08 | 2.65E-05 |
|  |  | A8IH77 | PsaH | Subunit H of photosystem I | 12.48 | 9.65 | 9.76 |  | 1.05E-02 | 3.24E-02 | 1.49E-02 |
|  |  | P59777 | psaJ | Photosystem I reaction center subunit IX | 0.26 | 0.12 | 0.23 |  | 2.80E-06 | 1.73E-04 | 1.62E-02 |
|  |  | A8J6K8 | PsaK | Photosystem I reaction center subunit psaK | 0.51 | 0.18 | 0.12 |  | 2.34E-03 | 4.70E-05 | 5.15E-06 |
|  |  | A8I835 | PsaN | Photosystem I reaction center subunit N | 0.47 | 0.41 | 0.08 |  | 9.95E-04 | 3.27E-04 | 8.99E-07 |
|  |  | O20030 | ycf4 | Photosystem I assembly protein Ycf4 | 0.08 | 0.08 | 0.14 |  | 2.20E-08 | 6.55E-08 | 2.94E-06 |
|  |  | A8J9G6 | Cyc 6 | Cytochrome c6 | 3.06 | 0.46 | 1.20 |  | 3.13E-01 | 3.89E-02 | 8.36E-01 |
|  |  | P23577 | petA | Apocytochrome f | 1.77 | 0.71 | 0.91 |  | 2.57E-01 | 2.18E-02 | 5.15E-01 |
|  |  | Q00471 | petB | Cytochrome b6 | 0.27 | 0.42 | 0.61 |  | 2.97E-04 | 4.23E-05 | 2.67E-03 |
|  |  | A8IXV0 | CHLRE_03g198850v5 | Thylakoid lumen protein | 1.29 | 0.78 | 0.45 |  | 2.62E-01 | 2.74E-01 | 1.50E-02 |
|  |  | A8HZ72 | CGLD14 | Predicted protein | 0.48 | 0.18 | 0.38 |  | 1.92E-02 | 5.84E-04 | 2.94E-02 |
|  |  | A8HQJ5 | TEF30 | Predicted protein | 0.85 | 0.71 | 0.79 |  | 1.81E-01 | 7.00E-03 | 6.76E-02 |
|  |  | P00877 | Rbcl | Ribulose bisphosphate carboxylase large chain | 0.54 | 0.56 | 0.37 |  | 8.04E-02 | 7.53E-02 | 4.90E-02 |
|  |  | A8IYP4 | PRK1 | Phosphoribulokinase | 1.04 | 0.12 | 0.11 |  | 8.34E-01 | 2.66E-06 | 1.77E-09 |
|  |  | A8IKQ0 | FBP1 | Fructose-1,6-bisphosphatase | 0.66 | 0.28 | 0.07 |  | 1.41E-05 | 1.39E-04 | 5.99E-08 |
|  |  | A8I531 | CHLD | Magnesium chelatase subunit D | 0.20 | 0.15 | 0.12 |  | 9.07E-05 | 2.18E-06 | 3.19E-07 |
|  |  | A8IMZ5 | CHLI1 | Magnesium chelatase subunit I | 0.12 | 0.07 | 0.06 |  | 2.84E-09 | 5.58E-09 | 1.21E-07 |
|  |  | A8IKQ6 | CHLI2 | Magnesium chelatase subunit I | 0.18 | 0.09 | 0.16 |  | 5.76E-05 | 2.07E-07 | 7.13E-07 |
|  |  | A8IKK7 | CTH1\|CTH1B | Copper target 1 protein | 0.31 | 0.33 | 0.38 |  | 1.20E-02 | 1.50E-02 | 1.75E-02 |
|  |  | A8ITX0 | CRD1 | Copper response defect 1 protein | 1.55 | 0.40 | 0.60 |  | 7.13E-01 | 4.04E-02 | 4.62E-01 |
|  |  | A8HNE8 | CHLRE_01g050950v5 | Geranylgeranyl reductase | 0.23 | 0.13 | 0.09 |  | 5.09E-04 | 7.99E-06 | 2.98E-06 |
|  |  | A8HPJ2 | POR | Light-dependent protochlorophyllide reductase | 0.20 | 0.09 | 0.05 |  | 5.50E-05 | 1.39E-06 | 6.02E-12 |
|  |  | Q9AXF6 | LHCBM7 | Chlorophyll a-b binding protein of LHCII | 0.69 | 0.22 | 0.95 |  | 3.29E-04 | 1.81E-03 | 2.58E-02 |
|  |  | Q9ZSJ4 | LHCBM5 | Chlorophyll a-b binding protein of LHCII | 3.42 | 3.47 | 3.55 |  | 1.68E-02 | 1.06E-01 | 1.90E-03 |
|  |  | A8JCU4 | LHCBM1 | Chlorophyll a-b binding protein of LHCII | 4.45 | 5.59 | 5.91 |  | 1.40E-02 | 1.32E-01 | 1.36E-02 |
|  |  | A8J6D1 | CP29 | Chlorophyll a-b binding protein of photosystem II | 0.78 | 0.44 | 0.18 |  | 3.47E-01 | 2.26E-02 | 4.46E-07 |
|  |  | A8J287 | LHCBM6 | Chloropyll a-b binding protein of LHCII type I chloroplast | 0.79 | 0.53 | 0.59 |  | 1.77E-01 | 3.93E-02 | 4.87E-02 |
|  |  | A8J270 | LHCBM8 | Chlorophyll a-b binding protein of LHCII | 0.59 | 0.32 | 0.44 |  | 1.69E-01 | 5.95E-03 | 3.24E-02 |
|  |  | Q8S3T9 | LHCBM9 | Chlorophyll a-b binding protein of LHCII | 24.82 | 23.50 | 26.59 |  | 7.94E-05 | 4.68E-03 | 1.40E-04 |
|  |  | P93664 | LHCSR1 | Light-harvesting complex stress-related protein 1, chloroplastic | 9.42 | 28.18 | 39.15 |  | 5.31E-02 | 1.52E-02 | 1.49E-04 |
|  |  | A8J431 | LHCSR3 | Stress-related chlorophyll a/b binding protein 2 | 0.28 | 0.22 | 0.50 |  | 1.67E-03 | 4.82E-04 | 1.09E-01 |
|  |  | A8JF10 | LHCA3 | Light-harvesting chlorophyll-a/b protein of photosystem I type III | 0.72 | 0.61 | 0.59 |  | 3.46E-01 | 4.66E-02 | 1.45E-02 |
|  |  | A8I000 | LHCA4 | Light-harvesting protein of photosystem I | 4.15 | 4.68 | 5.35 |  | 5.31E-03 | 1.24E-01 | 1.07E-02 |
|  |  | A8ISG0 | LHCA7 | Light-harvesting protein of photosystem I | 0.55 | 0.44 | 0.70 |  | 3.43E-03 | 1.35E-02 | 5.96E-02 |
|  |  | A8ITV3 | LHCA9 | Light-harvesting protein of photosystem I | 0.62 | 0.34 | 0.48 |  | 9.44E-06 | 8.72E-04 | 1.61E-03 |
|  |  | Q75VY8 | LHCA5 | Light-harvesting chlorophyll-a/b protein of photosystem I | 0.62 | 0.65 | 1.12 |  | 1.18E-02 | 7.37E-02 | 6.31E-01 |
|  |  | Q75VY7 | LHCA8 | Light-harvesting chlorophyll-a/b protein of photosystem I | 2.54 | 2.27 | 3.87 |  | 1.04E-01 | 2.65E-01 | 2.54E-02 |
|  |  | A8J0A7 | ELI3 | Early light-inducible protein | 1.32 | 2.92 | 3.33 |  | 7.03E-01 | 3.19E-02 | 3.73E-02 |
|  |  | A8HMS2 | CAO | Chlorophyll a oxygenase | 0.78 | 0.43 | 0.65 |  | 6.75E-01 | 1.26E-02 | 3.06E-01 |
|  |  | A8JFJ1 | CHLG | Chlorophyll synthetase | 0.40 | 0.65 | 0.74 |  | 2.28E-02 | 1.03E-01 | 1.47E-01 |
|  |  | A8J7H3 | GSA | Chlorophyll synthetase | 0.06 | 0.04 | 0.06 |  | 1.27E-08 | 1.32E-09 | 1.23E-06 |
|  |  | A8I7P5 | CHLH1 | Magnesium chelatase subunit H | 0.06 | 0.12 | 0.14 |  | 6.32E-07 | 7.25E-06 | 3.51E-05 |
|  |  | A8JGJ6 | CHLM | Mg protoporphyrin IX S-adenosyl methionine O-methyl transferase | 0.28 | 0.18 | 0.42 |  | 7.01E-07 | 2.76E-04 | 2.98E-03 |
|  |  | A8J9S8 | PNP1 | Polyribonucleotide phosphorylase PNPase (Fragment) | 0.51 | 0.71 | 0.40 |  | 8.83E-04 | 3.27E-02 | 1.67E-04 |
|  |  | A8J9E9 | CHLREDRAFT_196597 | Carotenoid isomerase (Fragment) | 1.19 | 2.11 | 2.05 |  | 4.02E-01 | 1.55E-02 | 2.92E-03 |
|  |  | Q6J214 | PSY | Chloroplast phytoene synthase | 0.26 | 0.58 | 0.93 |  | 8.42E-04 | 5.61E-03 | 8.61E-01 |
|  |  | A8J261 | HST1 | Homogentisate solanesyltransferase (Fragment) | 0.42 | 0.53 | 0.49 |  | 4.70E-03 | 7.21E-03 | 2.44E-02 |
|  |  | A8HWI0 | LCYE | Lycopene epsilon cyclase | 0.14 | 0.21 | 0.38 |  | 3.51E-05 | 1.90E-04 | 1.49E-04 |
|  |  | A8I647 | ZDS1 | Zeta-carotene desaturase | 0.21 | 0.23 | 0.17 |  | 1.05E-05 | 1.01E-04 | 9.32E-06 |
| 3 | Redox homeostasis | A8IUG8 | CDSP32 | Plastidic thioredoxin-like protein | 0.90 | 0.76 | 0.35 |  | 5.06E-01 | 6.09E-03 | 1.48E-04 |
|  |  | A8J0Q8 | CITRX | Thioredoxin-related protein | 4.51 | 4.51 | 3.24 |  | 4.33E-03 | 4.25E-03 | 1.16E-01 |
|  |  | A8J594 | DLC3 | Flagellar outer dynein arm 16 kDa light chain LC3 | 3.34 | 3.22 | 1.65 |  | 4.94E-02 | 1.52E-01 | 8.43E-02 |
|  |  | A8HPL8 | DLD2 | Dihydrolipoamide dehydrogenase | 0.70 | 0.59 | 0.28 |  | 7.27E-02 | 2.07E-02 | 1.66E-04 |
|  |  | A8J1T4 | GCSL | Dihydrolipoyl dehydrogenase | 2.31 | 4.07 | 4.23 |  | 5.44E-02 | 1.55E-02 | 7.15E-03 |
|  |  | A8JHA9 | GRX1 | Glutaredoxin CPYC type | 3.21 | 3.64 | 3.10 |  | 5.87E-04 | 3.04E-08 | 9.41E-04 |
|  |  | A8IYH1 | GRX2 | Glutaredoxin CPYC type | 1.71 | 2.45 | 3.47 |  | 9.28E-02 | 1.72E-01 | 4.41E-02 |
|  |  | A8J916 | CHLRE_06g278183v5 | Glutaredoxin-like protein | 1.12 | 2.03 | 2.25 |  | 3.76E-01 | 5.84E-02 | 2.53E-02 |
|  |  | A8JH05 | GRX3 | Glutaredoxin CGFS type | 0.80 | 0.64 | 0.59 |  | 1.53E-01 | 2.43E-03 | 9.55E-02 |
|  |  | A8JIA7 | GRX4 | Glutaredoxin CGFS type | 0.78 | 1.05 | 1.12 |  | 5.34E-03 | 8.55E-01 | 3.13E-02 |
|  |  | A8HN52 | GRX6 | Glutaredoxin CGFS type | 0.51 | 0.15 | 0.12 |  | 2.43E-02 | 4.70E-06 | 6.67E-05 |
|  |  | A8J0E5 | GSHR2 | Glutathione reductase | 1.56 | 2.78 | 2.33 |  | 1.84E-02 | 1.51E-04 | 3.36E-02 |
|  |  | A8J6A7 | MET16/APR1 | Adenylylphosphosulfate reductase | 8.99 | 18.99 | 24.48 |  | 1.92E-05 | 5.61E-04 | 7.93E-04 |
|  |  | A8J9N7 | NRX4 | Nucleoredoxin | 1.19 | 1.65 | 1.69 |  | 1.90E-01 | 3.56E-03 | 1.72E-01 |
|  |  | A8HNQ7 | NTRC1 | Thioredoxin reductase | 0.35 | 0.53 | 1.19 |  | 3.21E-03 | 1.57E-02 | 5.05E-01 |
|  |  | O48949 | PDI | Protein disulfide isomerase | 9.02 | 8.22 | 10.25 |  | 8.36E-02 | 1.05E-01 | 4.16E-02 |
|  |  | A8HQT1 | PDI2 | Protein disulfide isomerase | 6.88 | 5.12 | 2.64 |  | 1.51E-02 | 8.55E-02 | 3.44E-02 |
|  |  | A8JBH7 | PDI3 | Protein disulfide isomerase | 1.43 | 0.42 | 0.38 |  | 1.20E-01 | 3.44E-02 | 1.49E-04 |
|  |  | A8IHI1 | PDI4 | Protein disulfide isomerase | 0.58 | 2.13 | 1.91 |  | 1.57E-02 | 2.01E-01 | 7.67E-02 |
|  |  | A8HZQ4 | PRX3 | Peroxiredoxin type II | 0.74 | 0.54 | 0.43 |  | 9.78E-02 | 3.21E-03 | 1.71E-04 |
|  |  | A8HPG8 | PRX5 | Peroxiredoxin type II | 4.75 | 4.11 | 3.29 |  | 6.62E-02 | 2.90E-02 | 3.55E-02 |
|  |  | A8ICK6 | PRX6 | Thioredoxin dependent peroxidase | 0.73 | 0.42 | 0.44 |  | 1.95E-01 | 8.74E-04 | 2.03E-03 |
|  |  | A8JIT5 | PRX7 | Peroxiredoxin | 2.56 | 3.38 | 3.54 |  | 4.76E-02 | 4.43E-02 | 3.40E-02 |
|  |  | A8JFC3 | SCO1 | Cytochrome c oxidase assembly factor | 1.60 | 1.87 | 2.13 |  | 2.78E-02 | 2.44E-02 | 2.45E-02 |
|  |  | Q9FE86 | PRX1 | 2-cys peroxiredoxin chloroplastic | 17.92 | 17.27 | 17.88 |  | 5.92E-03 | 7.49E-05 | 7.60E-04 |
|  |  | A8IZR5 | TRXh | Thioredoxin | 9.35 | 9.45 | 11.36 |  | 2.54E-02 | 3.55E-02 | 4.68E-02 |
|  |  | A8HP58 | TRXm | Thioredoxin | 1.39 | 1.18 | 0.17 |  | 4.53E-01 | 6.23E-01 | 7.60E-05 |
|  |  | Q84XS0 | TRXo | Thioredoxin o | 1.29 | 0.97 | 0.20 |  | 7.17E-02 | 9.14E-01 | 6.66E-05 |
|  |  | Q84XR9 | TRXx | Thioredoxin x | 9.23 | 8.98 | 9.09 |  | 1.58E-01 | 1.62E-02 | 4.23E-02 |
|  |  | A8IQA9 | CHLREDRAFT_11164 | Thioredoxin-like protein | 1.06 | 0.75 | 0.92 |  | 8.41E-01 | 2.32E-03 | 7.00E-01 |
|  |  | A8I0I4 | CHLRE_10g456250v5 | Thioredoxin-like protein | 0.56 | 0.60 | 0.67 |  | 9.46E-04 | 5.40E-03 | 9.08E-04 |
|  |  | A8JA70 | CHLRE_16g687294v5 | Ferredoxin thioredoxin reductase variable chain | 2.08 | 0.55 | 0.34 |  | 7.57E-02 | 2.07E-04 | 9.15E-04 |
|  |  | A8HUI8 | CHLREDRAFT_188073 | Predicted protein | 1.08 | 1.62 | 2.00 |  | 6.05E-01 | 2.81E-01 | 2.57E-03 |
|  |  | A8JDA2 | CHLRE_17g715500v5 | Predicted protein | 2.01 | 1.36 | 0.90 |  | 2.56E-02 | 1.82E-01 | 1.40E-01 |
|  |  | A8IC32 | CHLREDRAFT_141005 | Predicted protein | 0.47 | 0.67 | 0.97 |  | 2.39E-02 | 1.56E-01 | 8.75E-01 |
|  |  | A8JG35 | CHLREDRAFT_160132 | Predicted protein | 0.96 | 0.63 | 0.28 |  | 2.10E-01 | 1.79E-03 | 9.24E-05 |
|  |  | A8HUF5 | CHLREDRAFT_182752 | Predicted protein | 1.05 | 0.92 | 0.52 |  | 8.00E-01 | 6.64E-01 | 1.04E-02 |
|  |  | A8IHN7 | CHLREDRAFT_188533 | Predicted protein | 0.60 | 1.07 | 1.14 |  | 2.39E-02 | 8.03E-01 | 7.10E-01 |
|  |  | O49822 | apx1 | Ascorbate peroxidase | 4.57 | 5.70 | 5.27 |  | 2.90E-02 | 1.39E-04 | 1.03E-02 |
|  |  | A8J7X9 | CCPR1 | Cytochrome c peroxidase | 4.88 | 4.58 | 3.49 |  | 2.06E-03 | 5.60E-03 | 1.35E-02 |
|  |  | A8JG56 | CHLREDRAFT_165193 | L-ascorbate peroxidase | 1.26 | 0.62 | 0.14 |  | 1.43E-02 | 3.48E-03 | 1.58E-05 |
|  |  | A8J285 | APX2 | L-ascorbate peroxidase | 2.44 | 4.11 | 4.41 |  | 4.52E-02 | 1.69E-02 | 8.30E-02 |
|  |  | O81648 | Lci2 | Low CO2 inducible gene | 0.76 | 0.52 | 0.46 |  | 4.51E-01 | 7.40E-03 | 1.16E-02 |
|  |  | A8J537 | CAT1 | Catalase | 4.78 | 6.46 | 6.15 |  | 8.52E-03 | 6.37E-03 | 3.62E-03 |
|  |  | A8HN12 | CAT2 | Catalase/peroxidase | 2.23 | 3.01 | 3.26 |  | 8.57E-02 | 4.36E-02 | 2.14E-02 |
|  |  | A8IXD6 | CLPR4 | ATP-dependent Clp protease proteolytic subunit | 6.14 | 5.76 | 3.45 |  | 1.33E-02 | 8.21E-02 | 5.57E-03 |
| 4 | Protein folding | A8J524 | CCT2 | T-complex protein 1 beta subunit | 1.77 | 1.55 | 0.99 |  | 1.68E-03 | 2.18E-01 | 9.67E-01 |
|  |  | A8J7J2 | CCT5 | T-complex protein epsilon subunit | 1.23 | 2.58 | 1.74 |  | 4.37E-01 | 9.33E-03 | 2.36E-01 |
|  |  | A8HQ74 | CCT7 | T-complex protein eta subunit | 0.81 | 1.96 | 1.70 |  | 2.70E-01 | 3.16E-02 | 8.51E-02 |
|  |  | A8IF08 | CCT8 | T-complex protein theta subunit | 1.44 | 1.61 | 1.16 |  | 2.87E-01 | 1.81E-02 | 7.68E-01 |
|  |  | Q66YD3 | CDJ1 | Chloroplast DnaJ-like protein | 0.57 | 0.57 | 1.14 |  | 1.24E-03 | 2.13E-02 | 4.62E-01 |
|  |  | A8J594 | DLC3 | Flagellar outer dynein arm 16 kDa light chain LC3 | 3.34 | 3.22 | 1.65 |  | 4.94E-02 | 1.52E-01 | 8.43E-02 |
|  |  | A8IQC5 | DNJ1 | DnaJ-like protein | 0.31 | 1.13 | 1.39 |  | 1.23E-02 | 5.25E-01 | 3.77E-01 |
|  |  | A8HMC0 | CRT2 | Calreticulin 2 calcium-binding protein | 1.17 | 0.48 | 0.22 |  | 6.52E-01 | 4.65E-03 | 2.77E-05 |
|  |  | A8IV02 | CYN1a\|CYN1b | Peptidyl-prolyl cis-transisomerase cyclophilin type | 0.97 | 0.87 | 0.76 |  | 7.69E-01 | 3.07E-01 | 5.54E-03 |
|  |  | A8JD64 | CYN19-2 | Peptidyl-prolyl cis-trans isomerase | 0.88 | 0.51 | 0.29 |  | 6.04E-01 | 1.44E-02 | 1.23E-02 |
|  |  | A8HUU9 | CYN19-3 | Peptidyl-prolyl cis-trans isomerase | 0.62 | 0.69 | 0.34 |  | 8.03E-02 | 1.72E-01 | 1.52E-03 |
|  |  | A8J282 | CYN20-1 | Peptidyl-prolyl cis-trans isomerase | 2.93 | 5.91 | 2.59 |  | 2.41E-02 | 7.34E-04 | 7.15E-02 |
|  |  | A8JDL5 | CYN20-3 | Peptidyl-prolyl cis-trans isomerase | 1.18 | 0.60 | 0.51 |  | 7.68E-01 | 1.82E-02 | 2.59E-02 |
|  |  | A8ID98 | CYN20-5 | Peptidyl-prolyl cis-trans isomerase cyclophilin-type | 0.67 | 0.66 | 0.98 |  | 3.63E-03 | 1.61E-01 | 9.23E-01 |
|  |  | A8JFN4 | CYN23a\|CYN23b | Cyclophilin-like protein | 2.26 | 1.79 | 1.30 |  | 6.35E-02 | 1.13E-01 | 4.40E-02 |
|  |  | A8IE53 | CYN26 | Peptidyl-prolyl cis-trans isomerase cyclophilin-type | 0.65 | 0.35 | 0.38 |  | 7.37E-03 | 1.92E-04 | 5.52E-04 |
|  |  | A8JHN8 | CYN28 | Peptidyl-prolyl cis-trans isomerase cyclophilin-type | 0.95 | 0.46 | 0.30 |  | 6.72E-01 | 6.46E-03 | 2.90E-03 |
|  |  | A8INE5 | CYN37 | Peptidyl-prolyl cis-trans isomerase cyclophilin-type | 1.02 | 0.46 | 0.72 |  | 9.55E-01 | 2.30E-03 | 2.67E-01 |
|  |  | A8I0M0 | CYN65 | Peptidyl-prolyl cis-trans isomerase cyclophilin-type | 1.12 | 0.59 | 0.98 |  | 8.96E-02 | 3.69E-02 | 8.87E-01 |
|  |  | A8I6Y0 | ERJ1 | ER DnaJ-like protein 1 | 0.65 | 0.54 | 0.70 |  | 2.13E-02 | 5.09E-03 | 2.81E-05 |
|  |  | A8J3C1 | FKB16-7a\|FKB16-7b | Peptidyl-prolyl cis-trans isomerase | 0.61 | 0.29 | 0.35 |  | 3.54E-05 | 2.49E-04 | 2.07E-03 |
|  |  | A8J3L6 | FKB16-2a\|FKB16-2c\|FKB16-2b | Peptidyl-prolyl cis-trans isomerase | 1.22 | 0.73 | 0.45 |  | 1.29E-01 | 8.34E-02 | 1.91E-03 |
|  |  | A8I9C7 | FKB42 | Peptidyl-prolyl cis-trans isomerase FKBP-type | 1.32 | 1.77 | 1.47 |  | 5.84E-03 | 1.80E-03 | 4.82E-01 |
|  |  | A8J1U1 | HSP90A | Heat shock protein 90A | 3.10 | 4.59 | 3.90 |  | 7.22E-02 | 1.19E-02 | 5.60E-02 |
|  |  | A8I7T1 | HSP90B | Heat shock protein 90B | 2.24 | 2.85 | 3.28 |  | 9.60E-02 | 5.77E-03 | 6.40E-03 |
|  |  | Q66T67 | HSP90C | Heat shock protein 90C | 0.75 | 0.42 | 0.36 |  | 3.83E-01 | 3.92E-03 | 1.99E-03 |
|  |  | Q944P3 | HSP33 | Heat shock protein 33 | 0.54 | 0.32 | 0.62 |  | 7.90E-04 | 9.03E-04 | 1.65E-02 |
|  |  | A8JES1 | MGE1 | GrpE protein homolog | 3.31 | 2.63 | 1.05 |  | 8.42E-03 | 4.65E-03 | 8.55E-01 |
|  |  | A8HPB8 | MSRA4 | Peptidyl-prolyl cis-trans isomerase, FKBP-type | 0.76 | 0.38 | 0.19 |  | 2.49E-01 | 3.46E-07 | 2.35E-06 |
|  |  | A8JDH3 | NUDC | Nuclear movement family protein | 0.87 | 0.60 | 0.40 |  | 4.12E-01 | 1.43E-01 | 5.88E-03 |
|  |  | A8JBH7 | PDI3 | Protein disulfide isomerase | 1.43 | 0.42 | 0.38 |  | 1.20E-01 | 3.44E-02 | 1.49E-04 |
|  |  | A8IHI1 | PDI4 | Protein disulfide isomerase | 0.58 | 2.13 | 1.91 |  | 1.57E-02 | 2.01E-01 | 7.67E-02 |
|  |  | A8JD56 | TIG1 | Chloroplast trigger factor | 0.54 | 0.26 | 0.30 |  | 1.57E-03 | 2.78E-07 | 4.16E-03 |
|  |  | A8IZR5 | TRXh | Thioredoxin | 9.35 | 9.45 | 11.36 |  | 2.54E-02 | 3.55E-02 | 4.68E-02 |
|  |  | A8HP58 | TRXm | Thioredoxin | 1.39 | 1.18 | 0.17 |  | 4.53E-01 | 6.23E-01 | 7.60E-05 |
|  |  | Q84XS0 | TRXo | Thioredoxin o | 1.29 | 0.97 | 0.20 |  | 7.17E-02 | 9.14E-01 | 6.66E-05 |
|  |  | Q84XR9 | TRXx | Thioredoxin x | 9.23 | 8.98 | 9.09 |  | 1.58E-01 | 1.62E-02 | 4.23E-02 |
|  |  | A8J0Q8 | CITRX | Thioredoxin-related protein CITRX | 4.51 | 4.51 | 3.24 |  | 4.33E-03 | 4.25E-03 | 1.16E-01 |
|  |  | A8IQA9 | CHLREDRAFT_11164 | Thioredoxin-like protein | 1.06 | 0.75 | 0.92 |  | 8.41E-01 | 2.32E-03 | 7.00E-01 |
|  |  | A8I0I4 | CHLRE_10g456250v5 | Thioredoxin-like protein | 0.56 | 0.60 | 0.67 |  | 9.46E-04 | 5.40E-03 | 9.08E-04 |
|  |  | A8I211 | CHLREDRAFT_161043 | Predicted protein | 1.84 | 2.17 | 1.99 |  | 7.90E-03 | 1.03E-02 | 9.29E-03 |
|  |  | A8IHN7 | CHLREDRAFT_188533 | Predicted protein | 0.60 | 1.07 | 1.14 |  | 2.39E-02 | 8.03E-01 | 7.10E-01 |
|  |  | A8HUK0 | FKB12 | Peptidyl-prolyl cis-trans isomerase | 0.81 | 0.29 | 0.23 |  | 3.94E-01 | 5.31E-06 | 3.19E-05 |
|  |  | A8J746 | FKB15-1 | Peptidyl-prolyl cis-trans isomerase | 0.66 | 0.31 | 0.23 |  | 2.28E-02 | 1.63E-04 | 2.25E-04 |
|  |  | A8J740 | FKB15-4 | Peptidyl-prolyl cis-trans isomerase | 0.64 | 0.13 | 0.13 |  | 1.12E-01 | 9.30E-06 | 3.53E-06 |
|  |  | A8I6B6 | FKB16-1 | Peptidyl-prolyl cis-trans isomerase | 0.77 | 0.33 | 0.29 |  | 4.16E-01 | 1.67E-05 | 4.69E-04 |
|  |  | A8J3L3 | FKB16-5 | Peptidyl-prolyl cis-trans isomerase | 0.75 | 0.42 | 0.17 |  | 1.23E-01 | 2.38E-05 | 4.12E-04 |
|  |  | A8JEI4 | FKB16-8 | Peptidyl-prolyl cis-trans isomerase | 0.76 | 0.37 | 0.29 |  | 3.34E-02 | 3.59E-05 | 7.49E-04 |
|  |  | A8J1E5 | FKB17-1 | Peptidyl-prolyl cis-trans isomerase | 0.69 | 0.37 | 0.40 |  | 1.90E-01 | 5.38E-03 | 2.19E-02 |
|  |  | A8JEK6 | FKB17-2 | Peptidyl-prolyl cis-trans isomerase | 0.51 | 0.73 | 0.68 |  | 3.17E-02 | 1.65E-01 | 2.50E-03 |
|  |  | A8I1U5 | FKB18 | Peptidyl-prolyl cis-trans isomerase | 0.91 | 0.37 | 0.37 |  | 6.19E-01 | 3.01E-09 | 1.07E-04 |
|  |  | A8IVN2 | FKB19 | Peptidyl-prolyl cis-trans isomerase | 4.87 | 2.13 | 1.35 |  | 5.09E-04 | 1.67E-01 | 3.73E-01 |
|  |  | A8JAS8 | FKB53 | Peptidyl-prolyl cis-trans isomerase | 1.01 | 0.56 | 0.69 |  | 9.70E-01 | 4.54E-02 | 1.83E-01 |
|  |  | A8J0I6 | FKB62 | Peptidyl-prolyl cis-trans isomerase FKBP-type | 0.72 | 0.84 | 0.84 |  | 2.96E-02 | 1.46E-01 | 6.95E-02 |
|  |  | A8HPN3 | FKB99 | Peptidyl-prolyl cis-trans isomerase FKBP-type | 1.07 | 1.09 | 1.43 |  | 2.48E-01 | 5.21E-01 | 4.57E-02 |
|  |  | A8I9E1 | TPR1 | Predicted chloroplast-targeted protein | 0.68 | 0.54 | 0.44 |  | 2.45E-01 | 7.79E-02 | 4.88E-02 |
| 5 | Intracellular protein trafficking | A8J729 | AP1B1 | Putative uncharacterized protein AP1B1 | 1.97 | 3.71 | 3.03 |  | 1.77E-01 | 5.78E-03 | 1.06E-03 |
|  |  | A8IMC5 | AP4E1 | Epsilon-adaptin | 0.76 | 1.70 | 1.68 |  | 2.32E-01 | 1.85E-02 | 2.22E-03 |
|  |  | A8IZV8 | AP1S1 | Sigma1-Adaptin | 1.53 | 1.70 | 1.51 |  | 1.73E-05 | 4.03E-02 | 6.54E-02 |
|  |  | A8HXA2 | AP1M1 | Mu1-Adaptin | 0.93 | 1.97 | 1.44 |  | 7.75E-01 | 3.14E-03 | 6.76E-02 |
|  |  | A8ILF4 | AP2A1 | Alpha-adaptin | 1.25 | 3.37 | 2.95 |  | 2.67E-01 | 8.19E-03 | 1.72E-03 |
|  |  | A8J7K1 | CLC1 | Clathrin light chain | 3.43 | 2.41 | 1.44 |  | 3.25E-02 | 4.35E-02 | 2.28E-01 |
|  |  | A8I4S9 | CHC1 | Clathrin heavy chain | 1.03 | 8.04 | 6.58 |  | 9.30E-01 | 2.29E-02 | 7.17E-03 |
|  |  | A8HRR9 | COPA1 | Alpha-COP | 1.42 | 5.23 | 4.70 |  | 3.56E-01 | 9.10E-03 | 3.75E-03 |
|  |  | A8JEP9 | COPB1 | Coatomer subunit beta | 0.66 | 4.04 | 2.90 |  | 2.26E-01 | 4.14E-02 | 5.71E-02 |
|  |  | A8JGS8 | COPB2 | Beta-cop | 2.31 | 4.05 | 2.41 |  | 1.14E-02 | 1.44E-03 | 1.87E-02 |
|  |  | A8JCA4 | CHLREDRAFT_195581 | RabGAP/TBC protein | 3.94 | 7.18 | 6.10 |  | 3.33E-02 | 3.28E-06 | 1.06E-04 |
|  |  | A8JC30 | SAR1 | Sar-type small GTPase | 1.11 | 1.03 | 0.80 |  | 2.60E-01 | 5.01E-01 | 1.10E-02 |
|  |  | A8HZC8 | SEC23B | COP-II coat subunit | 0.93 | 2.06 | 1.23 |  | 1.67E-01 | 1.55E-01 | 1.55E-02 |
|  |  | A8I985 | SEC24A | COP-II coat subunit | 0.68 | 1.59 | 1.88 |  | 4.24E-02 | 6.24E-02 | 9.95E-02 |
|  |  | A8I1Y7 | SNAPA1 | Alpha-SNAP | 3.10 | 5.13 | 5.41 |  | 1.28E-02 | 8.95E-03 | 1.46E-03 |
|  |  | Q8S4W5 | SYP6 | Qc-SNARE protein Tlg1/Syntaxin 6-family | 2.79 | 3.31 | 2.84 |  | 7.32E-03 | 4.36E-04 | 1.96E-03 |
|  |  | A8IR64 | SYP5 | Qc-SNARE protein Syn8/Syntaxin8-family | 3.57 | 5.29 | 4.91 |  | 1.89E-02 | 4.18E-02 | 1.21E-02 |
|  |  | A8JBN4 | VCL1 | VPS16-like protein | 0.66 | 0.72 | 1.37 |  | 1.12E-01 | 4.01E-01 | 4.83E-02 |
|  |  | A8J4X1 | VPS26 | Subunit of retromer complex | 4.28 | 3.71 | 2.75 |  | 6.01E-03 | 1.76E-02 | 3.48E-03 |
|  |  | A8HQF0 | VPS35 | Subunit of retromer complex | 1.62 | 2.51 | 2.35 |  | 4.46E-03 | 3.29E-02 | 3.29E-02 |
|  |  | A8JCM8 | VPS45 | SM/Sec1-family protein | 2.30 | 2.25 | 2.10 |  | 4.03E-02 | 7.28E-02 | 2.18E-01 |
|  |  | A8I211 | CHLREDRAFT_161043 | Predicted protein | 1.84 | 2.17 | 1.99 |  | 7.90E-03 | 1.03E-02 | 9.29E-03 |
|  |  | A8I3S5 | CHLRE_02g108050v5 | Predicted protein | 2.46 | 3.82 | 4.44 |  | 2.51E-02 | 3.24E-02 | 1.81E-02 |
|  |  | A8IYJ6 | CGL38 | Predicted protein | 1.44 | 0.34 | 0.33 |  | 7.21E-02 | 1.84E-03 | 2.10E-03 |
|  |  | A8IAJ1 | VPS4 | AAA-ATPase of VPS4/SKD1 family | 0.90 | 0.94 | 0.49 |  | 3.58E-01 | 5.48E-01 | 3.00E-02 |
|  |  | A8HSQ2 | FAP66 | Flagellar associated protein | 0.66 | 2.44 | 1.86 |  | 2.39E-03 | 4.85E-02 | 2.44E-02 |
|  |  | A8J7M4 | CHLREDRAFT_150402 | Predicted protein (Fragment) | 0.74 | 1.40 | 1.39 |  | 1.45E-02 | 7.61E-03 | 2.04E-02 |
|  |  | A8IYH0 | CHLRE_12g549950v5 | Tetraspanning membrane protein SFT2-like protein | 0.14 | 0.48 | 0.53 |  | 1.86E-04 | 3.32E-02 | 7.95E-03 |
|  |  | A8I023 | TIC110 | 110 kDa translocon of chloroplast envelope inner membrane (Fragment) | 0.20 | 0.41 | 0.29 |  | 5.34E-07 | 6.72E-04 | 1.63E-03 |
|  |  | A8IJ46 | RNB2 | 3'-5' exoribonuclease II (Fragment) | 0.77 | 0.72 | 0.65 |  | 2.53E-01 | 8.61E-03 | 1.60E-01 |
|  |  | Q6Y682 | Rap38 | 38 kDa ribosome-associated protein | 1.05 | 0.70 | 0.38 |  | 7.94E-03 | 1.96E-02 | 9.83E-03 |
|  |  | A8IA39 | EFG1 | Chloroplast elongation factor G | 0.36 | 0.45 | 0.15 |  | 1.35E-03 | 6.65E-03 | 9.62E-05 |
|  |  | Q5S7Y5 | TIM | Chloroplast triosephosphate isomerase | 2.65 | 4.02 | 3.91 |  | 1.42E-01 | 8.98E-03 | 3.29E-02 |
|  |  | A8J680 | SECA2 | Chloroplast-associated SecA protein (Fragment) | 0.70 | 0.76 | 0.71 |  | 3.95E-02 | 1.76E-02 | 7.94E-02 |
|  |  | A8J6V5 | CHLREDRAFT_205591 | Diaminohydroxyphosphoribosylaminopyrimidine deaminase | 0.71 | 0.62 | 0.74 |  | 1.39E-01 | 2.62E-02 | 1.08E-02 |
|  |  | A8IZX1 | TEF24 | LrgB-like protein (Fragment) | 0.46 | 1.14 | 1.62 |  | 3.72E-02 | 3.86E-01 | 1.09E-01 |
|  |  | A8HWL8 | CHLREDRAFT_142189 | Predicted protein | 1.05 | 1.69 | 2.28 |  | 8.62E-01 | 4.25E-02 | 7.42E-02 |
|  |  | A8IB12 | CHLREDRAFT_126186 | Predicted protein (Fragment) | 0.91 | 0.83 | 0.54 |  | 3.45E-01 | 3.93E-02 | 1.89E-01 |
|  |  | A8J209 | CGL59 | Predicted protein | 0.36 | 0.41 | 0.49 |  | 3.62E-03 | 1.03E-02 | 1.29E-02 |
|  |  | A8J682 | SECA1 | Chloroplast-associated SecA protein | 0.38 | 0.69 | 0.78 |  | 6.39E-03 | 6.16E-02 | 9.33E-02 |
| 6 | TCA and ATP production | Q96550 | atpA | ATP synthase subunit alpha | 3.11 | 3.36 | 3.25 |  | 2.40E-02 | 1.01E-03 | 3.40E-02 |
|  |  | Q37304 | atpH | ATP synthase subunit c chloroplastic | 0.30 | 0.22 | 0.31 |  | 1.10E-04 | 7.18E-04 | 1.23E-05 |
|  |  | A8ID12 | ATP1B | Mitochondrial F1F0 ATP synthase alpha subunit (Fragment) | 0.77 | 0.75 | 0.41 |  | 2.81E-01 | 1.47E-01 | 2.94E-04 |
|  |  | A8HX15 | CHLREDRAFT_187139 | Sodium/potassium-transporting ATPase alpha subunit | 2.93 | 12.74 | 7.50 |  | 1.99E-04 | 2.51E-02 | 7.30E-03 |
|  |  | A8I164 | ATPvA1 | Vacuolar ATP synthase subunit A | 2.58 | 5.07 | 3.97 |  | 1.88E-02 | 7.24E-03 | 1.80E-02 |
|  |  | A8IA45 | ATPvB | Vacuolar ATP synthase subunit B | 4.48 | 3.30 | 2.45 |  | 1.46E-02 | 4.10E-02 | 2.93E-01 |
|  |  | A8IW47 | ATPvE | Vacuolar ATP synthase subunit E | 2.81 | 2.43 | 2.00 |  | 1.08E-02 | 6.37E-03 | 4.98E-02 |
|  |  | A8IDY0 | ATPvD1 | Vacuolar H+ ATPase V0 sector subunit D | 3.37 | 3.84 | 2.71 |  | 7.69E-03 | 7.00E-02 | 1.37E-01 |
|  |  | A8IST3 | ATPvA3 | Vacuolar proton ATPase subunit A | 1.27 | 3.97 | 3.96 |  | 4.52E-01 | 1.88E-03 | 3.35E-02 |
|  |  | A8J1K0 | ATPvA2 | Vacuolar proton translocating ATPase subunit A | 1.39 | 4.26 | 3.48 |  | 2.66E-01 | 1.82E-03 | 1.65E-03 |
|  |  | A8J588 | ATPvL2 | Vacuolar proton-ATPase subunit c'' proteolipid | 0.67 | 1.30 | 1.74 |  | 4.94E-02 | 2.88E-01 | 8.54E-02 |
|  |  | A8HZ87 | ATPvF | V-type proton ATPase subunit F | 1.81 | 1.88 | 1.20 |  | 8.71E-03 | 1.05E-04 | 5.45E-01 |
|  |  | Q8RVB8 | ATP6 | ATP synthase F1F0 subunit 6 | 1.51 | 0.89 | 1.05 |  | 2.24E-02 | 2.16E-01 | 6.20E-01 |
|  |  | A8HXL8 | ATPC | ATP synthase gamma chain | 7.84 | 12.47 | 14.71 |  | 6.64E-02 | 1.13E-01 | 3.25E-02 |
|  |  | o63075 | atpI | ATP synthase subunit a chloroplastic | 0.49 | 0.37 | 0.79 |  | 3.98E-05 | 9.00E-03 | 2.23E-02 |
|  |  | Q8HTL5 | atpF | ATP synthase subunit b chloroplastic | 0.61 | 0.80 | 0.99 |  | 3.06E-06 | 4.11E-01 | 7.23E-01 |
|  |  | A8ICT4 | ATP15 | F1F0 ATP synthase epsilon subunit | 2.66 | 0.93 | 0.44 |  | 2.44E-01 | 8.70E-01 | 4.27E-04 |
|  |  | A8J3U9 | ATP5 | Mitochondrial ATP synthase subunit 5 OSCP subunit | 3.92 | 1.84 | 2.66 |  | 5.64E-03 | 7.44E-03 | 1.54E-02 |
|  |  | A8J9X1 | ATP4 | Mitochondrial F1F0 ATP synthase delta subunit | 0.88 | 0.26 | 0.25 |  | 5.45E-01 | 1.24E-04 | 1.38E-05 |
|  |  | A8IVG0 | OGD1 | 2-oxoglutarate dehydrogenase E1 subunit | 1.32 | 3.89 | 3.32 |  | 5.12E-01 | 4.02E-02 | 2.90E-02 |
|  |  | A8HMQ1 | ACH1 | Aconitate hydratase | 1.47 | 2.98 | 2.00 |  | 2.43E-01 | 1.37E-02 | 6.05E-02 |
|  |  | A8JHC9 | CIS1 | Citrate synthase | 12.65 | 14.93 | 15.41 |  | 3.20E-03 | 8.86E-03 | 2.76E-03 |
|  |  | A8J2S0 | CIS2 | Citrate synthase | 0.30 | 0.27 | 0.19 |  | 3.32E-05 | 6.08E-04 | 9.34E-05 |
|  |  | A8HX04 | SDH2 | Iron-sulfur subunit of mitochondrial succinate dehydrogenase | 11.93 | 12.17 | 9.00 |  | 2.54E-02 | 4.14E-03 | 4.07E-03 |
|  |  | A8J9S7 | IDH3 | Isocitrate dehydrogenase [NADP] | 4.22 | 5.58 | 2.85 |  | 8.92E-02 | 4.00E-03 | 3.96E-02 |
|  |  | A8J0R7 | IDH2 | Isocitrate dehydrogenase NAD-dependent | 1.74 | 1.00 | 0.70 |  | 3.60E-02 | 9.75E-01 | 2.15E-01 |
|  |  | A8ICG9 | MDH2 | Malate dehydrogenase | 0.15 | 0.75 | 0.86 |  | 1.65E-06 | 2.22E-01 | 2.80E-01 |
|  |  | A8J0W9 | MDH3 | Malate dehydrogenase | 0.98 | 1.87 | 2.17 |  | 8.80E-01 | 1.34E-02 | 4.93E-03 |
|  |  | A8JHU0 | MDH4 | Malate dehydrogenase | 9.33 | 11.97 | 9.63 |  | 4.38E-02 | 8.14E-03 | 9.98E-03 |
|  |  | Q6X898 | MAS1 | Malate synthase | 0.56 | 0.18 | 0.20 |  | 1.14E-03 | 2.56E-04 | 7.34E-04 |
|  |  | Q9FNS5 | NADP-mdh | NADP-Malate dehydrogenase | 0.26 | 0.25 | 0.32 |  | 4.42E-06 | 9.68E-05 | 3.02E-04 |
|  |  | A8HP06 | SDH1 | Succinate dehydrogenase subunit A | 1.52 | 1.74 | 1.12 |  | 2.13E-04 | 1.63E-03 | 7.47E-01 |
|  |  | A8HPU1 | SDH4 | Succinate dehydrogenase subunit D | 0.78 | 0.57 | 0.42 |  | 1.10E-01 | 1.99E-03 | 9.67E-06 |
| 7 | Amino acid and sulfur metabolism | A8J6Q7 | SHKA1 | 3-deoxy-D-arabino-heptulosonate 7-phosphate synthetase | 0.26 | 0.44 | 0.45 |  | 1.51E-04 | 1.26E-05 | 1.73E-02 |
|  |  | A8JH48 | SHKG1 | 3-phosphoshikimate 1-carboxyvinyltransferase | 0.13 | 0.25 | 0.39 |  | 3.77E-06 | 4.41E-04 | 1.04E-03 |
|  |  | A8J2Z6 | SHKH1 | Chorismate synthase | 0.65 | 0.45 | 0.48 |  | 1.75E-01 | 1.38E-02 | 7.97E-04 |
|  |  | A8J434 | OASTL1 | Cysteine synthase | 0.60 | 0.56 | 0.58 |  | 8.62E-03 | 5.61E-03 | 3.39E-02 |
|  |  | A8IEE5 | OASTL3 | Cysteine synthase | 2.69 | 3.01 | 3.11 |  | 1.34E-01 | 9.87E-02 | 5.00E-02 |
|  |  | A8ISA9 | OASTL4 | Cysteine synthase | 19.91 | 19.97 | 20.34 |  | 3.46E-04 | 4.81E-03 | 1.22E-03 |
|  |  | A8JG03 | LEU1L | Isopropylmalate dehydratase large subunit | 1.62 | 3.80 | 7.11 |  | 1.53E-01 | 1.04E-01 | 8.03E-05 |
|  |  | A8IFZ9 | MAA7 | Tryptophan synthase beta subunit | 0.44 | 0.56 | 0.36 |  | 2.95E-05 | 4.42E-02 | 1.22E-04 |
|  |  | A8I9R1 | THD1 | Threonine deaminase | 0.28 | 0.42 | 0.46 |  | 5.15E-04 | 3.69E-03 | 7.39E-03 |
|  |  | A8HPI1 | AGK1 | Acetylglutamate kinase-like protein | 7.02 | 5.19 | 5.30 |  | 8.98E-03 | 6.80E-02 | 2.32E-03 |
|  |  | A8IRD4 | PROB2 | Glutamate 5-kinase | 0.45 | 0.55 | 0.39 |  | 2.53E-02 | 1.72E-02 | 1.26E-02 |
|  |  | A8IRD5 | PROB1 | Glutamate 5-kinase | 0.70 | 0.76 | 0.87 |  | 1.63E-02 | 1.76E-04 | 6.18E-01 |
|  |  | A8J2W0 | GSD1 | Glutamic-gamma-semialdehyde dehydrogenase | 0.33 | 1.25 | 1.21 |  | 9.03E-05 | 9.32E-02 | 1.59E-02 |
|  |  | A8HTR6 | CHLRE_13g579800v5 | Predicted amino acid kinase (Fragment) | 0.37 | 0.50 | 0.33 |  | 2.03E-05 | 4.03E-02 | 1.69E-02 |
|  |  | A8IBN0 | PRT1 | Anthranilate phosphoribosyltransferase | 0.44 | 0.31 | 0.39 |  | 9.34E-03 | 6.67E-03 | 1.99E-02 |
|  |  | A8IMY5 | ANS1 | Anthranilate synthase alpha subunit (Fragment) | 0.74 | 0.48 | 0.63 |  | 1.11E-01 | 5.91E-03 | 5.49E-02 |
|  |  | A8HP28 | ASB1 | Anthranilate synthase beta subunit | 1.17 | 0.72 | 1.21 |  | 1.81E-01 | 1.32E-02 | 2.55E-01 |
|  |  | A8J599 | TSA1 | Tryptophan synthetase alpha subunit | 0.57 | 0.55 | 0.47 |  | 1.90E-02 | 7.27E-02 | 2.38E-02 |
|  |  | A8HT60 | IGS1 | Indole-3-glycerol-phosphate synthase | 0.76 | 0.63 | 0.53 |  | 2.68E-03 | 8.92E-05 | 2.06E-02 |
|  |  | A8J3Q6 | APK1 | Adenylyl-sulfate kinase | 2.75 | 2.17 | 2.09 |  | 4.66E-02 | 1.61E-01 | 3.67E-02 |
|  |  | A8IXF1 | ATS1 | ATP-sulfurylase | 1.04 | 4.89 | 4.87 |  | 9.13E-01 | 1.16E-01 | 4.55E-03 |
|  |  | A8I3V3 | ATS2 | ATP-sulfurylase | 8.52 | 17.05 | 18.20 |  | 2.18E-03 | 1.36E-02 | 1.07E-02 |
|  |  | A8HYU5 | METM | S-adenosylmethionine synthase | 0.68 | 0.41 | 0.13 |  | 9.51E-02 | 1.27E-02 | 3.02E-06 |
|  |  | A8IXE0 | SAH1 | Adenosyl homocysteinase | 7.50 | 5.86 | 1.39 |  | 2.25E-03 | 1.26E-04 | 5.19E-01 |
|  |  | A8HRS5 | CHLREDRAFT_127560 | N^10^-formyltetrahydrofolate synthetase | 1.22 | 0.60 | 0.29 |  | 4.19E-01 | 1.14E-03 | 1.05E-03 |
|  |  | A8IQS8 | CHLREDRAFT_206121 | Methylenetetrahydrofolate dehydrogenase/  methylenetetrahydrofolate cyclohydrolase | 0.79 | 0.63 | 0.56 |  | 5.94E-03 | 6.28E-02 | 4.36E-02 |
|  |  | A8IAY6 | CHLREDRAFT_111330 | Methylenetetrahydrofolate reductase | 0.95 | 0.54 | 0.22 |  | 5.54E-01 | 4.96E-02 | 1.52E-04 |
|  |  | A8J4Z8 | CAH3 | Carbonic anhydrase 3 | 0.76 | 0.26 | 0.23 |  | 2.84E-01 | 1.09E-04 | 4.77E-04 |
|  |  | A8IT01 | CAH1 | Carbonic anhydrase | 0.27 | 0.23 | 0.28 |  | 1.80E-03 | 1.08E-04 | 5.24E-05 |
|  |  | A8IAK9 | PYR2 | Aspartate carbamoyltransferase | 0.56 | 0.30 | 0.36 |  | 1.39E-01 | 2.58E-03 | 1.37E-04 |
|  |  | A8IMN5 | CMPS1 | Carbamoyl phosphate synthase small subunit | 1.73 | 1.82 | 1.52 |  | 3.17E-01 | 5.80E-06 | 1.15E-01 |
|  |  | A8JBN5 | PYR4 | Dihydropryrimidine dehydrogenase | 1.86 | 2.62 | 3.10 |  | 2.65E-02 | 2.81E-02 | 1.80E-02 |
|  |  | A8JJR9 | FAK2 | Flagellar adenylate kinase (Fragment) | 1.51 | 10.07 | 7.44 |  | 4.72E-01 | 2.89E-02 | 9.32E-02 |
|  |  | A8HN92 | PYR5 | Uridine 5'-monophosphate synthase | 1.23 | 2.15 | 1.50 |  | 3.78E-02 | 2.64E-02 | 2.08E-01 |
| 8 | Response to cytokinin | A8J3F8 | CHLRE_16g672750v5 | Predicted protein | 0.58 | 0.25 | 0.23 |  | 4.94E-04 | 1.92E-06 | 7.76E-06 |
|  |  | A8JF10 | LHCA3 | Light-harvesting chlorophyll-a/b protein of photosystem I type III | 0.72 | 0.61 | 0.59 |  | 3.46E-01 | 4.66E-02 | 1.45E-02 |
|  |  | Q70DX8 | S1 | Plastid ribosomal protein S1 | 0.49 | 0.19 | 0.13 |  | 6.83E-03 | 4.02E-05 | 1.11E-04 |
|  |  | A8IRG9 | CPLD28 | Predicted protein | 0.91 | 0.74 | 0.91 |  | 1.03E-01 | 3.28E-02 | 3.33E-01 |
|  |  | A8HWZ6 | PRPL13 | Plastid ribosomal protein L13 | 0.32 | 0.08 | 0.08 |  | 3.65E-04 | 1.59E-08 | 4.22E-07 |
|  |  | A8IU62 | PIN3 | Peptidyl-prolyl cis-trans isomerase parvulin-type | 0.63 | 0.58 | 0.57 |  | 2.36E-03 | 2.44E-02 | 4.45E-02 |
|  |  | A8HXG5 | PSBR | 10 kDa photosystem II polypeptide | 0.78 | 0.42 | 0.27 |  | 2.42E-02 | 9.25E-03 | 1.62E-02 |
|  |  | A8HVJ9 | CHLREDRAFT_112806 | Photosystem II stability/assembly factor HCF136 | 0.24 | 0.10 | 0.13 |  | 1.47E-04 | 4.77E-06 | 2.84E-05 |
|  |  | A8J503 | PRPL6 | Plastid ribosomal protein L6 | 0.94 | 0.16 | 0.15 |  | 8.44E-01 | 3.37E-05 | 6.67E-05 |
|  |  | Q8HTL1 | rpl5 | 50S ribosomal protein L5 chloroplastic | 2.79 | 0.68 | 0.37 |  | 1.09E-02 | 6.66E-02 | 1.61E-03 |
|  |  | A8JAL6 | PRPL15 | Plastid ribosomal protein L15 | 0.24 | 0.04 | 0.05 |  | 3.39E-04 | 2.86E-07 | 2.65E-07 |
|  |  | A8J785 | ATPG | ATP synthase subunit b' chloroplastic | 0.90 | 1.00 | 1.21 |  | 2.95E-02 | 9.98E-01 | 2.05E-03 |
|  |  | A8JC21 | UROD1 | Uroporphyrinogen decarboxylase | 0.41 | 0.21 | 0.24 |  | 3.97E-03 | 3.99E-04 | 7.65E-03 |
|  |  | A8IYP4 | PRK1 | Phosphoribulokinase | 1.04 | 0.12 | 0.11 |  | 8.34E-01 | 2.66E-06 | 1.77E-09 |
|  |  | A8J8U1 | RPN10 | 26S proteasome regulatory subunit | 0.84 | 0.65 | 0.79 |  | 2.76E-01 | 1.31E-03 | 4.13E-03 |
|  |  | A8I8X2 | DEG1A | DegP-type protease | 1.40 | 1.14 | 0.37 |  | 3.26E-01 | 8.02E-01 | 3.93E-02 |
|  |  | A8IKQ0 | FBP1 | Fructose-1,6-bisphosphatase | 0.66 | 0.28 | 0.07 |  | 1.41E-05 | 1.39E-04 | 5.99E-08 |
|  |  | A8INR7 | PRPL27 | Plastid ribosomal protein L27 | 0.26 | 0.14 | 0.15 |  | 8.50E-04 | 8.24E-05 | 9.15E-05 |

Supporting information Table S3. List of differentially expressed proteins in *hpm91* delineated from Dataset S6 that represents major proteome changes during H_2_ production process.

| Ranking | GOPB | Uniprot accession | Gene ID | Protein name | Ratio (Mut/Mu0) | | |  | *p*-value | | |  |
| --- | --- | --- | --- | --- | --- | --- | --- | --- | --- | --- | --- | --- |
|  |  |  |  |  | 24 h | 72 h | 120 h |  | 24 h | 72 h | 120 h | |
| 1 | Translation | A8J576 | RPS27-A | 40S ribosomal protein S27 | 0.45 | 0.17 | 0.12 |  | 2.93E-02 | 7.18E-03 | 5.28E-03 | |
|  |  | A8J0V6 | RPS27-B | 40S ribosomal protein S27 | 2.19 | 2.93 | 2.10 |  | 6.83E-02 | 4.64E-02 | 8.00E-02 | |
|  |  | A8HVQ1 | RPS8 | 40S ribosomal protein S8 | 0.34 | 0.16 | 0.12 |  | 1.55E-03 | 2.76E-04 | 3.92E-04 | |
|  |  | A8IB25 | CHLREDRAFT_126059 | 40S ribosomal protein SA | 0.88 | 0.68 | 0.54 |  | 2.84E-01 | 3.35E-02 | 4.21E-02 | |
|  |  | A8IUV7 | RPL13 | 60S ribosomal protein L13 | 1.55 | 0.52 | 0.36 |  | 1.81E-01 | 9.02E-03 | 2.48E-03 | |
|  |  | A8IKZ2 | RPL18 | 60S ribosomal protein L18 | 1.18 | 0.57 | 0.32 |  | 5.55E-01 | 3.45E-02 | 7.44E-03 | |
|  |  | A8IQC1 | RPL27 | 60S ribosomal protein L27 | 6.40 | 3.47 | 3.09 |  | 2.04E-03 | 5.01E-02 | 5.34E-02 | |
|  |  | Q8GUQ9 | RPL38 | 60S ribosomal protein L38 | 3.92 | 1.77 | 1.91 |  | 1.10E-02 | 6.47E-04 | 1.43E-01 | |
|  |  | A8JCA8 | EFG5 | Elongation factor EF-Tu-like protein | 0.96 | 2.46 | 1.88 |  | 5.97E-01 | 5.96E-03 | 4.29E-02 | |
|  |  | A8JB67 | NHP2 | Nucleolar protein small subunit of H/ACA snoRNPs | 0.76 | 0.57 | 0.54 |  | 8.38E-02 | 3.16E-02 | 6.80E-02 | |
|  |  | A8J9X5 | CHLREDRAFT_18599 | Predicted protein | 0.85 | 0.58 | 0.58 |  | 3.29E-01 | 4.36E-02 | 1.43E-01 | |
|  |  | A8JHJ5 | CHLRE_15g641200v5 | Predicted protein | 0.14 | 0.14 | 0.12 |  | 1.87E-02 | 1.95E-02 | 1.67E-02 | |
|  |  | A8I9M5 | CHLREDRAFT_141578 | Predicted protein | 0.16 | 0.10 | 0.09 |  | 7.09E-04 | 1.28E-04 | 1.26E-04 | |
|  |  | A8I4T2 | RPL10a | Ribosomal protein | 10.16 | 9.34 | 7.90 |  | 7.41E-03 | 9.96E-03 | 1.64E-02 | |
|  |  | A8IZK3 | RPL10 | Ribosomal protein L10 | 0.69 | 0.41 | 0.21 |  | 2.71E-01 | 6.23E-02 | 2.27E-02 | |
|  |  | A8HQ81 | RPL11 | Ribosomal protein L11 | 4.01 | 1.94 | 1.52 |  | 4.15E-04 | 2.21E-03 | 4.46E-03 | |
|  |  | A8IA18 | RPL19 | Ribosomal protein L19 | 0.43 | 0.15 | 0.11 |  | 2.37E-02 | 6.66E-03 | 5.78E-03 | |
|  |  | A8J951 | RPL21 | Ribosomal protein L21 | 3.25 | 1.56 | 1.31 |  | 2.87E-02 | 2.27E-01 | 5.72E-01 | |
|  |  | A8J239 | RPL23a | Ribosomal protein L23a | 1.00 | 0.38 | 0.34 |  | 9.93E-01 | 1.74E-02 | 3.25E-02 | |
|  |  | A8I2T0 | RPL27a | Ribosomal protein L27a | 0.56 | 0.29 | 0.17 |  | 1.13E-01 | 2.20E-02 | 1.34E-02 | |
|  |  | A8ID84 | RPL3 | Ribosomal protein L3 | 0.35 | 0.28 | 0.13 |  | 2.44E-05 | 1.00E-05 | 3.36E-06 | |
|  |  | A8ILG8 | RPL31 | Ribosomal protein L31 | 0.80 | 0.25 | 0.23 |  | 6.12E-01 | 5.20E-03 | 3.38E-03 | |
|  |  | A8J2G4 | RPL32 | Ribosomal protein L32 | 0.85 | 0.45 | 0.25 |  | 6.17E-01 | 5.13E-02 | 6.51E-03 | |
|  |  | A8J8P4 | RPL34 | Ribosomal protein L34 | 0.42 | 0.35 | 0.28 |  | 1.62E-02 | 3.43E-03 | 3.90E-03 | |
|  |  | A8HNX3 | RPL35 | Ribosomal protein L35 | 10.24 | 4.02 | 3.79 |  | 7.92E-03 | 9.88E-02 | 3.01E-01 | |
|  |  | A8J0I0 | RPL4 | Ribosomal protein L4 | 0.55 | 0.33 | 0.20 |  | 8.05E-02 | 2.13E-02 | 1.34E-02 | |
|  |  | A8HP55 | RPL5 | Ribosomal protein L5 | 2.31 | 1.65 | 1.36 |  | 2.93E-02 | 8.88E-02 | 3.81E-01 | |
|  |  | A8J567 | RPL7a | Ribosomal protein L7a | 4.76 | 3.33 | 2.29 |  | 4.52E-02 | 7.26E-02 | 2.15E-01 | |
|  |  | A8JHC3 | RPS11 | Ribosomal protein S11 | 0.23 | 0.18 | 0.09 |  | 1.11E-02 | 8.20E-03 | 5.55E-03 | |
|  |  | A8JE07 | RPS15a | Ribosomal protein S15a | 2.94 | 1.35 | 0.96 |  | 1.46E-02 | 5.48E-01 | 6.01E-01 | |
|  |  | A8JGK1 | RPS17 | Ribosomal protein S17 | 8.26 | 4.47 | 3.14 |  | 2.23E-02 | 4.37E-02 | 1.08E-01 | |
|  |  | A8HVP2 | RPS18 | Ribosomal protein S18 | 7.16 | 5.10 | 4.23 |  | 1.90E-02 | 2.74E-02 | 1.05E-01 | |
|  |  | A8I403 | RPS19 | Ribosomal protein S19 | 9.38 | 4.81 | 3.55 |  | 2.78E-03 | 4.07E-03 | 5.75E-02 | |
|  |  | A8HME4 | RPS2 | Ribosomal protein S2 | 1.08 | 0.59 | 0.43 |  | 7.55E-01 | 2.69E-02 | 1.13E-02 | |
|  |  | A8J8M9 | RPS20 | Ribosomal protein S20 | 0.94 | 0.35 | 0.32 |  | 7.13E-01 | 2.02E-03 | 8.37E-03 | |
|  |  | A8IS22 | RPS26 | Ribosomal protein S26 | 0.68 | 0.35 | 0.25 |  | 3.12E-01 | 4.12E-02 | 2.10E-02 | |
|  |  | A8HVK4 | RPS27a | Ribosomal protein S27a | 0.23 | 0.16 | 0.12 |  | 2.02E-04 | 2.78E-04 | 1.06E-04 | |
|  |  | A8I4P5 | RPS3 | Ribosomal protein S3 | 0.92 | 0.42 | 0.36 |  | 8.37E-01 | 6.11E-02 | 4.83E-02 | |
|  |  | A8JF66 | RPS30 | Ribosomal protein S30 | 0.70 | 0.37 | 0.27 |  | 1.68E-01 | 1.78E-02 | 8.68E-03 | |
|  |  | A8IMP6 | RPS4 | Ribosomal protein S4 | 0.90 | 0.41 | 0.27 |  | 8.48E-01 | 4.79E-02 | 2.11E-02 | |
|  |  | A8J2I5 | RPS5 | Ribosomal protein S5 | 5.85 | 5.29 | 4.81 |  | 5.04E-02 | 1.87E-02 | 1.45E-01 | |
|  |  | A8JGF8 | RPS9 | Ribosomal protein S9 component of cytosolic 80S ribosome and 40S small subunit | 5.46 | 2.10 | 1.72 |  | 3.72E-02 | 9.80E-02 | 2.94E-01 | |
|  |  | A8J8J8 | CHLREDRAFT_120661 | GTPase Der (Fragment) | 0.47 | 0.48 | 0.32 |  | 3.12E-02 | 3.46E-02 | 1.62E-02 | |
|  |  | A8I2G3 | CHLREDRAFT_112251 | Predicted protein (Fragment) | 0.53 | 0.53 | 0.48 |  | 2.58E-03 | 2.01E-02 | 3.95E-03 | |
|  |  | A8JHU2 | RPL36 | 60S ribosomal protein L36 | 7.38 | 2.61 | 2.37 |  | 2.91E-02 | 1.14E-01 | 1.88E-01 | |
|  |  | A8I982 | RPL15 | Ribosomal protein L15 | 0.84 | 0.53 | 0.29 |  | 7.78E-01 | 1.28E-01 | 3.72E-02 | |
|  |  | A8JI94 | RPL22 | Ribosomal protein L22 | 20.81 | 11.42 | 10.69 |  | 5.90E-03 | 1.56E-02 | 1.23E-02 | |
|  |  | A8HMG7 | RPL26 | Ribosomal protein L26 | 1.45 | 0.58 | 0.42 |  | 1.27E-01 | 4.75E-02 | 1.87E-02 | |
|  |  | A8I0Y2 | RPL35a | Ribosomal protein L35a | 1.14 | 0.42 | 0.32 |  | 6.68E-01 | 5.45E-02 | 2.44E-02 | |
|  |  | A8IVE2 | RPL7 | Ribosomal protein L7 | 5.18 | 3.42 | 2.59 |  | 1.37E-03 | 4.42E-03 | 3.59E-02 | |
|  |  | A8IVK1 | RPL8 | Ribosomal protein L8 | 0.32 | 0.20 | 0.20 |  | 1.66E-02 | 9.03E-03 | 1.11E-02 | |
|  |  | Q7YKX3 | rps11 | 30S ribosomal protein S11 chloroplastic | 0.45 | 0.21 | 0.25 |  | 4.65E-02 | 1.23E-02 | 1.45E-02 | |
|  |  | P59776 | rps19 | 30S ribosomal protein S19 chloroplastic | 0.27 | 0.07 | 0.07 |  | 3.50E-03 | 2.63E-04 | 2.61E-04 | |
|  |  | O47027 | rps2-1 | 30S ribosomal protein S2 chloroplastic | 0.26 | 0.18 | 0.18 |  | 1.01E-02 | 8.05E-03 | 7.46E-03 | |
|  |  | Q08365 | rps3 | 30S ribosomal protein S3 chloroplastic | 0.23 | 0.08 | 0.08 |  | 2.07E-02 | 1.01E-02 | 9.66E-03 | |
|  |  | P48270 | rps4 | 30S ribosomal protein S4 chloroplastic | 0.66 | 0.26 | 0.27 |  | 1.09E-02 | 1.74E-03 | 3.97E-04 | |
|  |  | P48267 | rps7 | 30S ribosomal protein S7 chloroplastic | 2.65 | 1.47 | 1.71 |  | 2.50E-03 | 1.12E-01 | 2.17E-02 | |
|  |  | O20029 | rps9 | 30S ribosomal protein S9 chloroplastic | 0.23 | 0.09 | 0.10 |  | 1.92E-03 | 9.68E-04 | 9.97E-04 | |
|  |  | Q8HTL2 | rpl2 | 50S ribosomal protein L2 chloroplastic | 0.22 | 0.06 | 0.05 |  | 1.86E-03 | 8.92E-04 | 8.51E-04 | |
|  |  | P26565 | rpl20 | 50S ribosomal protein L20 chloroplastic | 1.28 | 0.47 | 0.26 |  | 3.53E-01 | 2.83E-02 | 1.18E-02 | |
|  |  | Q8HTL3 | rpl23 | 50S ribosomal protein L23 chloroplastic | 0.64 | 0.16 | 0.13 |  | 1.51E-01 | 1.56E-02 | 1.59E-02 | |
|  |  | A8JEP1 | PRPL35 | 50S ribosomal protein L35 | 0.91 | 0.35 | 0.28 |  | 8.87E-01 | 3.70E-02 | 7.66E-02 | |
|  |  | Q8HTL1 | rpl5 | 50S ribosomal protein L5 chloroplastic | 1.33 | 0.64 | 0.52 |  | 2.64E-01 | 6.14E-03 | 5.84E-04 | |
|  |  | A8I8Z4 | PRPL1 | Plastid ribosomal protein L1 | 0.41 | 0.08 | 0.07 |  | 5.36E-03 | 1.22E-03 | 1.04E-03 | |
|  |  | A8ICE4 | PRPL11 | Plastid ribosomal protein L11 | 0.86 | 0.40 | 0.36 |  | 7.23E-01 | 6.94E-03 | 4.67E-03 | |
|  |  | A8HWZ6 | PRPL13 | Plastid ribosomal protein L13 | 0.43 | 0.12 | 0.15 |  | 3.68E-02 | 1.08E-02 | 1.08E-02 | |
|  |  | A8JAL6 | PRPL15 | Plastid ribosomal protein L15 | 0.33 | 0.05 | 0.05 |  | 2.04E-02 | 5.06E-03 | 5.00E-03 | |
|  |  | A8I3M4 | PRPL17 | Plastid ribosomal protein L17 | 1.41 | 0.57 | 0.52 |  | 2.03E-01 | 5.56E-02 | 3.12E-02 | |
|  |  | A8HNJ8 | PRPL18 | Plastid ribosomal protein L18 | 0.82 | 0.17 | 0.12 |  | 4.36E-01 | 1.99E-02 | 1.55E-02 | |
|  |  | A8IW44 | PRPL19 | Plastid ribosomal protein L19 | 0.33 | 0.11 | 0.13 |  | 2.24E-02 | 6.37E-03 | 6.79E-03 | |
|  |  | A8J9D9 | PRPL24 | Plastid ribosomal protein L24 | 1.06 | 0.21 | 0.28 |  | 8.00E-01 | 2.37E-04 | 7.63E-03 | |
|  |  | A8INR7 | PRPL27 | Plastid ribosomal protein L27 | 0.19 | 0.05 | 0.04 |  | 2.00E-03 | 8.47E-04 | 7.99E-04 | |
|  |  | A8HWS8 | PRPL28 | Plastid ribosomal protein L28 | 0.23 | 0.06 | 0.04 |  | 5.36E-03 | 2.14E-03 | 1.96E-03 | |
|  |  | A8JE35 | PRPL3 | Plastid ribosomal protein L3 | 0.28 | 0.07 | 0.05 |  | 1.78E-03 | 5.59E-04 | 4.50E-04 | |
|  |  | A8IUC3 | PRPL32 | Plastid ribosomal protein L32 | 2.07 | 0.40 | 0.38 |  | 1.06E-01 | 1.19E-02 | 1.11E-02 | |
|  |  | A8I1D3 | PRPL33 | Plastid ribosomal protein L33 | 1.24 | 0.55 | 0.39 |  | 3.48E-01 | 2.82E-02 | 2.34E-02 | |
|  |  | A8J503 | PRPL6 | Plastid ribosomal protein L6 | 0.79 | 0.20 | 0.15 |  | 1.58E-01 | 3.33E-03 | 2.03E-03 | |
|  |  | A8IYS1 | PRPL9 | Plastid ribosomal protein L9 | 0.80 | 0.23 | 0.18 |  | 5.23E-01 | 4.80E-02 | 4.01E-02 | |
|  |  | A8JGS2 | PRPS17 | Plastid ribosomal protein S17 | 0.24 | 0.08 | 0.08 |  | 1.41E-03 | 9.29E-05 | 9.46E-05 | |
|  |  | A8JDN4 | PRPS20 | Plastid ribosomal protein S20 | 2.80 | 1.28 | 1.39 |  | 9.34E-04 | 2.28E-01 | 2.27E-01 | |
|  |  | A8J8M5 | PRPS5 | Plastid ribosomal protein S5 | 0.16 | 0.04 | 0.06 |  | 1.85E-04 | 8.32E-05 | 8.88E-05 | |
|  |  | A8IMN3 | PSRP-6 | Plastid-specific ribosomal protein 6 | 0.11 | 0.04 | 0.04 |  | 1.77E-04 | 6.56E-05 | 7.10E-05 | |
|  |  | A8IA39 | EFG1 | Chloroplast elongation factor G | 0.42 | 0.48 | 0.52 |  | 1.49E-02 | 2.51E-03 | 4.08E-02 | |
|  |  | A8IPJ0 | MITC11 | Mitochondrial carrier protein | 1.74 | 1.25 | 1.28 |  | 3.06E-02 | 4.37E-01 | 5.06E-01 | |
|  |  | A8IZL2 | MRPL21 | Mitochondrial ribosomal protein L21 | 1.00 | 0.80 | 1.02 |  | 9.29E-01 | 1.25E-02 | 7.37E-01 | |
|  |  | A8HXM1 | MRPL29 | Mitochondrial ribosomal protein L29 | 11.63 | 4.41 | 3.79 |  | 3.95E-03 | 2.66E-02 | 1.72E-01 | |
|  |  | A8JFK9 | MRPL7/L12 | Mitochondrial ribosomal protein L7/L12 | 2.95 | 3.97 | 3.80 |  | 5.64E-02 | 1.93E-02 | 3.47E-02 | |
|  |  | A8HXY9 | UCP2 | Uncoupling protein | 3.24 | 3.56 | 2.82 |  | 1.54E-02 | 3.15E-03 | 7.65E-02 | |
|  |  | A8JCF4 | MRPS17 | Mitochondrial ribosomal protein S17 | 0.56 | 0.27 | 0.59 |  | 1.29E-02 | 3.47E-03 | 9.44E-03 | |
|  |  | A8J2A5 | MSCP1 | Mitochondrial substrate carrier protein | 0.91 | 0.34 | 0.41 |  | 6.79E-01 | 4.78E-03 | 1.05E-02 | |
| 2 | Protein folding | A8J524 | CCT2 | T-complex protein 1 beta subunit | 1.72 | 2.10 | 1.52 |  | 1.85E-02 | 7.82E-03 | 3.03E-02 | |
|  |  | A8II42 | CCT3 | T-complex protein 1 gamma subunit | 1.05 | 2.02 | 1.32 |  | 9.96E-01 | 1.88E-02 | 3.01E-01 | |
|  |  | A8JE04 | CCT4 | T-complex protein 1 subunit delta | 1.49 | 3.08 | 1.98 |  | 2.59E-01 | 8.85E-03 | 2.13E-02 | |
|  |  | A8J7J2 | CCT5 | T-complex protein epsilon subunit | 1.34 | 3.71 | 3.17 |  | 4.79E-01 | 2.42E-02 | 1.48E-01 | |
|  |  | A8J014 | CCT6 | T-complex protein zeta subunit | 3.00 | 3.29 | 2.50 |  | 3.38E-02 | 1.77E-03 | 2.35E-02 | |
|  |  | A8HQ74 | CCT7 | T-complex protein eta subunit | 0.89 | 2.87 | 2.40 |  | 4.60E-01 | 9.45E-03 | 7.80E-02 | |
|  |  | A8IUG8 | CDSP32 | Plastidic thioredoxin-like protein | 1.02 | 0.26 | 0.42 |  | 9.20E-01 | 9.08E-03 | 2.25E-02 | |
|  |  | A8HMC0 | CRT2 | Calreticulin 2 calcium-binding protein | 1.46 | 0.54 | 0.20 |  | 2.61E-01 | 6.07E-02 | 1.26E-02 | |
|  |  | A8IV02 | CYN1a\|CYN1b | Peptidyl-prolyl cis-transisomerase cyclophilin type | 0.86 | 0.82 | 0.65 |  | 4.09E-01 | 1.33E-01 | 1.60E-02 | |
|  |  | A8JD64 | CYN19-2 | Peptidyl-prolyl cis-trans isomerase | 0.70 | 0.60 | 0.45 |  | 2.07E-01 | 1.16E-01 | 4.91E-02 | |
|  |  | A8J282 | CYN20-1 | Peptidyl-prolyl cis-trans isomerase | 4.78 | 7.32 | 2.43 |  | 1.27E-03 | 4.11E-03 | 2.37E-01 | |
|  |  | A8JDL5 | CYN20-3 | Peptidyl-prolyl cis-trans isomerase | 0.79 | 0.44 | 0.31 |  | 5.31E-01 | 7.53E-02 | 3.44E-02 | |
|  |  | A8ID98 | CYN20-5 | Peptidyl-prolyl cis-trans isomerase cyclophilin-type | 0.70 | 0.50 | 0.58 |  | 1.64E-01 | 6.48E-03 | 2.10E-02 | |
|  |  | A8IE53 | CYN26 | Peptidyl-prolyl cis-trans isomerase cyclophilin-type | 1.07 | 0.29 | 0.44 |  | 8.49E-01 | 1.76E-02 | 3.66E-02 | |
|  |  | A8JHN8 | CYN28 | Peptidyl-prolyl cis-trans isomerase cyclophilin-type | 1.03 | 0.32 | 0.57 |  | 6.74E-01 | 2.96E-02 | 1.16E-01 | |
|  |  | A8HN48 | CYN40 | Peptidyl-prolyl cis-trans isomerase cyclophilin-type | 1.88 | 2.14 | 1.68 |  | 2.78E-02 | 1.07E-01 | 1.93E-01 | |
|  |  | A8IQC5 | DNJ1 | DnaJ-like protein | 0.47 | 2.01 | 1.34 |  | 7.12E-02 | 2.98E-02 | 2.79E-01 | |
|  |  | A8I6Y0 | ERJ1 | ER DnaJ-like protein 1 | 0.80 | 0.69 | 0.76 |  | 3.08E-01 | 3.86E-02 | 3.77E-01 | |
|  |  | A8J3L6 | FKB16-2a/2c/2b | Peptidyl-prolyl cis-trans isomerase | 0.85 | 0.43 | 0.20 |  | 4.01E-01 | 6.75E-03 | 1.03E-02 | |
|  |  | A8J3C1 | FKB16-7a\|FKB16-7b | Peptidyl-prolyl cis-trans isomerase | 0.66 | 0.25 | 0.25 |  | 1.93E-01 | 1.58E-03 | 1.32E-03 | |
|  |  | Q944P3 | HSP33 | Heat shock protein 33 | 0.54 | 0.37 | 0.51 |  | 2.24E-02 | 7.48E-03 | 1.95E-02 | |
|  |  | A8J1U1 | HSP90A | Heat shock protein 90A | 3.89 | 5.74 | 3.69 |  | 5.61E-02 | 2.12E-02 | 4.93E-02 | |
|  |  | A8I7T1 | HSP90B | Heat shock protein 90B | 5.17 | 5.76 | 5.33 |  | 5.98E-03 | 1.24E-03 | 2.06E-03 | |
|  |  | A8JES1 | MGE1 | GrpE protein homolog | 5.54 | 2.70 | 3.45 |  | 8.02E-04 | 2.90E-03 | 1.06E-01 | |
|  |  | A8HPB8 | MSRA4 | Peptidyl-prolyl cis-trans isomerase | 0.69 | 0.45 | 0.38 |  | 1.26E-02 | 4.24E-03 | 3.33E-03 | |
|  |  | A8IHI1 | PDI4 | Protein disulfide isomerase | 0.93 | 2.49 | 2.33 |  | 4.36E-01 | 3.08E-03 | 1.02E-02 | |
|  |  | A8JD56 | TIG1 | Chloroplast trigger factor | 0.61 | 0.60 | 0.71 |  | 2.94E-02 | 5.08E-02 | 8.36E-02 | |
|  |  | A8IZR5 | TRXh | Thioredoxin | 9.04 | 19.31 | 18.47 |  | 5.35E-02 | 5.12E-02 | 3.58E-02 | |
|  |  | A8HP58 | TRXm | Thioredoxin | 1.05 | 0.60 | 0.35 |  | 8.14E-01 | 8.48E-02 | 3.57E-02 | |
|  |  | Q84XR9 | TRXx | Thioredoxin x | 4.04 | 5.26 | 5.91 |  | 3.51E-02 | 3.96E-02 | 7.57E-02 | |
|  |  | A8IQA9 | CHLREDRAFT_11164 | Thioredoxin-like protein | 0.97 | 0.67 | 0.55 |  | 9.94E-01 | 1.61E-01 | 7.22E-03 | |
|  |  | A8I0I4 | CHLRE_10g456250v5 | Thioredoxin-like protein | 0.58 | 0.36 | 0.32 |  | 3.72E-02 | 1.27E-02 | 1.38E-02 | |
|  |  | A8J0Q8 | CITRX | Thioredoxin-related protein CITRX | 3.06 | 3.95 | 3.60 |  | 9.08E-02 | 2.45E-02 | 3.16E-02 | |
|  |  | A8I211 | CHLREDRAFT_161043 | Predicted protein | 1.24 | 1.44 | 1.77 |  | 1.51E-01 | 4.69E-02 | 1.75E-03 | |
|  |  | A8HUK0 | FKB12 | Peptidyl-prolyl cis-trans isomerase | 0.64 | 0.20 | 0.07 |  | 3.65E-02 | 3.34E-04 | 6.21E-05 | |
|  |  | A8J746 | FKB15-1 | Peptidyl-prolyl cis-trans isomerase | 0.69 | 0.26 | 0.10 |  | 4.05E-02 | 5.50E-04 | 8.91E-05 | |
|  |  | A8I6B6 | FKB16-1 | Peptidyl-prolyl cis-trans isomerase | 0.98 | 0.42 | 0.16 |  | 9.32E-01 | 8.20E-03 | 2.20E-03 | |
|  |  | A8JC71 | FKB16-4 | Peptidyl-prolyl cis-trans isomerase | 0.61 | 0.22 | 0.24 |  | 1.93E-01 | 4.16E-02 | 6.74E-02 | |
|  |  | A8J3L3 | FKB16-5 | Peptidyl-prolyl cis-trans isomerase | 0.85 | 0.57 | 0.34 |  | 5.35E-01 | 6.94E-02 | 1.74E-02 | |
|  |  | A8JEI4 | FKB16-8 | Peptidyl-prolyl cis-trans isomerase | 0.97 | 0.32 | 0.34 |  | 6.73E-01 | 1.74E-03 | 1.18E-02 | |
|  |  | A8I0A8 | FKB16-9 | Peptidyl-prolyl cis-trans isomerase | 1.19 | 0.67 | 0.45 |  | 4.22E-01 | 1.15E-01 | 4.98E-02 | |
|  |  | A8JEK6 | FKB17-2 | Peptidyl-prolyl cis-trans isomerase | 0.72 | 0.74 | 0.84 |  | 1.78E-02 | 1.15E-01 | 4.11E-01 | |
|  |  | A8I1U5 | FKB18 | Peptidyl-prolyl cis-trans isomerase | 0.94 | 0.30 | 0.32 |  | 5.81E-01 | 2.82E-03 | 2.00E-03 | |
|  |  | A8IVN2 | FKB19 | Peptidyl-prolyl cis-trans isomerase | 4.07 | 1.99 | 1.55 |  | 1.65E-02 | 1.56E-01 | 3.62E-01 | |
| 3 | Carbon metabolism | A8J4D3 | AMYA2 | Alpha-amylase | 2.27 | 3.32 | 1.95 |  | 4.37E-02 | 3.93E-02 | 5.14E-02 | |
|  |  | A8IZ00 | CHLREDRAFT_173725 | Alpha-amylase-like protein | 5.64 | 2.54 | 2.24 |  | 4.09E-02 | 1.56E-01 | 3.05E-01 | |
|  |  | A8IZE7 | ATF1 | Glucosamine--fructose-6-phosphate aminotransferase | 0.69 | 2.58 | 2.32 |  | 3.12E-01 | 1.00E-02 | 4.49E-02 | |
|  |  | A8IEM7 | GHL1 | Glycosyl hydrolase | 1.44 | 3.06 | 2.39 |  | 9.72E-02 | 1.15E-01 | 3.72E-02 | |
|  |  | A8HPD2 | GPM2 | Phosphoglucomutase | 1.25 | 0.71 | 0.80 |  | 1.42E-01 | 2.96E-01 | 3.16E-02 | |
|  |  | A8ICG9 | MDH2 | Malate dehydrogenase | 0.37 | 0.94 | 0.34 |  | 3.21E-02 | 8.11E-01 | 3.41E-02 | |
|  |  | A8J0W9 | MDH3 | Malate dehydrogenase | 1.88 | 3.42 | 3.31 |  | 2.34E-01 | 4.50E-03 | 1.01E-02 | |
|  |  | A8JHU0 | MDH4 | Malate dehydrogenase | 4.62 | 5.22 | 4.72 |  | 1.10E-02 | 2.10E-02 | 2.64E-02 | |
|  |  | Q9FNS5 | NADP-mdh | NADP-Malate dehydrogenase | 0.32 | 0.16 | 0.45 |  | 2.27E-04 | 9.60E-05 | 5.58E-03 | |
|  |  | A8HQW1 | CHLREDRAFT_101528 | 6-phosphogluconolactonase-like protein | 8.14 | 7.27 | 8.51 |  | 1.14E-04 | 2.73E-03 | 2.98E-04 | |
|  |  | A8HMX2 | PFL1 | Pyruvate-formate lyase | 6.73 | 4.05 | 5.09 |  | 5.09E-04 | 2.39E-02 | 1.47E-02 | |
|  |  | A8IYK1 | PHOB | Phosphorylase | 4.07 | 5.96 | 5.03 |  | 9.69E-02 | 3.26E-02 | 4.11E-02 | |
|  |  | A8IYP4 | PRK1 | Phosphoribulokinase | 0.81 | 0.08 | 0.06 |  | 3.61E-01 | 2.68E-03 | 2.43E-03 | |
|  |  | A8HQP0 | TAL1 | Transaldolase | 1.32 | 0.61 | 0.75 |  | 4.53E-01 | 2.95E-02 | 4.71E-02 | |
|  |  | A8IUU3 | TAL2 | Transaldolase | 0.82 | 0.12 | 0.08 |  | 6.21E-01 | 1.93E-04 | 1.91E-04 | |
|  |  | A8J914 | UGD2 | UDP-glucose dehydrogenase | 0.93 | 1.77 | 1.04 |  | 8.81E-01 | 2.44E-02 | 9.99E-01 | |
|  |  | A8ITZ0 | CHLRE_07g347100v5 | Predicted protein | 1.11 | 2.11 | 1.79 |  | 9.35E-01 | 2.73E-02 | 1.66E-01 | |
|  |  | A8J352 | CHLREDRAFT_119219 | Predicted protein | 3.83 | 3.70 | 4.64 |  | 1.16E-02 | 4.62E-03 | 5.03E-03 | |
|  |  | A8ICN2 | CHLREDRAFT_96789 | Predicted protein | 3.88 | 6.13 | 6.10 |  | 3.71E-01 | 1.99E-02 | 2.77E-02 | |
|  |  | A8J3Q5 | CHLREDRAFT_80327 | Predicted protein | 0.59 | 0.51 | 0.34 |  | 3.03E-02 | 2.04E-02 | 8.86E-03 | |
|  |  | A8IXP6 | CHLRE_03g200650v5 | Predicted protein | 1.63 | 1.00 | 1.12 |  | 2.78E-02 | 9.63E-01 | 6.59E-01 | |
|  |  | A8HMQ1 | ACH1 | Aconitate hydratase | 1.79 | 3.77 | 3.24 |  | 3.56E-01 | 1.36E-02 | 8.69E-03 | |
|  |  | A8JHC9 | CIS1 | Citrate synthase | 12.56 | 16.14 | 15.51 |  | 6.88E-03 | 1.31E-03 | 1.02E-02 | |
|  |  | A8J2S0 | CIS2 | Citrate synthase | 0.54 | 0.42 | 0.27 |  | 5.60E-02 | 2.04E-02 | 5.23E-03 | |
|  |  | A8HX04 | SDH2 | Iron-sulfur subunit of mitochondrial succinate dehydrogenase | 23.15 | 37.75 | 34.39 |  | 1.02E-01 | 9.14E-03 | 5.02E-02 | |
|  |  | A8J9S7 | IDH3 | Isocitrate dehydrogenase [NADP] | 5.16 | 7.19 | 5.65 |  | 1.50E-02 | 6.04E-04 | 4.02E-03 | |
|  |  | A8J6V1 | IDH1 | Isocitrate dehydrogenase NAD-dependent | 1.19 | 1.61 | 1.74 |  | 4.73E-01 | 1.31E-01 | 3.97E-02 | |
|  |  | Q6X898 | MAS1 | Malate synthase | 0.58 | 0.14 | 0.10 |  | 8.07E-02 | 1.28E-02 | 1.15E-02 | |
|  |  | A8HP06 | SDH1 | Succinate dehydrogenase subunit A | 1.70 | 2.45 | 1.81 |  | 1.83E-01 | 2.71E-02 | 1.02E-01 | |
|  |  | A8HPU1 | SDH4 | Succinate dehydrogenase subunit D | 1.02 | 0.68 | 0.41 |  | 8.02E-01 | 1.16E-01 | 3.72E-02 | |
|  |  | A8J244 | ICL1 | Isocitrate lyase | 0.78 | 0.24 | 0.11 |  | 6.10E-01 | 6.01E-02 | 3.41E-02 | |
|  |  | A8IXQ5 | CHLRE_03g200250v5 | Predicted protein | 0.64 | 0.14 | 0.19 |  | 3.82E-02 | 1.36E-03 | 1.45E-03 | |
|  |  | A8J2R7 | CHLREDRAFT_104431 | Predicted protein (Fragment) | 2.03 | 5.22 | 7.63 |  | 1.99E-01 | 2.51E-02 | 8.56E-03 | |
|  |  | A8IKQ0 | FBP1 | Fructose-1,6-bisphosphatase | 1.11 | 0.52 | 0.35 |  | 6.64E-01 | 1.86E-02 | 6.54E-03 | |
|  |  | A8IE23 | PGI1 | Glucose-6-phosphate isomerase | 4.77 | 6.51 | 5.53 |  | 6.61E-03 | 8.84E-03 | 1.64E-02 | |
|  |  | A8J0N7 | PCK1b\|PCK1a | Phosphoenolpyruvate carboxykinase splice variant | 1.28 | 0.46 | 0.33 |  | 4.80E-01 | 7.80E-02 | 2.54E-02 | |
|  |  | A8JCE1 | CHLREDRAFT_121619 | Predicted protein (Fragment) | 0.14 | 0.13 | 0.19 |  | 1.88E-02 | 1.70E-02 | 2.24E-02 | |
|  |  | A8IRK4 | SEBP1 | Sedoheptulose-1,7-bisphosphatase | 0.42 | 0.13 | 0.12 |  | 3.94E-02 | 1.07E-02 | 1.37E-02 | |
|  |  | A8HX70 | PFK1 | Phosphofructokinase family protein | 3.05 | 3.05 | 2.55 |  | 5.83E-03 | 3.03E-04 | 1.02E-01 | |
|  |  | A8IYM0 | PFK2 | Phosphofructokinase family protein | 2.01 | 3.14 | 3.29 |  | 2.08E-03 | 7.28E-04 | 1.35E-03 | |
| 4 | Photosynthesis | P22666 | psbH | Photosystem II reaction center protein H | 30.03 | 25.79 | 19.68 |  | 4.75E-02 | 1.59E-02 | 1.09E-03 | |
|  |  | A8J0E4 | PsbO | Oxygen-evolving enhancer protein 1 of photosystem II | 0.99 | 0.82 | 0.78 |  | 8.87E-01 | 6.22E-05 | 2.81E-05 | |
|  |  | A8IXU9 | CGL30/PsbP | Photosystem II thylakoid lumenal 29.8 kDa protein PsbP | 16.11 | 14.48 | 16.24 |  | 5.36E-02 | 1.46E-02 | 3.91E-02 | |
|  |  | A8J3S9 | PsbP2 | PsbP-like protein | 1.01 | 0.46 | 0.54 |  | 8.96E-01 | 8.70E-02 | 2.53E-02 | |
|  |  | A8JEV1 | PsbQ | Oxygen evolving enhancer protein 3 | 21.52 | 27.63 | 25.07 |  | 7.66E-02 | 1.15E-03 | 2.14E-02 | |
|  |  | A8JFQ7 | PsbW | Photosystem II reaction center W protein | 0.21 | 0.07 | 0.09 |  | 3.68E-02 | 1.82E-02 | 1.98E-02 | |
|  |  | A8III5 | Psb28 | Photosystem II reaction center psb28 protein | 0.71 | 0.18 | 0.13 |  | 1.65E-01 | 1.48E-02 | 1.24E-02 | |
|  |  | P12154 | psaA | Photosystem I P700 chlorophyll a apoprotein A1 | 0.89 | 4.42 | 4.04 |  | 4.87E-01 | 2.83E-02 | 3.94E-02 | |
|  |  | P09144 | psaB | Photosystem I P700 chlorophyll a apoprotein A2 | 1.94 | 11.43 | 8.18 |  | 7.64E-01 | 1.34E-02 | 1.04E-01 | |
|  |  | Q5NKW4 | PsaD | Photosystem I reaction center subunit II 20 kDa | 13.21 | 20.61 | 19.15 |  | 3.30E-02 | 2.01E-03 | 3.28E-03 | |
|  |  | A8J4S1 | PsaF | Photosystem I reaction center subunit III | 7.69 | 1.36 | 1.71 |  | 4.46E-02 | 6.01E-01 | 5.47E-01 | |
|  |  | A8JHN9 | PsaG | Photosystem I reaction center subunit V | 0.42 | 0.06 | 0.05 |  | 5.62E-04 | 4.85E-06 | 8.48E-06 | |
|  |  | A8J6K8 | PsaK | Photosystem I reaction center subunit psaK | 0.77 | 0.29 | 0.27 |  | 1.24E-01 | 3.78E-03 | 5.12E-03 | |
|  |  | A8IL32 | PsaL | Photosystem I reaction center subunit XI | 0.87 | 0.34 | 0.49 |  | 7.35E-01 | 1.38E-02 | 2.82E-02 | |
|  |  | A8I835 | PsaN | Photosystem I reaction center subunit N | 0.82 | 0.05 | 0.05 |  | 3.29E-01 | 2.39E-05 | 3.02E-05 | |
|  |  | O20030 | ycf4 | Photosystem I assembly protein Ycf4 | 0.28 | 0.25 | 0.20 |  | 1.14E-04 | 6.28E-04 | 1.77E-04 | |
|  |  | P23577 | petA | Apocytochrome f | 2.90 | 0.33 | 0.22 |  | 2.11E-02 | 1.06E-01 | 7.28E-02 | |
|  |  | Q00471 | petB | Cytochrome b6 | 0.57 | 0.24 | 0.31 |  | 1.30E-01 | 2.59E-02 | 2.86E-02 | |
|  |  | A8HQJ5 | TEF30 | Predicted protein | 1.17 | 0.42 | 0.13 |  | 9.81E-01 | 1.05E-01 | 3.12E-02 | |
|  |  | A8I531 | CHLD | Magnesium chelatase subunit D | 0.18 | 0.14 | 0.19 |  | 7.89E-04 | 4.69E-04 | 6.84E-04 | |
|  |  | A8IMZ5 | CHLI1 | Magnesium chelatase subunit I | 0.13 | 0.08 | 0.15 |  | 4.50E-04 | 2.82E-04 | 4.74E-04 | |
|  |  | A8IKQ6 | CHLI2 | Magnesium chelatase subunit I | 0.26 | 0.11 | 0.25 |  | 2.07E-03 | 1.02E-03 | 3.65E-03 | |
|  |  | A8HPJ2 | POR | Light-dependent protochlorophyllide reductase | 0.52 | 0.06 | 0.14 |  | 4.33E-02 | 5.12E-03 | 7.21E-03 | |
|  |  | Q93WL4 | LHCBM3 | Light-harvesting chlorophyll-a/b binding protein LhcII-1.3 | 2.33 | 0.73 | 0.38 |  | 5.49E-02 | 2.12E-01 | 3.91E-02 | |
|  |  | Q9ZSJ4 | LHCBM5 | Chlorophyll a-b binding protein of LHCII | 12.89 | 4.40 | 2.80 |  | 1.08E-02 | 7.89E-02 | 3.48E-02 | |
|  |  | A8J287 | LHCBM6 | Chloropyll a-b binding protein of LHCII type I chloroplast | 0.97 | 0.35 | 0.13 |  | 8.54E-01 | 2.03E-02 | 5.18E-03 | |
|  |  | A8J270 | LHCBM8 | Chlorophyll a-b binding protein of LHCII | 0.78 | 0.29 | 0.25 |  | 6.71E-01 | 5.77E-02 | 4.29E-02 | |
|  |  | Q8S3T9 | LHCBM9 | Chlorophyll a-b binding protein of LHCII | 36.48 | 27.70 | 26.74 |  | 5.89E-03 | 1.47E-03 | 5.01E-03 | |
|  |  | A8J249 | LHCA1 | Light-harvesting protein of photosystem I | 2.32 | 0.73 | 0.29 |  | 2.43E-01 | 2.66E-01 | 2.20E-03 | |
|  |  | A8IKC8 | LHCA2 | Light-harvesting protein of photosystem I | 1.52 | 0.21 | 0.80 |  | 2.70E-01 | 7.76E-03 | 2.33E-01 | |
|  |  | Q75VY8 | LHCA5 | Light-harvesting chlorophyll-a/b protein of photosystem I | 0.78 | 0.29 | 0.40 |  | 3.76E-01 | 5.76E-05 | 4.28E-04 | |
|  |  | Q75VY6 | LHCA6 | Light-harvesting chlorophyll-a/b protein of photosystem I | 0.90 | 0.25 | 0.35 |  | 5.06E-01 | 6.28E-03 | 2.85E-02 | |
|  |  | A8ISG0 | LHCA7 | Light-harvesting protein of photosystem I | 0.87 | 0.23 | 0.21 |  | 4.58E-01 | 4.93E-03 | 3.44E-03 | |
|  |  | A8ITV3 | LHCA9 | Light-harvesting protein of photosystem I | 0.75 | 0.13 | 0.13 |  | 2.34E-01 | 8.53E-05 | 1.51E-04 | |
|  |  | A8J0A7 | ELI3 | Early light-inducible protein | 1.34 | 2.21 | 2.22 |  | 2.45E-01 | 3.93E-02 | 1.32E-01 | |
| 5 | Redox homeostasis | A8HNQ7 | NTRC1 | Thioredoxin reductase | 0.49 | 0.34 | 0.36 |  | 1.71E-03 | 1.50E-04 | 2.40E-03 | |
|  |  | A8IUG8 | CDSP32 | Plastidic thioredoxin-like protein | 1.02 | 0.26 | 0.42 |  | 9.20E-01 | 9.08E-03 | 2.25E-02 | |
|  |  | Q9FE86 | PRX1 | 2-cys peroxiredoxin chloroplastic | 16.83 | 17.62 | 16.67 |  | 1.14E-03 | 9.71E-05 | 7.07E-04 | |
|  |  | A8J0Q8 | CITRX | Thioredoxin-related protein CITRX | 3.06 | 3.95 | 3.60 |  | 9.08E-02 | 2.45E-02 | 3.16E-02 | |
|  |  | A8JDC5 | DLC5 | Flagellar outer arm dynein 14 kDa light chain LC5 | 0.44 | 0.40 | 0.44 |  | 4.73E-02 | 8.59E-03 | 2.69E-02 | |
|  |  | A8HPL8 | DLD2 | Dihydrolipoamide dehydrogenase | 0.72 | 0.31 | 0.20 |  | 2.82E-01 | 2.62E-02 | 2.06E-02 | |
|  |  | A8J6A7 | MET16/APR1 | Adenylylphosphosulfate reductase | 8.56 | 14.80 | 15.71 |  | 1.84E-03 | 1.70E-04 | 8.61E-04 | |
|  |  | A8J1T4 | GCSL | Dihydrolipoyl dehydrogenase | 2.95 | 5.47 | 5.00 |  | 1.98E-01 | 5.53E-02 | 4.37E-02 | |
|  |  | A8JHA9 | GRX1 | Glutaredoxin CPYC type | 2.76 | 3.31 | 3.57 |  | 1.98E-02 | 2.71E-04 | 1.06E-04 | |
|  |  | A8JH05 | GRX3 | Glutaredoxin CGFS type | 0.73 | 0.45 | 0.48 |  | 4.94E-02 | 1.18E-02 | 1.09E-02 | |
|  |  | A8HN52 | GRX6 | Glutaredoxin CGFS type | 0.46 | 0.22 | 0.24 |  | 3.50E-02 | 1.27E-03 | 3.54E-03 | |
|  |  | A8J0E5 | GSHR2 | Glutathione reductase | 1.24 | 2.16 | 2.08 |  | 2.91E-01 | 6.69E-03 | 2.01E-02 | |
|  |  | A8J448 | NTR1 | NADPH-dependent thioredoxin reductase 1 | 0.98 | 1.64 | 1.22 |  | 9.47E-01 | 4.73E-02 | 6.37E-01 | |
|  |  | A8HQT1 | PDI2 | Protein disulfide isomerase | 10.61 | 10.09 | 8.47 |  | 4.38E-03 | 1.98E-02 | 3.96E-02 | |
|  |  | A8IHI1 | PDI4 | Protein disulfide isomerase | 0.93 | 2.49 | 2.33 |  | 4.36E-01 | 3.08E-03 | 1.02E-02 | |
|  |  | A8I4C2 | PDI5 | Protein disulfide isomerase | 0.65 | 0.58 | 0.34 |  | 1.11E-02 | 1.76E-01 | 1.64E-02 | |
|  |  | A8HZQ4 | PRX3 | Peroxiredoxin type II | 0.83 | 0.57 | 0.36 |  | 1.69E-01 | 9.36E-03 | 1.34E-03 | |
|  |  | A8JFC3 | SCO1 | Cytochrome c oxidase assembly factor | 3.13 | 4.09 | 4.95 |  | 4.26E-02 | 1.62E-02 | 2.05E-02 | |
|  |  | A8IZR5 | TRXh | Thioredoxin | 9.04 | 19.31 | 18.47 |  | 5.35E-02 | 5.12E-02 | 3.58E-02 | |
|  |  | A8HP58 | TRXm | Thioredoxin | 1.05 | 0.60 | 0.35 |  | 8.14E-01 | 8.48E-02 | 3.57E-02 | |
|  |  | Q84XR9 | TRXx | Thioredoxin x | 4.04 | 5.26 | 5.91 |  | 3.51E-02 | 3.96E-02 | 7.57E-02 | |
|  |  | A8IXH4 | CHLREDRAFT_205510 | EF-Hand domain-containing thioredoxin | 6.82 | 6.24 | 5.33 |  | 3.63E-02 | 3.42E-02 | 1.77E-02 | |
|  |  | A8I0I4 | CHLRE_10g456250v5 | Thioredoxin-like protein | 0.58 | 0.36 | 0.32 |  | 3.72E-02 | 1.27E-02 | 1.38E-02 | |
|  |  | A8IQA9 | CHLREDRAFT_11164 | Thioredoxin-like protein | 0.97 | 0.67 | 0.55 |  | 9.94E-01 | 1.61E-01 | 7.22E-03 | |
|  |  | A8JG35 | CHLREDRAFT_160132 | Predicted protein | 0.95 | 0.52 | 0.48 |  | 5.06E-01 | 5.21E-02 | 3.39E-02 | |
|  |  | A8JDA2 | CHLRE_17g715500v5 | Predicted protein | 2.43 | 1.31 | 1.08 |  | 2.89E-03 | 3.49E-01 | 9.41E-01 | |
|  |  | A8J0Q4 | CHLREDRAFT_191208 | Predicted protein | 2.38 | 4.20 | 3.65 |  | 4.62E-01 | 4.41E-02 | 1.22E-01 | |
|  |  | A8JA70 | CHLRE_16g687294v5 | Ferredoxin thioredoxin reductase variable chain | 1.98 | 0.68 | 0.33 |  | 2.49E-02 | 1.18E-01 | 1.94E-02 | |
|  |  | O49822 | apx1 | Ascorbate peroxidase | 7.53 | 11.12 | 13.11 |  | 1.21E-02 | 3.31E-02 | 7.36E-03 | |
|  |  | A8J7X9 | CCPR1 | Cytochrome c peroxidase | 12.06 | 12.80 | 10.92 |  | 5.09E-05 | 5.23E-03 | 7.00E-03 | |
|  |  | A8J285 | APX2 | L-ascorbate peroxidase | 3.09 | 3.46 | 4.49 |  | 3.05E-02 | 4.10E-02 | 4.71E-02 | |
|  |  | O81648 | Lci2 | Low CO2 inducible gene | 0.79 | 0.07 | 0.16 |  | 3.32E-01 | 1.87E-04 | 6.86E-04 | |
|  |  | A8J537 | CAT1 | Catalase | 3.13 | 4.92 | 4.37 |  | 5.83E-02 | 1.17E-03 | 4.88E-05 | |
|  |  | A8IXD6 | CLPR4 | ATP-dependent Clp protease proteolytic subunit | 6.84 | 5.82 | 5.04 |  | 2.06E-02 | 4.72E-02 | 1.29E-01 | |
| 6 | Intracellular protein trafficking | A8I1Y7 | SNAPA1 | Alpha-SNAP | 0.94 | 3.31 | 2.91 |  | 9.51E-01 | 3.73E-02 | 8.47E-02 | |
|  |  | A8I4S9 | CHC1 | Clathrin heavy chain | 2.60 | 16.32 | 14.00 |  | 6.11E-01 | 1.47E-02 | 1.78E-02 | |
|  |  | A8HQF0 | VPS35 | Subunit of retromer complex | 2.77 | 3.49 | 3.31 |  | 1.04E-02 | 3.36E-03 | 9.94E-04 | |
|  |  | A8IM71 | COPG1 | Coatomer subunit gamma | 1.22 | 4.00 | 2.82 |  | 8.46E-01 | 4.12E-02 | 6.82E-02 | |
|  |  | A8J4X1 | VPS26 | Subunit of retromer complex | 6.42 | 4.86 | 4.91 |  | 6.47E-04 | 1.25E-02 | 1.16E-03 | |
|  |  | A8INI9 | CHLREDRAFT_170116 | Predicted protein | 1.36 | 0.86 | 1.11 |  | 3.53E-02 | 2.79E-01 | 7.56E-01 | |
|  |  | A8JEP9 | COPB1 | Coatomer subunit beta | 1.01 | 3.93 | 3.32 |  | 9.99E-01 | 3.69E-02 | 5.48E-02 | |
|  |  | A8I211 | CHLREDRAFT_161043 | Predicted protein | 1.24 | 1.44 | 1.77 |  | 1.51E-01 | 4.69E-02 | 1.75E-03 | |
|  |  | Q8S4W5 | SYP6 | Qc-SNARE protein Tlg1/Syntaxin 6-family | 2.82 | 2.89 | 2.98 |  | 4.07E-02 | 1.65E-02 | 4.07E-03 | |
|  |  | A8J729 | AP1B1 | Putative uncharacterized protein AP1B1 | 2.70 | 5.15 | 4.06 |  | 5.51E-02 | 2.56E-04 | 1.34E-03 | |
|  |  | A8IR64 | SYP5 | Qc-SNARE protein Syn8/Syntaxin8-family | 4.09 | 5.61 | 5.62 |  | 6.63E-02 | 8.15E-02 | 4.75E-02 | |
|  |  | A8JBG7 | SYP3 | Qa-SNARE protein Sed5/Syntaxin5-family | 1.58 | 1.16 | 0.71 |  | 9.51E-02 | 6.30E-01 | 3.55E-02 | |
|  |  | A8JC30 | SAR1 | Sar-type small GTPase | 3.05 | 4.19 | 3.02 |  | 7.23E-02 | 2.70E-02 | 5.48E-02 | |
|  |  | A8JGS8 | COPB2 | Beta'-cop | 1.56 | 3.25 | 1.91 |  | 7.90E-02 | 4.00E-03 | 1.49E-02 | |
|  |  | A8IMC5 | AP4E1 | Epsilon-adaptin | 0.83 | 2.44 | 2.23 |  | 2.81E-01 | 2.20E-02 | 1.18E-02 | |
|  |  | A8J7K1 | CLC1 | Clathrin light chain | 2.90 | 2.62 | 1.96 |  | 2.37E-02 | 7.70E-02 | 1.31E-01 | |
|  |  | A8JCA4 | CHLREDRAFT_195581 | RabGAP/TBC protein | 5.94 | 12.65 | 9.92 |  | 3.04E-02 | 3.90E-03 | 1.97E-03 | |
|  |  | A8IYJ6 | CGL38 | Predicted protein | 1.47 | 0.20 | 0.21 |  | 5.06E-01 | 1.81E-02 | 1.01E-02 | |
|  |  | A8HXA2 | AP1M1 | Mu1-Adaptin | 1.89 | 2.95 | 2.66 |  | 2.80E-01 | 5.20E-03 | 2.16E-03 | |
|  |  | A8IL38 | CHLREDRAFT_128005 | RabGAP/TBC protein | 1.37 | 1.70 | 1.85 |  | 3.65E-01 | 4.21E-02 | 1.15E-01 | |
|  |  | A8ILF4 | AP2A1 | Alpha-adaptin | 2.46 | 7.45 | 5.88 |  | 3.94E-02 | 3.50E-03 | 4.37E-02 | |
|  |  | A8HRR9 | COPA1 | Alpha-COP | 1.61 | 6.81 | 5.72 |  | 5.33E-01 | 5.06E-03 | 1.57E-02 | |
|  |  | A8IAJ1 | VPS4 | AAA-ATPase of VPS4/SKD1 family | 1.15 | 1.00 | 0.53 |  | 5.02E-01 | 7.83E-01 | 3.65E-02 | |
|  |  | A8HSQ2 | FAP66 | Flagellar associated protein | 0.66 | 2.61 | 2.85 |  | 1.30E-01 | 3.44E-02 | 5.30E-02 | |
|  |  | A8IYH0 | CHLRE_12g549950v5 | Tetraspanning membrane protein SFT2-like protein | 0.69 | 0.21 | 0.12 |  | 7.70E-02 | 5.06E-03 | 1.07E-03 | |
|  |  | A8I023 | TIC110 | 110 kDa translocon of chloroplast envelope inner membrane (Fragment) | 0.42 | 0.39 | 0.44 |  | 9.45E-03 | 1.19E-02 | 2.15E-02 | |
|  |  | Q6Y682 | Rap38 | 38 kDa ribosome-associated protein | 1.16 | 0.37 | 0.10 |  | 1.38E-01 | 7.37E-05 | 6.20E-05 | |
|  |  | A8IA39 | EFG1 | Chloroplast elongation factor G | 0.42 | 0.48 | 0.52 |  | 1.49E-02 | 2.51E-03 | 4.08E-02 | |
|  |  | A8J680 | SECA2 | Chloroplast-associated SecA protein (Fragment) | 0.54 | 0.75 | 0.65 |  | 9.55E-03 | 6.62E-02 | 2.94E-02 | |
|  |  | A8JBT7 | EGY1 | Membrane associated metalloprotease | 0.79 | 0.39 | 0.30 |  | 2.89E-01 | 9.43E-02 | 2.70E-02 | |
|  |  | A8HWL8 | CHLREDRAFT_142189 | Predicted protein | 1.56 | 2.54 | 2.27 |  | 1.46E-01 | 2.35E-02 | 1.56E-02 | |
|  |  | A8J209 | CGL59 | Predicted protein | 0.57 | 0.53 | 0.33 |  | 9.96E-02 | 6.71E-02 | 3.24E-03 | |
|  |  | A8J682 | SECA1 | Protein translocase subunit SecA | 0.45 | 0.69 | 0.83 |  | 1.09E-02 | 6.89E-02 | 3.79E-01 | |
|  |  | Q5S7Y5 | TIM | Triosephosphate isomerase | 2.68 | 4.40 | 3.56 |  | 2.30E-01 | 4.89E-02 | 8.18E-02 | |
| 7 | N- and S- metabolism | A8J6Q7 | SHKA1 | 3-deoxy-D-arabino-heptulosonate 7-phosphate synthetase | 0.31 | 0.62 | 0.57 |  | 3.50E-02 | 1.41E-01 | 1.98E-01 | |
|  |  | A8JH48 | SHKG1 | 3-phosphoshikimate 1-carboxyvinyltransferase | 0.36 | 0.43 | 0.47 |  | 1.15E-03 | 2.78E-03 | 2.62E-03 | |
|  |  | A8J434 | OASTL1 | Cysteine synthase | 0.58 | 0.45 | 0.54 |  | 1.93E-02 | 7.21E-03 | 2.25E-02 | |
|  |  | A8IEE5 | OASTL3 | Cysteine synthase | 5.41 | 4.25 | 4.99 |  | 7.89E-02 | 6.16E-02 | 2.19E-02 | |
|  |  | A8ISA9 | OASTL4 | Cysteine synthase | 17.24 | 18.20 | 17.81 |  | 3.38E-02 | 2.18E-02 | 3.53E-02 | |
|  |  | A8JG03 | LEU1L | Isopropylmalate dehydratase large subunit | 1.97 | 6.80 | 8.04 |  | 1.58E-01 | 2.47E-03 | 2.14E-03 | |
|  |  | A8I9R1 | THD1 | Threonine deaminase | 0.42 | 0.40 | 0.50 |  | 5.07E-03 | 6.49E-03 | 3.53E-02 | |
|  |  | A8IFZ9 | MAA7 | Tryptophan synthase beta subunit | 0.60 | 0.81 | 0.58 |  | 1.45E-02 | 4.77E-02 | 8.29E-02 | |
|  |  | A8J129 | AST1 | Aspartate aminotransferase | 0.59 | 0.62 | 0.33 |  | 1.95E-01 | 2.09E-01 | 4.36E-02 | |
|  |  | A8I263 | AST3 | Aspartate aminotransferase | 2.01 | 2.14 | 2.32 |  | 1.04E-01 | 2.76E-04 | 3.30E-02 | |
|  |  | A8IAK9 | PYR2 | Aspartate carbamoyltransferase | 0.43 | 0.26 | 0.33 |  | 1.36E-01 | 4.94E-02 | 8.04E-02 | |
|  |  | Q7XXT3 | gdh | Glutamate dehydrogenase | 8.04 | 14.61 | 17.62 |  | 5.11E-02 | 3.34E-02 | 1.01E-02 | |
|  |  | A8JGD1 | GDH2 | Glutamate dehydrogenase | 14.42 | 27.91 | 31.36 |  | 4.37E-04 | 2.15E-04 | 6.02E-04 | |
|  |  | A8J3Q6 | APK1 | Adenylyl-sulfate kinase | 3.70 | 4.53 | 3.82 |  | 7.24E-03 | 1.60E-02 | 2.31E-04 | |
|  |  | A8IXF1 | ATS1 | ATP-sulfurylase | 2.00 | 6.83 | 7.68 |  | 3.94E-01 | 1.95E-02 | 2.85E-02 | |
|  |  | A8I3V3 | ATS2 | ATP-sulfurylase | 14.22 | 33.32 | 36.36 |  | 2.98E-02 | 7.02E-03 | 6.98E-03 | |
|  |  | A8JFW4 | DXS1 | 1-deoxy-D-xylulose 5-phosphate synthase (Fragment) | 0.31 | 0.20 | 0.19 |  | 2.71E-03 | 1.03E-03 | 1.53E-03 | |
|  |  | A8ILN4 | HDS1 | 1-hydroxy-2-methyl-2-(E)-butenyl 4-diphosphate synthase | 0.54 | 1.12 | 1.22 |  | 3.99E-03 | 5.88E-01 | 3.36E-01 | |
|  |  | A8IJL3 | CHLRE_12g503550v5 | 2-C-methyl-D-erythritol 24-cyclodiphosphate synthase | 3.88 | 6.20 | 5.70 |  | 9.18E-03 | 1.95E-03 | 1.45E-02 | |
|  |  | A8J0V1 | CMK1 | 4-diphosphocytidyl-2-C-methyl-D-erythritol kinase | 0.87 | 0.36 | 0.24 |  | 4.94E-01 | 2.18E-02 | 1.52E-02 | |
|  |  | A8IMN5 | CMPS1 | Carbamoyl phosphate synthase small subunit | 1.46 | 1.43 | 1.33 |  | 1.61E-01 | 2.38E-02 | 6.28E-02 | |
|  |  | A8JBN5 | PYR4 | Dihydropryrimidine dehydrogenase | 1.23 | 3.39 | 3.95 |  | 8.19E-01 | 5.44E-02 | 2.14E-02 | |
|  |  | A8HQF3 | FAK1 | ODA5-associated flagellar adenylate kinase | 0.48 | 1.18 | 0.72 |  | 3.12E-03 | 7.21E-01 | 3.18E-01 | |
|  |  | A8HN92 | PYR5 | Uridine 5'-monophosphate synthase | 1.35 | 2.97 | 2.19 |  | 5.01E-01 | 1.33E-02 | 5.98E-03 | |
| 8 | ATP hydrolysis coupled proton transport | Q96550 | atpA | ATP synthase subunit alpha | 3.23 | 3.60 | 2.66 |  | 3.79E-02 | 3.56E-02 | 1.10E-01 | |
|  |  | A8HX15 | CHLREDRAFT_187139 | Sodium/potassium-transporting ATPase alpha subunit | 5.75 | 7.33 | 7.63 |  | 4.32E-02 | 3.30E-04 | 1.70E-03 | |
|  |  | A8I164 | ATPvA1 | Vacuolar ATP synthase subunit A | 2.48 | 5.57 | 4.19 |  | 4.17E-01 | 1.72E-02 | 4.95E-02 | |
|  |  | A8IA45 | ATPvB | Vacuolar ATP synthase subunit B | 3.16 | 2.97 | 1.59 |  | 2.29E-02 | 4.28E-02 | 3.25E-01 | |
|  |  | A8IW47 | ATPvE | Vacuolar ATP synthase subunit E | 2.52 | 2.85 | 1.99 |  | 8.14E-03 | 7.69E-03 | 3.96E-02 | |
|  |  | A8HQ97 | ATPvH | Vacuolar ATP synthase subunit H | 3.17 | 8.94 | 7.24 |  | 1.56E-01 | 3.52E-02 | 2.97E-02 | |
|  |  | A8IDY0 | ATPvD1 | Vacuolar H+ ATPase V0 sector subunit D | 2.38 | 4.44 | 2.60 |  | 1.03E-01 | 3.68E-02 | 7.71E-03 | |
|  |  | A8HYU2 | ATPvC | Vacuolar H+ ATPase V1 sector subunit C | 2.54 | 4.82 | 3.38 |  | 1.87E-01 | 4.76E-02 | 7.18E-03 | |
|  |  | A8IST3 | ATPvA3 | Vacuolar proton ATPase subunit A | 1.18 | 3.69 | 3.38 |  | 5.66E-01 | 5.36E-02 | 3.82E-02 | |
|  |  | A8J1K0 | ATPvA2 | Vacuolar proton translocating ATPase subunit A | 4.24 | 7.42 | 5.63 |  | 7.28E-03 | 3.36E-04 | 2.32E-03 | |
| 9 | Response to cytokinin | A8J3F8 | CHLRE_16g672750v5 | Predicted protein | 0.70 | 0.24 | 0.19 |  | 7.35E-02 | 4.33E-03 | 4.27E-03 | |
|  |  | Q70DX8 | S1 | Plastid ribosomal protein S1 | 0.33 | 0.08 | 0.07 |  | 2.00E-02 | 3.55E-03 | 3.42E-03 | |
|  |  | A8JD33 | CHLREDRAFT_153076 | Predicted protein | 1.74 | 2.44 | 1.98 |  | 1.76E-01 | 1.58E-02 | 1.21E-01 | |
|  |  | A8IWA6 | GSN1 | Glutamate synthase NADH-dependent | 1.60 | 5.62 | 4.40 |  | 7.26E-01 | 2.23E-02 | 4.60E-03 | |
|  |  | A8HWZ6 | PRPL13 | Plastid ribosomal protein L13 | 0.43 | 0.12 | 0.15 |  | 3.68E-02 | 1.08E-02 | 1.08E-02 | |
|  |  | A8IU62 | PIN3 | Peptidyl-prolyl cis-trans isomerase parvulin-type | 0.74 | 0.50 | 0.45 |  | 9.70E-02 | 1.24E-02 | 4.89E-02 | |
|  |  | A8HVJ9 | CHLREDRAFT_112806 | Photosystem II stability/assembly factor HCF136 | 0.24 | 0.09 | 0.10 |  | 1.33E-03 | 5.74E-04 | 5.72E-04 | |
|  |  | A8J503 | PRPL6 | Plastid ribosomal protein L6 | 0.79 | 0.20 | 0.15 |  | 1.58E-01 | 3.33E-03 | 2.03E-03 | |
|  |  | Q8HTL1 | rpl5 | 50S ribosomal protein L5 chloroplastic | 1.33 | 0.64 | 0.52 |  | 2.64E-01 | 6.14E-03 | 5.84E-04 | |
|  |  | A8JAL6 | PRPL15 | Plastid ribosomal protein L15 | 0.33 | 0.05 | 0.05 |  | 2.04E-02 | 5.06E-03 | 5.00E-03 | |
|  |  | A8J785 | ATPG | ATP synthase subunit b' chloroplastic | 0.61 | 0.26 | 0.33 |  | 1.51E-01 | 4.60E-03 | 3.82E-02 | |
|  |  | A8IYP4 | PRK1 | Phosphoribulokinase | 0.81 | 0.08 | 0.06 |  | 3.61E-01 | 2.68E-03 | 2.43E-03 | |
|  |  | A8J8U1 | RPN10 | 26S proteasome regulatory subunit | 0.84 | 0.68 | 0.57 |  | 8.64E-02 | 7.18E-03 | 1.47E-02 | |
|  |  | A8I8X2 | DEG1A | DegP-type protease | 2.45 | 1.38 | 1.01 |  | 3.97E-03 | 3.79E-01 | 9.50E-01 | |
|  |  | A8IKQ0 | FBP1 | Fructose-1,6-bisphosphatase | 1.11 | 0.52 | 0.35 |  | 6.64E-01 | 1.86E-02 | 6.54E-03 | |
|  |  | A8INR7 | PRPL27 | Plastid ribosomal protein L27 | 0.19 | 0.05 | 0.04 |  | 2.00E-03 | 8.47E-04 | 7.99E-04 | |

**Supporting information Table S4.** List of primers used in this work.

| Primers | Nucleotide sequences (5’ to 3’) |
| --- | --- |
| *LHCA2*-F | GAAGAGCGAGGAGATGAAGC |
| *LHCA2*-R | CCGACGAGGTGTAGATGTTG |
| *LHCA5*-F | AGACCAAGGAGATCAAGAACGG |
| *LHCA5*-R | TGCAAGTGCCGATGTTCTTC |
| *LHCA6*-F | TGATGCTGGTTGCCAAGAAC |
| *LHCA6*-R | TTGAACCACTTCAGGGACTCG |
| *LHCA7*-F | TCATCCTCACCTCCATTGGT |
| *LHCA7*-R | TTCTTGAAGTCGTACCAGCG |
| *LHCA9*-F | TTCATCAACTCCTTCCCCTTCG |
| *LHCA9*-R | AGGTGATGTTCTTGCCGAAG |
| *CBLP*-F | ATGACCACCAACCCCATCATC |
| *CBLP*-R | GGTCCCACAGCATGGCAATG |
